# Spatial metatranscriptomics resolves host–bacteria–fungi interactomes

**DOI:** 10.1038/s41587-023-01979-2

**Published:** 2023-11-20

**Authors:** Sami Saarenpää, Or Shalev, Haim Ashkenazy, Vanessa Carlos, Derek Severi Lundberg, Detlef Weigel, Stefania Giacomello

**Affiliations:** 1 SciLifeLab, Department of Gene Technology, KTH Royal Institute of Technology, Stockholm, Sweden; 2 Max Planck Institute for Biology Tübingen, Tübingen, Germany; 3 Cluster of Excellence Physics of Life, TU Dresden, Dresden, Germany; 4 Institute for Bioinformatics and Medical Informatics, University of Tübingen, Tübingen, Germany; 5 Present Address: Systems Biology of Microbial Communities, University of Tübingen, Tübingen, Germany; 6 Present Address: Swedish University of Agricultural Sciences, Uppsala, Sweden

**Keywords:** Transcriptomics, Metagenomics, Microbiome, Microbe

## Abstract

The interactions of microorganisms among themselves and with their multicellular host take place at the microscale, forming complex networks and spatial patterns. Existing technology does not allow the simultaneous investigation of spatial interactions between a host and the multitude of its colonizing microorganisms, which limits our understanding of host–microorganism interactions within a plant or animal tissue. Here we present spatial metatranscriptomics (SmT), a sequencing-based approach that leverages 16S/18S/ITS/poly-d(T) multimodal arrays for simultaneous host transcriptome- and microbiome-wide characterization of tissues at 55-µm resolution. We showcase SmT in outdoor-grown *Arabidopsis thaliana* leaves as a model system, and find tissue-scale bacterial and fungal hotspots. By network analysis, we study inter- and intrakingdom spatial interactions among microorganisms, as well as the host response to microbial hotspots. SmT provides an approach for answering fundamental questions on host–microbiome interplay.

## Abstract (web summary)

Interactions among plant hosts, bacteria and fungi are measured at high spatial resolution.

## Main

Advances in spatially resolved transcriptomics technologies have greatly improved the understanding of eukaryotic host gene expression mechanisms in animal and plant tissues^1–4^. These technologies have been designed to capture targeted^3,5,6^ or untargeted^1,2,4^ RNA information based on imaging or sequencing of unique molecules, enabling the study of hundreds of genes or the whole transcriptome, respectively.

Spatial variation is also prominent in host–microorganism interactions, and single-cell RNA-sequencing (scRNA-seq) of the host has been used to understand how this affects host cellular responses during infection^7^. However, integrated spatially resolved analyses of microbial identity and the host response remain rare and are typically focused on individual microbial taxa within a host^8^. With existing technology, it has not been possible to simultaneously resolve the spatial interactions between a host and the multitude of microorganisms colonizing it. This has considerably limited our understanding of host–microorganism interactions at the tissue level.

Microorganisms often live in diverse communities surrounded by other microorganisms. Both cooperative and antagonistic interactions between microorganisms are known to be important for the functionality and health of ecosystems, plants, animals and humans^9–11^. Moreover, the success of microbial colonization and infection depends strongly on the spatial structure of microbial interactions with other microorganisms and with multicellular species, and several pioneering studies have revealed clear and functionally significant spatial organization in host-associated microbial communities^12–14^. Much broader knowledge of the spatial organization of microorganisms within hosts, and the associated local host responses, is therefore needed to fully understand the biology of the host–microorganism–microorganism interactome.

Fluorescence in situ hybridization (FISH)-based techniques provided the first insights into microbial spatial organization in different environments^15^ and in host tissues, including mouse gut^16^, human plaque microfilms^16^ and *Arabidopsis thaliana* roots^17^. A limitation of these targeted methods is that they use a set of predesigned probes, each specific to a single microbial taxon. Current FISH-based technologies thus cannot provide comprehensive spatial descriptions of unknown microbiomes. Moreover, despite recent advances, these methods cannot yet achieve complete spatial resolution of the host’s expression patterns due to their limited capacity and overfitting to specific hosts^18^.

Plants are colonized by a heterogeneous set of microorganisms whose diversity is comparable to that of the human gut’s microbial population^19^. Similar to gut microorganisms, plant colonizing microorganisms affect the host’s health and physiology in various ways, ranging from beneficial^20^ to harmful^21,22^. Plant microbial communities are shaped in an environment-dependent manner by the intertwined forces of host–microorganism and microorganism–microorganism interactions, which ultimately determine the fitness of the host and the associated microorganisms^23^.

Because of the limitations of current analytical methods, microbial interactions within plants are often deduced from complete tissues or whole plants, based on 16S rDNA abundance data^9,24,25^. This approach inevitably makes it impossible to resolve microscale differences in abundance. Hence, bulk RNA-seq can only be used to study average plant–microorganism interactions in a tissue^26,27^. Given the tremendous variation of unique RNA profiles found within tissues, demonstrated repeatedly by spatial transcriptomic (ST) and scRNA-seq analyses^2,28^, it is very likely that important information has been obscured by the limited spatial resolution of the techniques used to study plant–microorganism interactions.

**Fig. 1:**
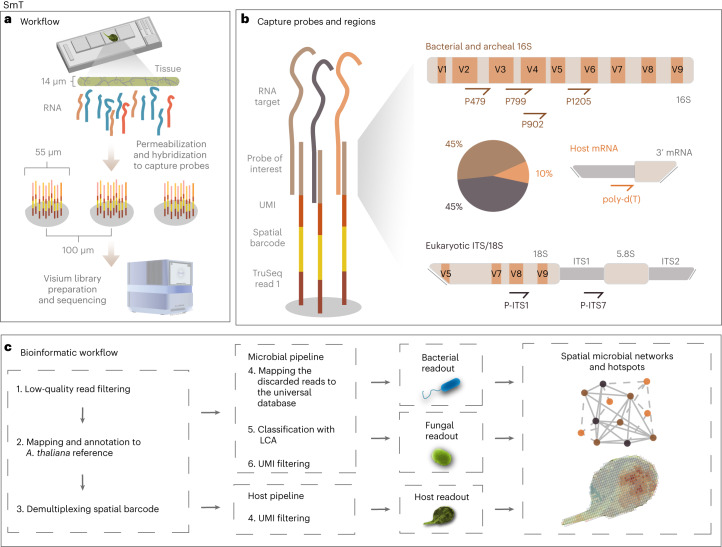
Overview of the method. **a**, SmT uses capture arrays on glass slides. Each capture array contains 4,992 spots that are 55 µm in diameter and 100 µm from center to center. Cells are permeabilized to release RNA molecules that hybridize to the barcoded capture probes in the spots. The captured molecules are then processed into a sequencing library. **b**, Capture probes consist of a sequencing adapter, a spatial barcode, a UMI and a capture moiety. Polyadenylated mRNAs are captured with poly-d(T) probes that comprise 10% of all the capture probes. Ribosomal RNAs from fungi are captured with P-ITS7 and P-ITS1 probes targeting the 18S rRNA and ITS regions, respectively. Ribosomal RNAs from bacteria and archaea are captured with P479, P799, P902 and P1205 probes targeting bacterial 16S rRNA. Bacterial and archaeal probes and fungal probes each comprise 45% of the capture probes. **c**, A bioinformatic workflow designed to assign the reads to host or microbial modalities. First, low-quality reads are filtered out, the remaining reads are mapped against the *A.* *thaliana* TAIR10 reference genome, and spatial barcodes are demultiplexed. Second, mapped *A. thaliana* reads are filtered based on their UMI and compiled to obtain a gene-count matrix. Third, the reads not mapping to *A. thaliana* are mapped to a universal database to remove those that are not clearly of microbial origin. The remaining microbial reads are classified with LCA based on their identity and UMIs, and unique taxa are counted to generate separate unique taxa-count matrices for fungi and for bacteria and archaea.

Here we present spatial metatranscriptomics (SmT; Fig. 1), an untargeted approach that allows simultaneous interrogation of bacterial and fungal communities, and the corresponding host transcriptional responses with a spatial resolution of 55 µm. By capturing the spatial distribution of bacterial and archaeal 16S rRNA sequences, together with fungal internal transcribed spacer (ITS) and 18S rRNA sequences and the host mRNAs, we link local changes in host gene expression to the size and composition of local microbial populations in *A. thaliana* leaves. We resolve the organization of microbial communities along tissue sections and demonstrate the presence of microbial hotspots at the leaf scale, and how these locally impact host responses.

## Results

### Spatial detection of bacterial infection and host response

**Fig. 2:**
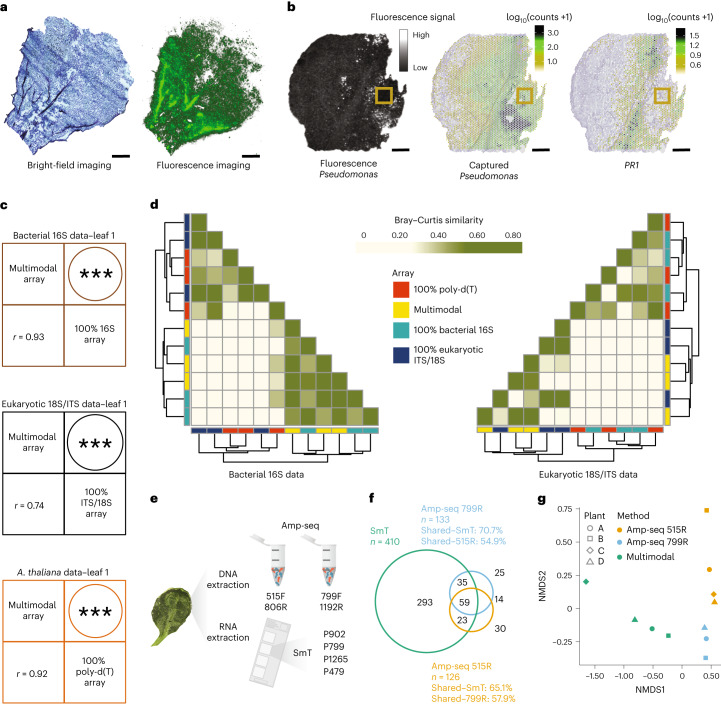
SmT resolves the microbial profile and host transcriptome at microscopic resolution. **a**, A Toluidine blue-stained bright-field image of a 14-µm thick longitudinal *A. thaliana* leaf section (left) and the fluorescent cDNA footprint (right) of the same section from the tissue optimization experiment. **b**, Left, fluorescence image of an intact *A. thaliana* leaf syringe-infiltrated (yellow square) with mCherry-tagged Pst DC3000 bacteria. Middle, a 14-µm thick longitudinal section from the same leaf was analyzed using a 50% poly-d(T), 25% P799 and 25% P902 array, revealing the spatial capture of Pst DC3000 16S rRNA molecules. Right, spatial distribution of *PR1* gene expression in the same leaf section. Scale bars: 1 mm. **c**, Pearson correlation coefficient and the corresponding two-tailed significance test of bacterial 16S rRNA, eukaryotic 18S rRNA/ITS and *A. thaliana* molecules captured in leaf 1 with a multimodal array containing 10% poly-d(T), 45% 16S rRNA and 45% 18S rRNA/ITS probes and with 100% 16S rRNA or 18S rRNA/ITS and poly-d(T) arrays. In all correlations, *P* = 0. **d**, Bray–Curtis similarity for bacterial and fungal taxa captured on different arrays, organized by hierarchical clustering. **e**, Experimental validation of SmT by amplicon sequencing. **f**,**g**, Numbers of bacterial and archaeal taxa detected using the two methods in a representative sample of four leaves from two plants are compared qualitatively using a Venn diagram (**f**) and quantitatively using NMDS (**g**). NMDS, non-metric multidimensional scaling.

To determine whether mRNA molecules could be captured from the host *A. thaliana* leaf sections while preserving the tissue’s morphology, we applied an optimized ST protocol to leaves grown under laboratory conditions^29^. To this end, we permeabilized a 14-μm thick longitudinal leaf section on a glass surface uniformly coated with poly-d(T) capture probes. Following cDNA synthesis with fluorophores, we obtained a fluorescent cDNA footprint (Fig. 2a) whose morphology matched to that of the original leaf, demonstrating that spatial host gene expression patterns can be obtained from longitudinal leaf sections. Next, because bacterial communities are typically characterized based on 16S rDNA sequences, we hypothesized that capturing 16S rRNA molecules could provide information on the spatial distribution of bacteria in host tissues. To prove this concept, we analyzed leaves of lab soil-grown *A. thaliana* plants infiltrated with the model pathogen *Pseudomonas syringae* pv. *tomato* DC3000 (Pst DC3000), which was genetically labeled to enable its fluorescence imaging in whole leaves (Fig. 2b). The array used in the analysis contained two degenerate probes (P799 and P902) to capture bacterial and archaeal (hereafter ‘bacterial’) diversity from 16S rRNA hypervariable regions, together with poly-d(T) probes to capture host mRNA, mixed in the following proportions: 50% poly-d(T), 25% P799 and 25% P902.

We imaged intact infected leaves 3 d postinfiltration to record the fluorescent spatial pattern of the bacterial infiltration and analyzed corresponding 14-µm-thick tissue sections with the array described above (Fig. 2b). We detected a uniform host and bacterial molecular capture throughout the tissue section (Supplementary Fig. 1a,b), indicating successful tissue permeabilization and RNA hybridization. We identified 512,779 unique bacterial molecules, of which 92.4% corresponded to *Pseudomonas*, indicating the controlled infection system of our lab-grown leaves. The density of *Pseudomonas* 16S rRNA molecules was the highest around the infiltration site (yellow squares in Fig. 2b) and gradually declined toward distal regions, thus providing a more comprehensive picture than the fluorescence imaging, which had missed the spatial component of the infection gradient (Supplementary Figs. 1c and 2a–c). This is confirmed by the positive correlation (*r* = 0.21, *P* < 2.2 × 10^−16^; Supplementary Fig. 1d) between the *Pseudomonas* array signal and the *Pseudomonas* fluorescence signal, which indicates higher sensitivity of the array in capturing the decreasing gradient of microbial content from the infection site that could not be recognized based on the fluorescence signal (Fig. 2b and Supplementary Figs. 1d and 2d). A similar pattern was seen in another leaf replicate (Supplementary Fig. 2f,g,i).

We next investigated the expression patterns of host genes in relation to *Pseudomonas* localization. Following a machine learning-based analysis (‘Boruta’^30^), we found the expression of *pathogenesis-related gene 1* (*PR1*, typical marker of the plant immune response^30^) as the most associated with *Pseudomonas* localization in both replicates (Supplementary Table 1). As expected, the *PR1* spatial gene expression pattern closely matched the distribution of SmT-derived *Pseudomonas* signal (Fig. 2b and Supplementary Fig. 2c,h), significantly correlated with the *Pseudomonas* signal detected by the array (*r* = 0.52, *P* < 2.2 × 10^−16^; Supplementary Fig. 1e) and was nearly fully contained within the region where *Pseudomonas* was detected by the array (Supplementary Fig. 1f). Finally, we found spatial colocalization of fluorescent microscopy-derived *Pseudomonas* signal with the SmT-derived *Pseudomonas* signal and host *PR1* immune gene expression (Supplementary Fig. 2e,j). These spatial patterns, which are obvious from visual inspection, were validated by statistical hotspot analysis, in which only significant spatial heterogeneities are considered (Supplementary Fig. 3). Taken together, these results show that we are able to simultaneously capture bacterial taxonomic information and host transcripts.

### Simultaneous detection of microbial and host spatial data

Having demonstrated that bacterial information can be specifically captured together with information on host gene expression, we aimed to add a third modality to our arrays, capturing information from eukaryotic microorganisms, specifically fungi. We designed 18S rRNA/ITS probes specific for fungi and tested their performance in both separate arrays and a multimodal array. For this purpose, we created arrays with 100% poly-d(T) probes, 100% 16S rRNA probes and 100% 18S rRNA/ITS probes, as well as a multimodal array containing all three probe types (10% poly-d(T), 45% 16S rRNA and 45%18S rRNA/ITS). We dissected three leaves of outdoor-grown *Arabidopsis* plants into four 14-μm thick longitudinal sections and analyzed consecutive sections from each leaf using the four array types. The multimodal and unimodal arrays greatly enriched the proportion of captured reads for the corresponding taxa when compared to the unspecific poly-d(T) probes (Supplementary Fig. 4). Specifically, at the genus level, the multimodal array enriched bacterial and fungal unique molecules up to ~19- and ~31-fold, respectively. At the superkingdom level, the 100% 16S rRNA array enriched bacterial-unique molecules up to ~47-fold when compared to the 100% poly-d(T) array, and the 100% 18S rRNA/ITS array enriched fungal unique molecules up to ~233-fold. As expected, the multimodal array enriched microbial signals to a lesser degree than the 100% 16S rRNA and 100% 18S rRNA/ITS arrays, given the lower concentration of microorganism-specific probes in the multimodal arrays (Supplementary Fig. 4).

Importantly, the bacterial information captured using the multimodal arrays was almost identical to that captured from consecutive tissue sections using 100% 16S rRNA arrays (both qualitatively and quantitatively). The multimodal array captured up to 962 bacterial taxa and 179 fungal taxa at the genus level (Supplementary Table 2), and recapitulated the profile of 100% 16S rRNA arrays independently if full bacterial components (*r* = 0.91–0.93, *P* < 0.001), top 500 bacterial taxa (*r* = 0.92–0.93, *P* < 0.001) or top 20 bacterial taxa (*r* = 0.96–0.99, *P* < 0.001) were considered (Fig. 2c and Supplementary Figs. 5–8). Similarly, the multimodal array recapitulated the profile of 100% 18S rRNA/ITS arrays if full fungal components (*r* = 0.71–0.74, *P* < 0.001) were considered, while the correlations obtained for the top 500 and 20 fungal taxa were on average 0.71 and 0.77 (*P* < 0.001), respectively (Fig. 2c and Supplementary Figs. 9–12).

Bray–Curtis similarity showed that the bacterial profile obtained using the bacterial 16S rRNA array was most similar to that of the multimodal array, while the fungal profile obtained with the multimodal array clustered with that for the eukaryotic 18S rRNA/ITS array (Fig. 2d). Conversely, the bacterial profile obtained with the eukaryotic 18S rRNA/ITS array and the poly-d(T) array differed markedly from that obtained with the bacterial 16S rRNA array, and the fungal profile obtained with the bacterial 16S array and the poly-d(T) array differed markedly from that obtained with the 18S rRNA/ITS array. By downsampling the 100% 16S rRNA and 18S rRNA/ITS arrays, simulating various probe concentrations, we identified that the Shannon diversity index was almost entirely saturated at 45% simulated probe concentration in all samples for both array types, showing that no new information could be captured by increasing the probe microbial concentrations (Supplementary Fig. 13). When a kingdom-specific array was used to analyze a kingdom other than that for which it was designed, it failed to do so (Fig. 2d). We confirmed this result by calculating the Shannon diversity index across leaves, revealing that the multimodal and 100% 16S rRNA arrays captured similar levels of diversity (*H*′ = 3.62–4.01 and *H*′ = 3.81–4.04, respectively), different from the 100% 18S rRNA/ITS and 100% poly-d(T) arrays (*H*′ = 2.76 and *H*′ = 3.70, respectively; Supplementary Fig. 14). Overall, the bacterial profile captured by the 16S rRNA array and the fungal profile captured by the 18S rRNA/ITS array could only be recapitulated by the multimodal array and not by any of the unspecific probes (Fig. 2d). These results imply that the multimodal array quantitatively enriched microbial counts and accurately profiled microbial populations within tissue sections, unlike the unspecific poly-d(T) probes (Fig. 2d, Supplementary Figs. 4–14 and Supplementary Table 2).

Finally, we confirmed that the multimodal array correctly captured the transcriptomic profile of the host as well (Fig. 2c and Supplementary Figs. 15–17) by comparing the *A. thaliana* gene expression pattern captured with the multimodal array to that obtained with the 100% poly-d(T) array. The multimodal array captured 16,368 *Arabidopsis* genes on average and its correlation with the 100% poly-d(T) array was high (*r* = 0.92–0.93, *P* < 0.001). Overall, these results show that multimodal arrays enable accurate simultaneous capture of the host transcriptome, the bacterial profile and the fungal profile.

### Validation of the multimodal array with amplicon sequencing

As each of the two 16S rRNA probes captures a slightly different bacterial community, we introduced two additional 16S rRNA probes, P479 and P1265 (Supplementary Figs. 18 and 19 and Fig. 1), thus improving the ability of the multimodal array to capture the bacterial taxonomic range. We compared the results of this multimodal array with those from 16S rDNA amplicon sequencing (amp-seq)—current gold standard for bacterial profiling. Amp-seq involves PCR amplification of crude DNA extracts using a primer pair. Conversely, our multimodal array captures RNA fragments that are targeted by individual probes. To be able to directly compare the multimodal array to amp-seq—which is conducted on crude extracts—we sampled four leaves from field-grown *A. thaliana* plants and simultaneously extracted their RNA and DNA. We then analyzed the crude RNA extracts with the multimodal array containing the additional P479 and P1265 probes and used the extracted DNA for amp-seq of two 16S loci with V3-V4 (primers 515F + 806R) and V4-V6 (primers 799F + 1192R; Fig. 2e).

We first qualitatively compared the bacterial profiles obtained using the multimodal array to those obtained with the two single pairs of 16S rDNA amp-seq primers by analyzing the presence or absence of every genus found by at least one of the three processes (Fig. 2f and Supplementary Fig. 20). SmT detected more than three times the total number of bacterial taxa detected by the two amp-seq primer pairs (Fig. 2f), including ~71% of the taxa detected by the amp-seq V4-V6 primers and ~65% of the taxa detected by the amp-seq V3-V4 primers. The two amp-seq primer pairs overlapped in ~56% of detected taxa.

We obtained similar results for the other three biological replicates (Supplementary Fig. 20), and a similar trend in quantitative analyses, comparing the Bray–Curtis distances, based on relative abundances (Fig. 2g). Furthermore, pairwise Spearman correlations calculated on bacterial profiles of genera shared across each pair of possible comparisons between the three profiles (Supplementary Fig. 21) showed that SmT delivers an accurate quantitative microbial profile, comparable to amp-seq. In summary, these results confirm that our multimodal array accurately profiles bacteria in *A. thaliana* leaves and captures a more diverse taxonomic range than standard amplicon sequencing.

### Microbial hotspots in the leaf govern microbial interactions

**Fig. 3:**
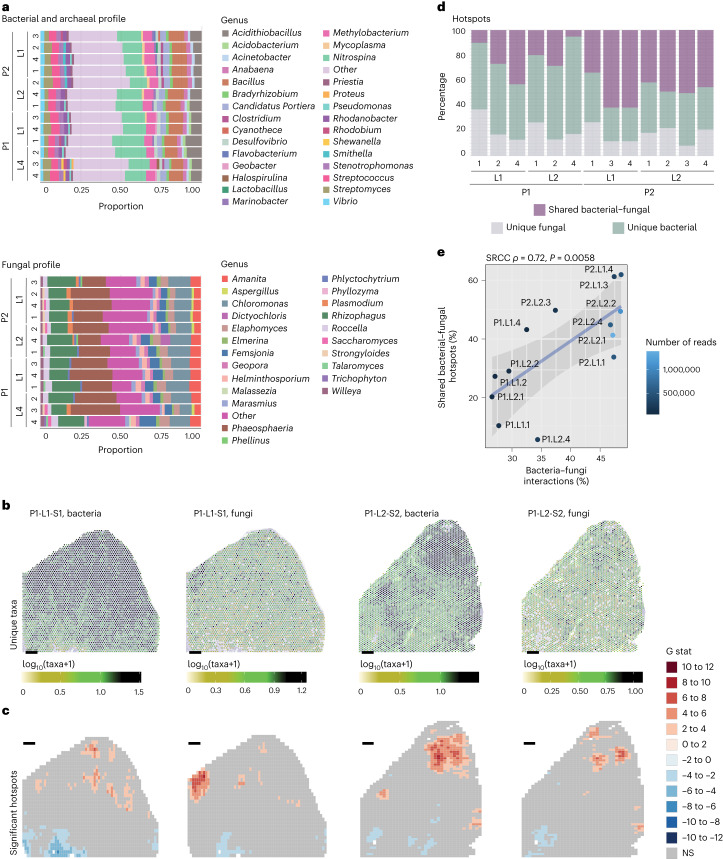
Microbial interactions are driven by spatial organization. **a**, Bacterial and fungal profiles for each of the sections of four leaves (‘L’) from two plants (‘P’). ‘Other’ denotes binned bacterial and archaeal genera and fungal genera having ≤1% abundance. **b**, Numbers of unique microbial taxa per capture spot. **c**, Significant hot- and cold-spots for bacteria and fungi in a representative leaf section. NS, not significant. Scale bars: 500 µm. **d**, Percentages of shared and unique hotspots among bacteria and fungi across 13 different leaf sections. Of note is the variance in shared regions across the sections. The sections were taken from two leaves from two different plants (four leaves in total); ‘P’ denotes plant and ‘L’ denotes leaf. **e**, The proportion of interkingdom (bacteria–fungi) interactions as a function of the proportion of shared interkingdom hotspots and the number of reads. SRCC *ρ* = 0.72, *P* = 0.0058 (two-tailed test).

The spatial distribution of the members of natural microbial communities within host leaves has been largely unknown. Therefore, we used SmT to investigate the microbial profiles of different leaf sections in outdoor-grown *A. thaliana* leaves. The microbial profiles of the different sections were similar, reflecting the similarity of the environments in which the source plants were grown (Methods) and the reproducibility of our method (Fig. 3a). We ensured that this similarity is not driven by any environmental contaminants by quantitatively comparing the observed microbial profiles with those of axenically-grown leaves. Despite the axenically-grown leaves presented microorganisms that probably survived the seed surface sterilization (for example, sporulating microorganisms; Methods), we found that both the bacterial and fungal profiles of outdoor- and axenically-grown leaves largely differ (Supplementary Figs. 22–24). In fact, 42% of the axenically-grown leaf microbial relative abundance alone was characterized by one bacterial genus, that is *Paenibacillus* (highly resistant spore-forming bacteria^31^), while the same bacterial genus had an average relative abundance of only 0.035% in the outdoor-grown leaves. Among outdoor-grown leaves, considering only taxa with relative abundances above 1%, we identified 29 bacterial taxa and 23 fungal taxa at the genus level (Supplementary Tables 3 and 4). The relative abundances of different microorganisms did not vary greatly across sections, leaves or whole plants. Analysis of the overall spatial distributions of bacterial and fungal genera (Fig. 3b and Supplementary Fig. 25) revealed that microorganisms were present across almost the entire leaf surface—unique bacterial molecules were detected in 99.9% of sampling spots at an average density of ~277 molecules per million reads, while unique fungal molecules were detected in 97.5% of sampling spots at an average density of ~261 molecules per million reads (Supplementary Table 5 and Supplementary Fig. 25). We validated that this pattern is not a technical artifact of lateral diffusion by comparing the reads under and outside the tissue, finding that for both the microbial and host profiles, the vast majority of reads was derived from under-the-tissue, while also showing a different microbial profile than outside-the-tissue (Supplementary Figs. 26–28).

We next analyzed the geography of microbial colonization. Although we detected both bacteria and fungi across the entire leaf surface, they were concentrated in hotspots rather than being homogeneously distributed (Fig. 3cand Supplementary Fig. 29). Some leaf regions, in 100% of the outdoor-grown leaf sections analyzed, were highly colonized with microorganisms, while others were uncolonized or colonized at very low levels. This complex spatial pattern, instead, could not be observed in sections of axenically-grown leaves where less than half of the tissue sections presented a few small highly delimited hotspots (Supplementary Fig. 30). Moreover, these were almost completely related to only one bacteria, that is *Paenibacillus*^31^ (93% and 83% of the hotspot microbial composition in leaf batches 1 and 2, respectively), in contrast to the mixed and diverse hotspot microbial composition found in outdoor-grown leaves (Supplementary Figs. 31 and 32).

Further investigation of outdoor-grown leaves revealed that some hotspots were shared between bacteria and fungi (Fig. 3d and Supplementary Fig. 29). The relative abundance of shared and unique hotspots varied widely across the 13 leaf sections (Fig. 3d). Because microbial interactions are constrained by physical proximity^32^, we hypothesized that the relative abundance of shared and unique hotspots controls the proportion of interkingdom and intrakingdom interactions. To test this, we computed the interaction network of the 50 most abundant bacterial and fungal taxa using an algorithm that accounts for the spatial structure of our data (Methods). We exemplify this approach by focusing on a subnetwork of 14 taxa (12 bacterial and 2 fungal), which are strongly associated (average pairwise Spearman’s rank correlation coefficient (SRCC) ≥ 0.35) in all tested leaf sections (Supplementary Fig. 33). We then tested the association between the relative abundance of shared hotspots and the magnitude of interkingdom (bacteria–fungi) interactions across the leaf sections, revealing a positive correlation between the two features (SRCC = 0.72, *P* = 0.0058; Fig. 3e). This implies that microbial interactions are driven by their spatial organization, and specifically by their presence in shared hotspots. We found a similar association for the magnitude of bacteria–bacteria interactions and the fraction of bacterial-unique hotspots (SRCC = 0.72, *P* = 0.059; Supplementary Fig. 34), but lower for fungi–fungi interactions and the fraction of fungal-unique hotspots (SRCC = 0.47, *P* = 0.1; Supplementary Fig. 35).

Together, these results demonstrate a considerable spatial organization of microorganisms within the leaf.

### Microbial hotspots and host gene expression associations

**Fig. 4:**
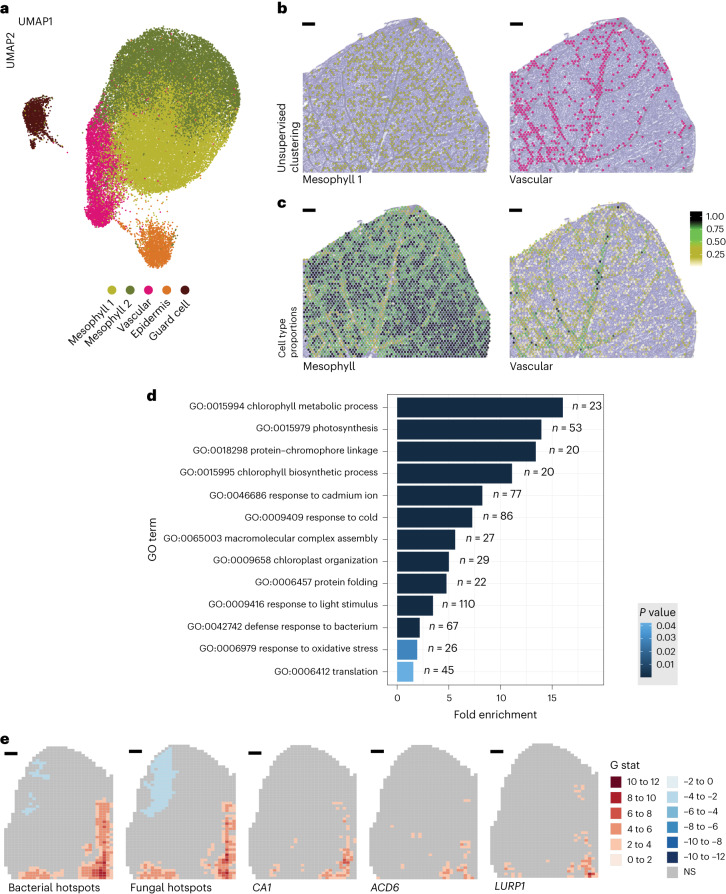
Host response is associated with microbial colonization pattern. **a**, UMAP clustering of host gene expression. **b**, Projection of UMAP clusters mesophyll 1 and vascular on a representative leaf section. **c**, Mesophyll and vascular cell-type proportions projected on a representative leaf tissue section. **d**, Overrepresented GO terms for microbial-associated genes (*n* denotes the number of genes labeled with the indicated GO term). **e**, Spatial distributions of significant bacterial and fungal hotspots together with hotspots for the expression of the defense-related genes *CA1* (AT3G01500), *LURP1* (AT2G14560) and *ACD6* (AT4G14400). NS, not significant. Scale bars: 500 µm.

Because microorganism–microorganism interactions are driven by spatial relatedness, we hypothesized that microbial organization might also drive host–microorganism interactions. We therefore investigated the effects of microbial hotspots on the host transcriptome by reducing the host expression patterns into five cell clusters using uniform manifold approximation and projection (UMAP^33^; Fig. 4a and Supplementary Figs. 36 and 37). As expected, the clustered spots reflected the leaf’s tissue structure, in which different cell types are distributed fairly evenly with the exception of vascular tissue (Fig. 4b and Supplementary Fig. 37). The close proximity of clusters 1 and 2 in UMAP indicates that these cells have similarities in their gene expression patterns as confirmed by the spot deconvolution analysis, which identified most of the spots populated by mesophyll cell types (Fig. 4c, Supplementary Figs. 38–40 and Supplementary Table 6). For example, *chlorophyll a*/*b binding protein 3* (*CAB3*), a common marker gene for mesophyll cells^34^, is upregulated in cluster 2 (avg. log_2_(fold change(FC)) = 0.33; Supplementary Fig. 41). Instead, cluster 3 is populated by both mesophyll and vascular cell types, as its spatial location clearly suggests (Fig. 4b), and in agreement with the spatial expression of the gene *glutathione s-transferase phi 9* (*GSTF9*; Supplementary Fig. 41). Clusters 4 and 5, in addition to mesophyll cell types, presented epidermal cell types (Supplementary Fig. 40), while cluster 5 contained the putative guard cell-type-marker gene *AT2G31141* (ref. ^35^; avg. log_2_(FC) = 1.05; Supplementary Fig. 41).

Overall, these results show that our system accurately resolves spatial host expression profiles in leaves. However, gene annotation analysis revealed no strong association between any of the five clusters and microbial colonization, and there was no obvious visual overlap between the clusters and the microbial hotspots (Supplementary Figs. 29 and 37).

To further investigate the host response to microbial hotspots, we first tested what fraction of expression hotspots overlapped with the microbial hotspots. We found that it highly varies across leaf sections, ranging from 4.6 to 75% shared expression-microbial hotspots (Supplementary Fig. 42). Next, we performed a machine learning-based analysis (‘Boruta’) to associate the host’s spatial gene expression pattern with bacterial and fungal abundance (Methods). This revealed 1,323 and 954 host genes that were significantly associated with bacteria and fungi, respectively (Supplementary Table 7). To test how general our results are, we asked how often genes were associated with microbial abundance in at least two sections of the same leaf (Supplementary Fig. 43). While moving from one section per leaf to two sections per leaf substantially reduced the number of genes significantly associated with microbial abundance, the size of this gene set was more moderately reduced when requiring that a gene was significant in three sections per leaf (Supplementary Table 8). This behavior implies that the chosen cutoff enriches real biological signal. This conservative approach reduced the number of genes associated with bacteria and fungi to 645 and 442, respectively, thus filtering singular hits. The vast majority of these (63% of 667 genes in total) was associated with both kingdoms, indicating involvement in a general microbial response by the host, rather than a kingdom-specific one (Supplementary Fig. 44). A gene ontology (GO) analysis revealed enrichment of biological process terms associated with plant immune responses, including GO:0042742—‘defense response to bacterium’ and GO:0006979—‘response to oxidative stress’ (Fig. 4d, Supplementary Fig. 45 and Supplementary Table 9). In total, 73 (11%) of the associated genes had GO terms associated with defense responses to bacteria and/or fungi (Supplementary Table 10). The spatial correlation between gene expression and microbial abundance is well illustrated by the expression patterns of the following three genes: *ACD6*, *CA1* and *LURP1* (Fig. 4e and Supplementary Fig. 46). All three genes are related to basal plant immunity—*ACD6* is broad-spectrum disease resistance gene activated by diverse microorganisms^36^, the *CA1* gene product binds the immune-related hormone salicylic acid^37^ and regulates stomatal opening during pathogen invasions^38^ and *LURP1* is required for resistance to the pathogen *Hyaloperonospora parasitica*^39^. Overall, these results reveal a connection between the spatial organization of microorganisms within the leaf and the host expression signature.

## Discussion

We present SmT, a multimodal untargeted sequencing method to investigate host–microorganism–microorganism interactions in tissue sections at a resolution of 55 µm. Numerous spatially resolved transcriptomics methods have been introduced so far^40^ based on either targeted^3,41^ or untargeted^1,4,42^ capture of the transcriptional information and characterized by different spatial resolutions ranging from subcellular^43–45^ to multiple cells^1,46^. These methods have been applied to a wide range of tissues from humans^47–49^ to plants^2,45,50,51^. Recently, methods have been developed that are capable of detecting multiple modalities such as protein and transcriptional information^41,46,52–54^ or chromatin accessibility and transcriptional information^55^. In addition, ref. ^56^ presented spatial capture of bacterial information in human cancer tissue. Our SmT approach extends these recent efforts by capturing information not only from the host and its colonizing prokaryotic microorganisms but also from its colonizing eukaryotic ones, thus achieving to retrieve spatial information from three different coexisting organisms simultaneously by using a diverse set of probes specific for polyadenylated transcripts, 16S rRNA and 18 rRNA/ITS regions.

SmT captures fungal, bacterial and host signals from a tissue section while preserving their spatial structure and thus enabling integrated network analysis of gene expression by the host and its microbiota. Recent advances in smFISH techniques for microbiome analysis support the spatially resolved capture of over 1,000 bacterial taxa at the single-cell level^16^ or the detection of bacterial metabolic activities^15^. However, smFISH is a laborious technique and requires the design of highly sensitive probes to capture a sample’s full bacterial diversity. Extending it to simultaneous detection of host whole-transcriptome information and potentially another microbial kingdom will likely be very challenging. SmT provides a straightforward approach, by sequencing the 16S rRNA and 18S rRNA/ITS variable regions together with polyadenylated transcripts. Our validation of SmT with amplicon sequencing, the gold standard method for bacterial profiling, revealed that SmT can more sensitively capture bacterial diversity than amplicon sequencing. This improvement is probably related to the usage of four individual probes simultaneously, compared with a set of two primers, providing a more diverse set of captured molecules. Similarly to our results, 16S amplicon sequencing was unable to detect many rare bacterial taxa in soil samples, probably due to primers bias^57^. Nevertheless, like any other emerging technologies, SmT presents limitations. The higher sensitivity of SmT comes with an increased risk of capturing signals from environmental contamination. As we have shown, this risk can be mitigated by exploiting the spatial information associated with each read. Specifically, by focusing on hotspots, contrasting the profiles under and outside the tissue and comparing different sections of the same sample, we were able to highlight fundamental differences between plants from different environments. A further limitation of the current implementation of SmT is that it does not yet achieve single-cell resolution. However, at least for the host, spot deconvolution allowed us to resolve the cell-type composition of spots.

We showcased SmT on *A. thaliana* leaves, which are an important model system for phyllosphere microbiology. We found microbial hotspots within plant leaves, reminiscent of microbial microniches in the human mouth^56,58^. An important question for future research will be whether there are specific leaf locations that favor a specific spatial organization of microorganisms within the leaf. We hypothesize that the invasion point at which the epiphytes entered the leaf is one factor governing the location of hotspots^59^, while the boundaries of hotspots may be set by the host response or simple ecological factors such as a local lack of nutrients in specific microenvironments^60^. An ecologically important aspect of microbial hotspots is that interactions are the strongest between microorganisms in close physical proximity^32,61^. New knowledge of interkingdom microbial interactions will be particularly valuable, given that interkingdom interactions can be associated with plant health^9,24^.

As for microbial interactions, studies of plant responses to microbial colonization have mainly been limited to analyses of homogenized whole tissues^26,27,62^. SmT now allows us to link microbial abundance at the micrometer level to host transcriptional responses. We found a high degree of overlap between the sets of genes associated with bacteria and fungi, implying a general response of leaf cells to microorganisms, although this generality could be driven by the extensive colocalization of bacteria and fungi in the sampled leaves. Furthermore, it may relate to the quantitative rather than qualitative difference in plant gene expression profiles to a diverse set of microorganisms^63^. Among the gene functions highly associated with microorganisms, chloroplast-related functions showed the greatest enrichment. This is consistent with reports linking chloroplasts to plant defense and pathogen invasion as well as photosynthesis^64^. This non-self-host-response profile we describe is less immune-centered than that recently described for the non-self *A. thaliana* response^63^. This difference is unsurprising given that (1) our study examined outdoor-grown plants instead of plants infected with individual microorganisms in a controlled environment, (2) we profiled host expression at a very late stage of the host–microbiota interaction (after a few months of growth) instead of just 9 d postinfection and (3) we describe the host response at the micrometer scale in different regions of individual leaves rather than the average response among homogenized leaves. Despite these methodological and conceptual differences, both studies revealed some similarities, such as the association between microbial infection and the immune-related gene *GSTF6* (AT1G02930), which was among the 24 general non-self-response genes that were discovered.

In conclusion, the versatility of SmT bodes well for its potential application to the many other tissue types ranging from plants to animals, including humans, where local differences in microbial colonization are an important determinant of health or disease.

## Methods

### Bacterial leaf-infiltration assay for microscopy

Seeds of *A. thaliana* (accession Col-0) were surface sterilized by an overnight incubation at −80 °C followed by washing with ethanol (5–15 min shaking in a solution of 75% EtOH (Sigma-Aldrich) and 0.5% Triton X-100 (Sigma-Aldrich), followed by a 95% EtOH wash and drying in a laminar flow hood). Stratification was done in a 0.1% agar solution at 4 °C for 7 d before planting. Seeds were sown on potting soil (CLT Topferde; www.einheitserde.de), in 60-pot trays (Herkuplast Kubern). During the first 2 d after sowing (the germination period), the trays were covered with a transparent lid to reduce the likelihood of pest infection. Indoor growing conditions were as follows: Cool White Deluxe fluorescent bulbs (25 to 175 μmol m^−2^ s^−1^), 23 °C and 65% relative humidity. Plants were grown under long-day conditions (16 h of light) for 15 d before syringe-infiltration with mCherry-tagged Pst DC3000 at OD_600_ = 0.001. Only half of the leaf was infiltrated (in relation to the main vein). A 3xmCherry construct had been inserted at the attn7 site and was a kind gift from Brian Kvitko.

Pst DC3000 was grown overnight in Luria Broth with the appropriate antibiotics (gentamicin and nitrofurantoin, 5 μg ml^−1^ each), then diluted 1:10 on the following morning, and was grown for an additional 4 h to initiate the log phase, after which the bacteria were centrifuged at 3500*g* for 90 s, and resuspended in 10 mM MgSO_4_.

Three days after infections, leaves were dissected and placed on 0.5× MS medium with agar (Duchefa, M0255), inspected under a Zeiss Axio Zoom.v16 fluorescence stereomicroscope to verify that the mCherry signal was present, and immediately flash-frozen in liquid N_2_. The leaves were stored at −80 °C before cryosectioning.

### Imaging of bacterial-infected leaves

Infected *A. thaliana* leaves were imaged on a Zeiss Axio Zoom.v16 fluorescence stereomicroscope, equipped with an LED array for transmitted illumination and an X-Cite XYLIS LED (Excelitas Technologies) for epi-illumination. All leaves were imaged using a PlanNeoFluar Z 1×/0.25 dry objective and a Hamamatsu ORCA-Flash4.0 digital CMOS camera (c11440-22C) with 2 × 2 binning. mCherry-tagged Pst DC3000 was detected using the Zeiss filter set 45 (00000-1114-462), which includes a 560/40 nm excitation filter, a 630/75 nm emission filter and a 585 nm dichroic mirror. Bright-field images were acquired as references for the outline of the leaves for the analysis. The camera exposure time was 220 ms at 5% of light intensity. The images of infected leaves have a pixel size of 18.6 µm^2^ and were acquired at a ×7 magnification. Image acquisition was done using the ZEN 2.1 software package.

### Outdoor-grown plants

For the analysis of microbial hotspots, microbial interactions and host responses to wild microbiomes, seeds of *A. thaliana* (accession Col-0) were germinated and grown indoors for seven short days (8 h of light). On 27 February 2019, the trays were placed outdoors near the Max Planck Institute for Biology Tübingen in a naturalized environment surrounded by other plants. Plants were irrigated weekly with regular tap water. Twenty-seven days after outdoor planting, individual leaves were sampled and immediately flash-frozen in liquid N_2_. Leaves from different plants were stored separately at −80 °C before cryosectioning.

### Axenically-grown plants

To grow axenic plants, *Arabidopsis* Col(0) seeds were pretreated at 37 °C for 24 h, followed by a cold treatment at −20 °C for 24 h. The seeds were then rinsed in 70% ethanol for 5 min, 20% chlorine for 20 min, and washed in sterile water three times before being transferred to Murashige Skoog plant agar plates. Subsequently, the seeds were vernalized at 4 °C for 48 h before allowing them to germinate and grow into seedlings, still on the MS plates, at 20 °C under long daylight conditions (16 h of light and 8 h of darkness). Ten days after the vernalization, individual leaves were sampled and immediately frozen in liquid N_2_. Leaves from different plants were stored separately at −80 °C before cryosectioning.

Two batches (‘1’ and ‘2’) of axenically-grown leaves were analyzed in the SmT experiments. Leaves were prepared as described in the subsection Sample preparation and sectioning. Three leaf sections from batch 1 and five leaf sections from batch 2 were cryosectioned and attached onto two multimodal array capture areas, respectively. In addition, from the same leaves, four sections per leaf were collected to a Lysing Matrix D tube (MP Biomedicals) for total RNA extraction, which was performed using the RNAqueous-Micro Total RNA Isolation kit (Invitrogen, Thermo Fisher Scientific) using minor modification. Specifically, the leaf sections were disrupted using a Fastprep-24 instrument (MP Biomedicals) in 50 μl of Lysis Buffer at 6.0 m s^−1^ for 40 s. Subsequently, the homogenized tissue lysate was centrifuged and transferred to the binding column followed by washes with wash buffers according to the manufacturer’s protocol. Finally, the total RNA was eluted in 20 μl of elution solution, and 10 μl from each of the two samples was added onto two multimodal array capture areas, respectively, during the cDNA synthesis (instead of tissue sections).

### Multimodal array structure

SmT uses multimodal slides (10x Genomics) with capture areas of 6.5 × 6.5 mm. Each capture area comprises 4,992 spots, with diameters of 55 μm each. The spots are covered with capture probes in the following proportion: 45% 16S rRNA probes, 45% 18S rRNA/ITS probes and 10% poly-d(T) probes.

### Probe design

Probes were designed using the following two approaches: one based on established primers of the relevant marker genes (*P799* (ref. ^65^) and *P902* (ref. ^66^)) and a de novo approach (*P1265* and *P479*) (Supplementary Fig. 18). On average, the probing sites were 100 nt upstream of the target site. In general, we aimed to maximize the following two variables: the conservation of the probe sites and the variability of the 100 nt downstream target sites. The de novo design process was adopted because previously designed primers were suboptimal with respect to these criteria.

Previously designed primers were used as templates due to their wide usage in the field, which is indicative of useful specificity—it implies that they have a wide taxonomic range and good ability to exclude host reads such as those originating from 16S chloroplast rRNA. Four probes were designed based on the following previous primers; the 16S probes 16S:P799 (5′-TTA VVG CRT GGA CWM CCM GGG TAT CTA ATC CKG TT-3′) and 16S:P902 (5′-CSS YTG TGY GSG GSC CCC CGT CAA TTC MTT TGA GTT TYA RYC-3′) were based on the mainstream primers 799F^65^ and 902R^66^, respectively. Additionally, the eukaryotic capture probes 18S:P-ITS1 (5′-CCT ACG GAA ACC TTG TTA CGA CTT TTT ACT TCC TCT AAA TGA CCA AG-3′) and ITS:P-ITS7 (5′-RRG CGC AAK RTG CGT TCA AAG ATT CGA TGA YTC AC-3′) were based on the mainstream primers ITS1F^67^ and ITS7F^68^, respectively. To fit the primers to the annealing conditions of the array, we reversed-complemented all forward-oriented primers (that is, all of them but 902R; the target RNA is single stranded, so reversal of the primer orientation was needed to capture it) and elongated them to obtain 35–45 bp long sequences, as recommended for microbial profiling in microarray systems^69^. To this end, 16S rRNA and ITS custom databases were downloaded (on 29 April 2020) from NCBI GenBank and the sequences downstream of the primer (up to 100 nt, including the primer) were extracted. These sequences were then aligned using the software Clustal Omega (v1.2.4) and the sequence profiles were plotted using weblogo (v3.7.5). The primers were elongated by manual inspection of the resulting weblogo. The length and degeneracy level were limited to obtain fewer than 35,000 unique probe sequences.

In addition to these probes, the following two de novo 16S probes were designed to complement the primer-based probes (as shown in Supplementary Fig. 18): 16S:P1265 (5′-GGT AAG GTT YYK CGC GTT GCD TCG AAT TAA ACC RCAT-3′) and 16S:P479 (5′-TCT CAG THC CAR TGT GGC YBD YCD YCC TCT CARR-3′). To design these probes, representative sequences were selected from the SILVA 16S database (v138.1) using CDHIT (v4.8.1) to the level of 99% sequence identity. Representative sequences were aligned using MAFFT (v7.245), and the sequence profile was plotted using weblogo (v3.7.5). In this process, we targeted highly variable regions with a conserved matching probing site.

### Sample preparation and sectioning

The leaves stored at −80 °C were immersed in 50% Optimal Cutting Compound (OCT, Sakura) in PBS (Medicago). Embedded samples were frozen in a cryostat (Cryostar NX70, Thermo Fisher Scientific) and sectioned to obtain 14-μm longitudinal sections. Tissue sections were then laid over the multimodal capture areas of the arrays.

### Tissue optimization experiment

Tissue permeabilization conditions were identified using a modified variant of a previously reported protocol^29^. Briefly, after attaching of the tissue section to the slide surface containing 100% poly-d(T) capture probes, the tissue was fixed in methanol (VWR) at −20 °C for 40 min and stained with 0.05% Toluidine Blue (Sigma-Aldrich) at room temperature for 2 min. Tissue sections were imaged using a Zeiss AxioImager 2T and a Metafer slide scanning system (v. 3.14.2, MetaSystems). They were then permeabilized with pepsin (Sigma-Aldrich) in 0.1% per 0.1 M HCl (Fluka) at 37 °C for 30 min. The plant mRNA molecules that had hybridized to the capture probes were reverse transcribed to cDNA using SuperScript III (Invitrogen, ThermoFisher Scientific) and Cy3-dCTP-nucleotides (PerkinElmer) at 42 °C overnight. Tissue sections were removed from the slide surface by incubation for 1 h at 37 °C in a hydrolytic enzyme mixture consisting of pectate lyase (Megazyme), xyloglucanase (Megazyme), xylanase 10A (Nzytech), β-mannanase 26A (Nzytech) and cellulase (Worthington) in monobasic sodium citrate (Sigma-Aldrich), pH 6.6. They were then incubated with 2% β-mercaptoethanol (Calbiochem) in RLT buffer (Qiagen) and proteinase K (Qiagen) in PKD buffer (Qiagen) for 1 h each. Finally, the fluorescent cDNA footprint was imaged using an Innoscan 910 (Innopsys) slide scanning system and Mapix image analysis software (v9.1.0, Innopsys) with a pixel size of 5.0 and a gain of 50.

### Sequencing library preparation

Sequencing libraries were prepared according to the Visium protocol (10x Genomics) with the following modifications: multimodal slides with leaf sections attached to the capture areas were incubated for 2 min at 37 °C followed by a 40-min fixation in methanol (VWR) at −20 °C. Capture areas were washed with PBS (Medicago) and incubated for 2 min at 37 °C. Tissue sections were stained for 2 min with 0.05% Toluidine Blue (Sigma-Aldrich) at room temperature followed by two washes with ultrapure water and warming at 37 °C for 2 min. The slides were mounted with 85% glycerol (Merck) and the bright-field images were acquired with a Zeiss AxioImager 2X microscope and a Metafer slide scanning system (v. 3.14.2, Metasystems) at ×20 magnification. To increase permeabilization efficiency and reduce the effect of secondary metabolites, the slides were incubated in 2% (wt/vol) polyvinylpyrrolidone 40 (PVP-40, Sigma-Aldrich) at room temperature for 10 min. Host plant and eukaryotic microbial cells were permeabilized using the permeabilization enzyme (10x Genomics) at 37 °C for 30 min. Bacterial organisms were permeabilized using 10 mg ml^−1^ lysozyme from chicken egg white (Sigma-Aldrich) in 0.05 M EDTA pH 8.0 (Invitrogen) and 0.1 M Tris–HCl, pH 7.0 (Invitrogen) for 30 min at 37 °C.

The rest of the SmT workflow followed the procedure described in the Visium Spatial Gene Expression user guide with the following modification: reverse transcription was performed using 2% (wt/vol) PVP-40 instead of nuclease-free water to reduce adverse impacts due to secondary metabolites and cDNA was amplified by performing 12–15 PCR cycles. Libraries were sequenced using the Illumina Nextseq 2000 and Nextseq 1000/2000 P2 or P3 Reagents (200 cycles) kit.

### Preprocessing of the reads and bright-field images

Template switch oligo and long poly-A stretches were removed from Read 2 using cutadapt v. 2.9 (ref. ^70^). The location of the tissue was determined using the Loupe Browser v. 5.1.0 (10x Genomics), in which all the spots containing at least 25% of the tissue were selected and their locations (that is, *x* and *y* coordinates) were recorded.

### Read alignment

TSO- and poly-A trimmed reads were analyzed using the ST Pipeline^71^ (v. 1.7.9, https://github.com/jfnavarro/st_pipeline), which enables simultaneous analysis of the spatial location, unique molecular identifier (UMI) and mRNA molecule. First, the pipeline trimmed poly-N stretches that are longer than 15 bp. Read 2 was then mapped against the *A. thaliana* TAIR10 genome release^72^ using the STAR v. 2.7.7a^73^ mapping tool and annotated with htseq-count 1.0 (ref. ^74^). The spatial barcode in read 1 was demultiplexed using Taggd (v. 0.3.6)^75^ and the information from read 1 and read 2 was combined. The ST Pipeline then grouped the reads based on the spatial barcode, gene and genomic location. Finally, the unique molecules were identified using a UMI and the counts were compiled into the gene-count matrix.

### Taxonomic assignment of microbial reads

Reads were mapped against the *A. thaliana* reference genome using STAR v. 2.7.7a^73^ and all reads aligning to the genome were discarded, leaving putative microbial reads. Next, read datasets were demultiplexed based on their probe types (that is, 16S rRNA and ITS/18S rRNA). For each probe dataset, the reads were first clustered into representative sequences by the fastx_uniques module of usearch v. 11.0.667 (ref. ^76^). Next, the representative sequences (query) were searched for the best homolog (hit) in the NCBI NT database (downloaded on January 2021)^77^ using MMseqs2 v. 1f30213 (refs. ^78,79^). For each query, all of the best hits (that is, those with the highest identical bit score and a taxonomic assignment on the genus level) were selected for further consideration. Next, the taxonomic assignment for a query was set as the lowest common ancestor (LCA) among the best hits as calculated by TaxonKit v. 0.7.2 (ref. ^80^) using the NCBI Taxonomy database (downloaded on January 2021)^81^. For 18S rRNA/ITS probes, reads were further considered if they were classified as Eukaryota but not as unclassified, chloroplast, mitochondria, uncultured, *Streptophyta*, *Chordata* or *Arthropoda* on the genus or the phylum levels. Similarly, for 16S rRNA probes, reads were considered if they were classified as bacteria but not as unclassified, chloroplast, mitochondria or uncultured on the genus level. Finally, reads were further filtered by their UMI, such that for each spatial location, only one representative read with a given UMI was retained. The number of reads considered for each dataset is provided in Supplementary Table 11.

The annotation of the sequences used for taxonomic assignment was assessed to confirm that they originated from the expected locus. On average, 93.7 and 96.8% of the sequences captured by the 16S and ITS probes, respectively, were annotated as 16S and ITS rDNA loci, and most of the rest were annotated as full genomes, which include 16S and ITS rDNA loci (Supplementary Table 12). We further validated the observed microbial profiles by confirming that the reads containing each of the targeted probes fell within the expected range when aligned against the corresponding sequences in the NCBI ‘nt’ database (Supplementary Fig. 47). This further confirms that the reads originated from the expected targeted region.

### Pst DC3000 infection experiment—data processing

Processed, aligned reads were analyzed using STUtility (v. 0.1.0)^82^. To exclude low-quality spots, the *A. thaliana* host data and bacterial-unique molecules were summed together and every spot with fewer than 20 unique molecules was discarded. Each spot containing less than 10 unique genes/taxa was discarded. The visualized genes and taxa were log_10_ normalized and projected on a bright-field image of the tissue section with an opacity of 0.75.

The maximum fluorescence intensities for each spot location were performed by manually aligning the fluorescence image and bright-field image. Then Matlab (2022a) was used to identify the centers of the spots and the *k*-nearest-neighbor algorithm was implemented to identify pixels that are a maximum of 27.5 μm away from the center. Maximum fluorescence values for each of the spots were extracted.

To generate the scatter plots and Pearson correlation, log_10_-normalized SmT captured unique *Pseudomonas* molecules were plotted against the log_10_-normalized host *PR1* gene expression and the log_10_-normalized maximum *Pseudomonas* fluorescence values from the fluorescence imaging using ggplot2 (v. 3.3.5.)^83^. The Eulerr^84^ package in R was used to generate the Venn-diagrams with a cutoff of 45 and 120 for leaves 1 and 2, respectively, to remove the background fluorescence signal and minimum of 1 unique molecule per spot for SmT captured *Pseudomonas* and *PR1*. Hotspots analysis using the fluorescence signal values with the applied cutoffs, as well as using the SmT reads, was performed as described in the subsection Analysis of microbial hotspots.

### Enrichment experiment

Glass slides bearing a multimodal capture array (10% poly-d(T) probes, 45% bacterial 16S rRNA probes and 45% eukaryotic 18S rRNA/ITS probes), a 100% poly-d(T) array, a 100% bacterial 16S rRNA array and a 100% eukaryotic 18S rRNA/ITS array were used. Three leaves were sectioned on each of these capture slides meaning every leaf had a consecutive section on each array type. Sequencing libraries were prepared as per the above protocol and sequenced with Nextseq 2000 (Illumina). The reads were annotated as described above and analyzed using R (v. 4.0.5).

STUtility (v. 0.1.0) was used to read the *A. thaliana* data to an object and sums of gene values were log_10_-transformed. Pairwise Pearson correlation coefficients were calculated and visualized with the corrplot package (v. 0.92) function corrplot.mixed using significance levels of 0.001, 0.01 and 0.05, with hierarchical clustering permitted. The scatter plots are visualized using ggplot2 (v. 3.3.5.)^83^.

For bacterial 16S rRNA and eukaryotic 18S rRNA/ITS data, unique molecules were summed together per taxon, generating a table containing the sum of unique molecules, phylogenetic paths and metadata relating to section identification. Any annotations to phylum *Streptophyta* were removed, after which the data were divided into bacterial and fungal datasets based on their superkingdom. For taxonomic rank plots, the unique molecules for the different taxonomic levels were counted and compared with the 100% poly-d(T) array to calculate the fold change for microbial taxa at each of the taxonomic levels. Pairwise correlations, and unique molecules for each taxonomic rank, were only calculated for classified reads. We performed the analysis three times—first with all taxa and then with only the most highly expressed 500 and 20 taxa. Shannon diversity and Bray–Curtis similarity were calculated using vegan R package (v. 2.5-7)^85^.

#### Simulation of probe concentration and effect on diversity

Different proportions of reads—ranging from 5 to 95%—were sampled of samples analyzed on a 100% 16S rRNA or 18S/ITS rRNA array to simulate the effect of different probe concentration on the captured microbial diversity (Shannon diversity index). The procedure was repeated 100 times. The distribution of this simulated Shannon diversity is presented together with the diversity observed in the 45% probe concentration multimodal SmT array.

Saturation of the host information was calculated by subsampling the annotated reads to the saturation point (2,000; 3,718; 8,389; 21,085; 55,598; 149,413; 404,428; 1,097,633 and 2,981,957 reads), and unique molecules and genes were counted and plotted against the saturation points.

### Validation of SmT with amplicon sequencing

To compare the performance of SmT to that achieved with amplicon sequencing, seeds of *A. thaliana* (accession Est-1) were surface sterilized and stratified at 4 °C for 1 week in a refrigerator, and then sown in plastic trays (Herkuplast) filled with wild soil from the Heuberger Tor experimental site of the University of Tübingen (Germany). The seeds were left outside to germinate in the same field in late September. The plants developed and overwintered without supplemental watering. Additional plants in each pot were thinned in January 2020 with tweezers, and individual plants were sampled at the end of March 2020 before flowering. The sampling protocol involved cutting the mature rosettes with sterile scissors, placing them in sterile 50 ml centrifuge tubes, and vigorously shaking them in sterile water. The water was then dumped and new water was added until the leaves released no further dirt. After washing, plants were immediately flash-frozen in liquid N_2_, and subsequently stored at −80 °C prior to nucleic acid extraction. Both DNA and RNA were extracted from each plant. The entire rosette was lysed in a buffer containing 2% β-mercaptoethanol to extract all nucleic acids while preserving RNA. One proportion of the lysate was used for RNA extraction by the phenol/chloroform protocol, while another portion was used to extract DNA following a previously described potassium acetate and SPRI bead protocol^86^. The DNA moiety was used for 16S rDNA amplicon sequencing. The following two sets of primers were used: (1) 515F-806R (V3-V4) in combination with plastid-blocking clamps^87^ and (2) 799F-1192R (V4-V6), which does not amplify chloroplasts and for which the mitochondrial amplicon was removed by gel extraction^88^. The RNA moiety was used for SmT, using the same pipeline as for all other samples with the exception that crude extracts were used in place of leaf samples (so spatial information was not extracted). A total of 300 μg of RNA was used for the array. In total, four plant samples were used for 16S rRNA profiling, comparing two amplicon sequencing primer sets to the SmT array, with the exception of leaf C for which amp-seq 799F-1192R was not performed. The reads obtained by amplicon sequencing were analyzed in the same way as the array reads, excluding the initial mapping to the *A. thaliana* TAIR10 database. For both of the methods, the reads were subsampled to the same sequencing depth. See the ‘Read alignment’ and ‘Taxonomic assignment of microbial reads’ subsections for information about the full pipeline.

Spearman correlation was calculated between all taxonomic profiles at the genus level (each amp-seq-primer-pair-profile with the SmT-profile and with the other primer-pair derived profile). In all comparisons, only taxa that were detected in both profiles were accounted for. The analysis was performed and plotted using the ggpairs function which is part of R GGally package (version 2.1.0)^89^.

### Analysis of microbial hotspots

Microbial hotspots (based on 16S rRNA/ITS reads) were identified using the Getis-Ord G statistic^90^ as implemented in the localG function of the R spdep package (v. 1.1.11)^91^. The calculation was performed using a 2 × 2 grid applied to the count matrix resulting from the sum of reads belonging to the 50 most abundant genera (separately for 16S rRNA/ITS reads). A similar calculation was done for individual host genes so that the association between microbial and G-values for individual host genes could be done. The p.adjustSP function of the R spdep package was used with the BH-FDR^92^ method to correct the G stats *P* values while accounting for the number of neighbors of each region. Hotspot spatial maps were plotted using the R tmap package (v. 3.3-2).

### Microbial interaction network analysis

Microbial interactions were inferred based on the Spearman rank correlation coefficient (SRCC) values of the reads count associated with each pair of genera. Specifically, for each pair of microbial genera, in each leaf section, SRCC was calculated accounting for all spots of the array (that is, each spot on the array was considered as a ‘sample’ for each genus). We considered pairs of genera to be interacting if their SRCC-corrected *P* value (BH-FDR) was below 0.05. Next, to account also for the spatial organization of microorganisms in the array, we computed the SRCC value of each candidate pair based on shuffled abundance matrices. This step, repeated 1,000 times, results in an empirical null distribution of expected SRCC values where the spatial association between paired genera is random. The shuffled count matrix was generated by using the permatfull function implemented in the R vegan package (v. 2.5.6) while keeping the total number of reads associated with each genus across all samples (spots) constant (that is argument fixedmar = ‘columns’). Finally, the significance of each candidate pair of genera was calculated by comparing the SRCC value based on the unshuffled count matrix to the empirical null pair distribution^93^ following a BH-FDR correction. Microbial interactions were considered to be also spatially significant if their corrected-empirical *P* value was below 0.05. The network was created based on these microbial pairwise correlation values using the R igraph package (v. 1.2.6) and plotted using the R ggraph package (v. 2.0.5).

### Host mRNA clustering

For the *A. thaliana* host data, the counts were filtered using STUtility (v. 0.1.0)^82^ by removing the low-quality spots and genes containing at least 10 and 30 counts, respectively. In addition, each spot was required to have at least 10 genes and each gene was required to cover at least 20 spots. Chloroplast, mitochondrial, ribosomal and noncoding genes were filtered from the data set because many of them are not polyadenylated and might contain genes captured with 16S rRNA and 18S rRNA/ITS probes. Finally, after the filtering steps, the spots with fewer than 10 genes were removed because they were considered to be of low quality.

Each section was normalized individually using the Seurat (v. 4.1.0)^94^ function sctransform^95^ to eliminate intrasection batch effects. To reintegrate the sections back together, anchor features were selected and the whole data was scaled based on these features. Principal component analysis (PCA) was performed on this data using identified variable features. Based on the results of the PCA, the intersection batch effects (experiment date, plant and leaf) were removed with Harmony (v. 0.1.0)^96^ using a diversity clustering penalty of 4 and PCA dimensions of 1 to 8.

Normalized gene counts were projected onto 2D leaf section images using UMAP^33^ with the eight first dimensions from Harmony and a resolution of 0.22. To identify cluster-specific markers, raw counts were normalized using the NormalizeData function with LogNormalize as a normalization method and the FindAllMarkers function with the parameters of test.use = ‘poisson’ and logfc.threshold = 0.15.

### Spot cell-type deconvolution

Cell-type proportions in the spatial host data were analyzed using Stereoscope (v. 0.3)^97^ with the Single Cell Leaf Atlas data^98^, who kindly provided the raw count data and cell-specific annotations. Stereoscope used raw gene-count matrices from single-cell data and raw spatial data from which spots outside the tissues had been removed. The stereoscope was run with a –gpu setting using batch sizes of 2,048 and epoch sizes of 50,000 for spatial and single-cell dataset and 5,000 most highly expressed genes from the single-cell dataset.

The single-cell data contained 19 clusters, which were reduced to the following five: mesophyll (11 clusters), vascular (4 clusters), epidermis (1 cluster), guard cell (1 cluster) and hydathode (1 cluster). These collapsed as well as the 19 original clusters were projected on tissue using STUtility (v. 0.1.0)^82^ and heatmaps for each of the clusters were generated with pheatmap (v. 1.0.12)^99^. To aid the visual interpretation, the cell-type proportions were scaled by quantiles using the 95th percentile of the data in each section and cell type.

### Host-response analyses

We used the Boruta algorithm^30^ to determine which set of *A. thaliana* genes is important to explain the microbial load on each spot of the array. Briefly, we modeled the relationship between the expression profile of all *A. thaliana* genes—*G*_*1*_*…G*_*n*_ and *M*—the sum of the 50 most abundant bacterial/fungal genera in each spot of the array (*M*)—*M* ~ *G*_1_…*G*_*n*_. We treated the task as a regression problem and used the random forest algorithm^100^ to calculate the importance of each gene in the model. Next, we used Boruta to assign a significance score for each gene based on its importance for the model’s accuracy. For this purpose, we used the R implementation of the Boruta package (v. 7.0.0) with 1,000 trees. This procedure was performed for each leaf section, once using the un-normalized read counts and once using the Getis-Ord G statistic value, treating each spot as an observation. Overall, a gene was considered further if it was found to be significant by Boruta for at least one measure (that is, reads count or G statistics), and if its SRCC *P* value (after FDR correction) was below 0.01. GO enrichment analyses were performed with the DAVID web server with the DAVID knowledgebase v2022q1 (refs. ^101,102^).

### Reporting summary

Further information on research design is available in the Nature Portfolio Reporting Summary linked to this article.

## Online content

Any methods, additional references, Nature Portfolio reporting summaries, source data, extended data, supplementary information, acknowledgements, peer review information; details of author contributions and competing interests; and statements of data and code availability are available at 10.1038/s41587-023-01979-2.

## Supplementary information

Supplementary Information Supplementary Figs. 1–47.

Reporting Summary

Supplementary Tables Supplementary Tables 1–12.

## Data Availability

Sequencing data have been deposited at NCBI-SRA under the BioProject PRJNA784452. Source data files (bright-field images, alignment matrices, putative microbial reads and annotation files and gene/taxa matrices) for each of the experiments have been deposited to Zenodo (10.5281/zenodo.8308137)^103^.

## Code availability

Scripts written for the analyses described in this paper are available on GitHub (https://github.com/giacomellolab/SpatialMetaTranscriptomics)^104^.
