# Supplementary material for: Spatial metatranscriptomics resolves host–bacteria–fungi interactomes
Source: Nat Biotechnol. 2023 Nov 20;42(9):1384–93. doi: 10.1038/s41587-023-01979-2 (PMC11392817; doi:10.1038/s41587-023-01979-2)
Supplement: Supplementary file 1 — Supplementary Figs. 1–47. [file 41587_2023_1979_MOESM1_ESM.pdf]

nature biotechnology

Article https://doi.org/10.1038/s41587-023-01979-2

Spatial metatranscriptomics resolves host–bacteria–fungi interactomes

In the format provided by the authors and unedited

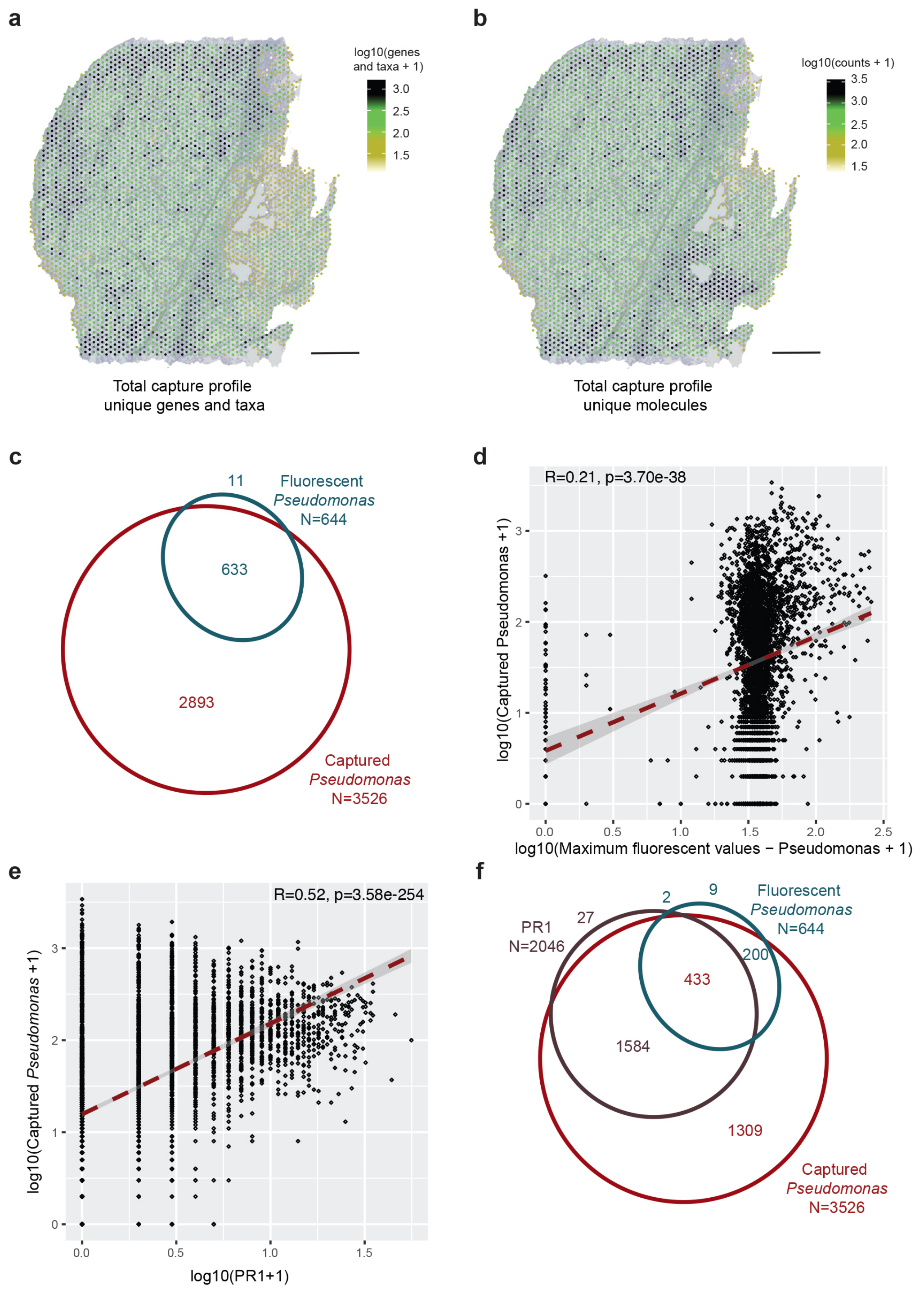

Supplementary Fig. 1: Combined capture profiles of host unique genes and microbial taxa (**a**), and of unique molecules of the host and the microbes (**b**) across the tissue section from *Pseudomonas* infected leaf 1. Scale bar is 1 mm. **c**, Overlap of the spots containing SmT-captured *Pseudomonas* and fluorescence imaging captured *Pseudomonas.* **d**, Scatter plot and the corresponding Pearson correlation (R=0.21, p=3.70e-38, two-tailed test) of log normalised *Pseudomonas* signal captured with the SmT array and the fluorescent imaging (maximum fluorescence intensity per spot area). The red line shows the fitted linear model with 0.95 confidence interval. **e**, Scatter plot and the corresponding Pearson correlation (R=0.52, p=3.58e-254, two-tailed test) of log normalised *Pseudomonas* and *PR1* host gene captured by the SmT array. The red line shows the fitted linear model with 0.95 confidence interval. **f**, Overlap of the spots containing SmT-captured *Pseudomonas*, fluorescence imaging captured *Pseudomonas* and host *PR1* gene. Data presented here is for leaf 1.

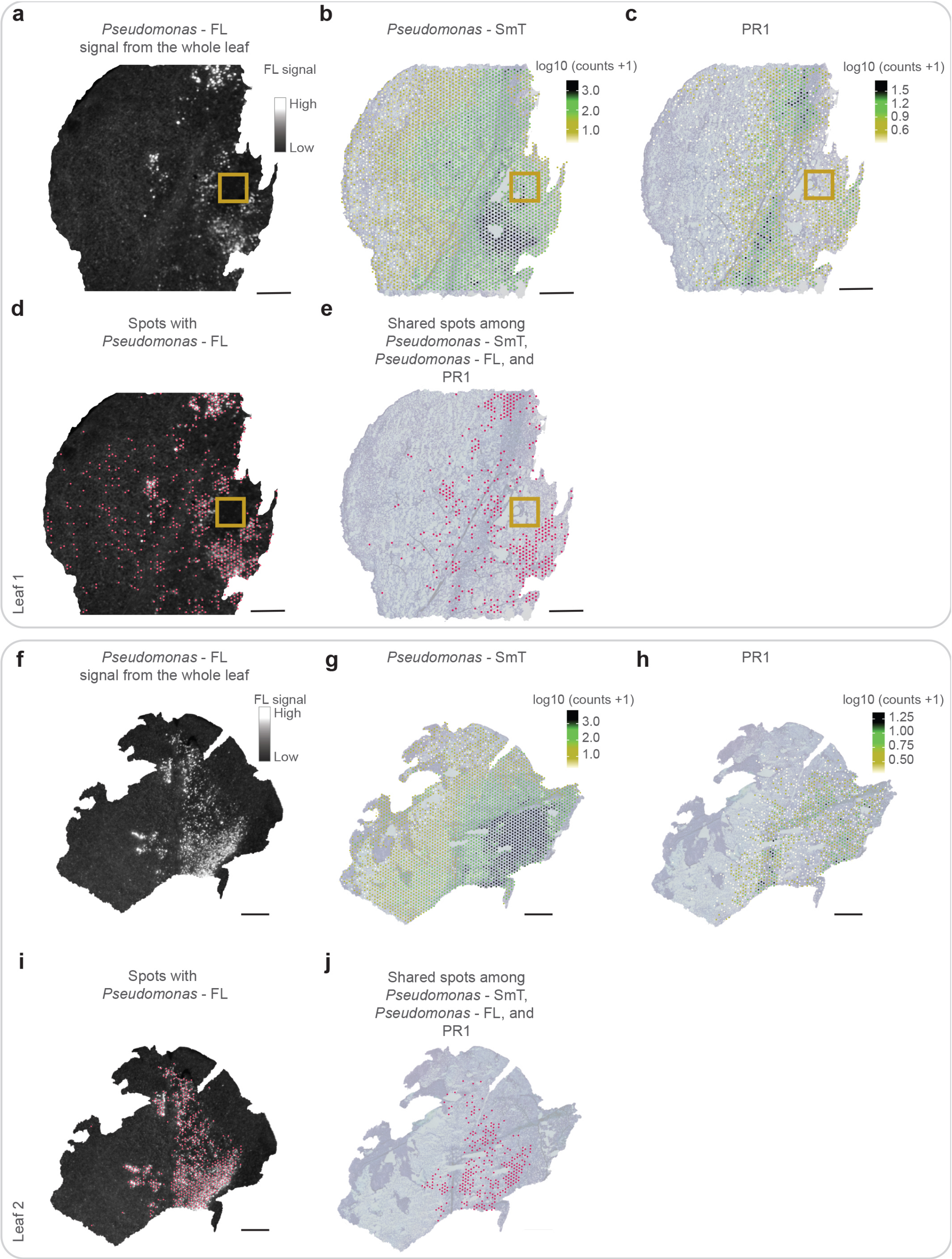

Supplementary Fig. 2: Analysis of two *Arabidopsis* leaves infected with fluorescently labelled *Pseudomonas* DC3000. We used fluorescent microscopy to visualize the signal from *mCherry*-labelled *Pseudomonas* DC3000 bacteria from whole intact leaves (a and f). Afterwards, we sectioned the leaves and profiled separate sections using SmT identifying a higher abundance of *Pseudomonas* DC3000 bacteria in correspondence of the higher fluorescent *Pseudomonas* signal (**b** and **g**) and expression of the host immune gene *PR1* (**c** and **h**). We identified all the spots containing the fluorescent signal (**d** and **i,** see **Methods**) and visualized the spots containing SmT and fluorescence microscopy-derived *Pseudomonas* DC3000 signal and the host *PR1* immune gene (**e** and **j**). Scale bar is 1 mm.

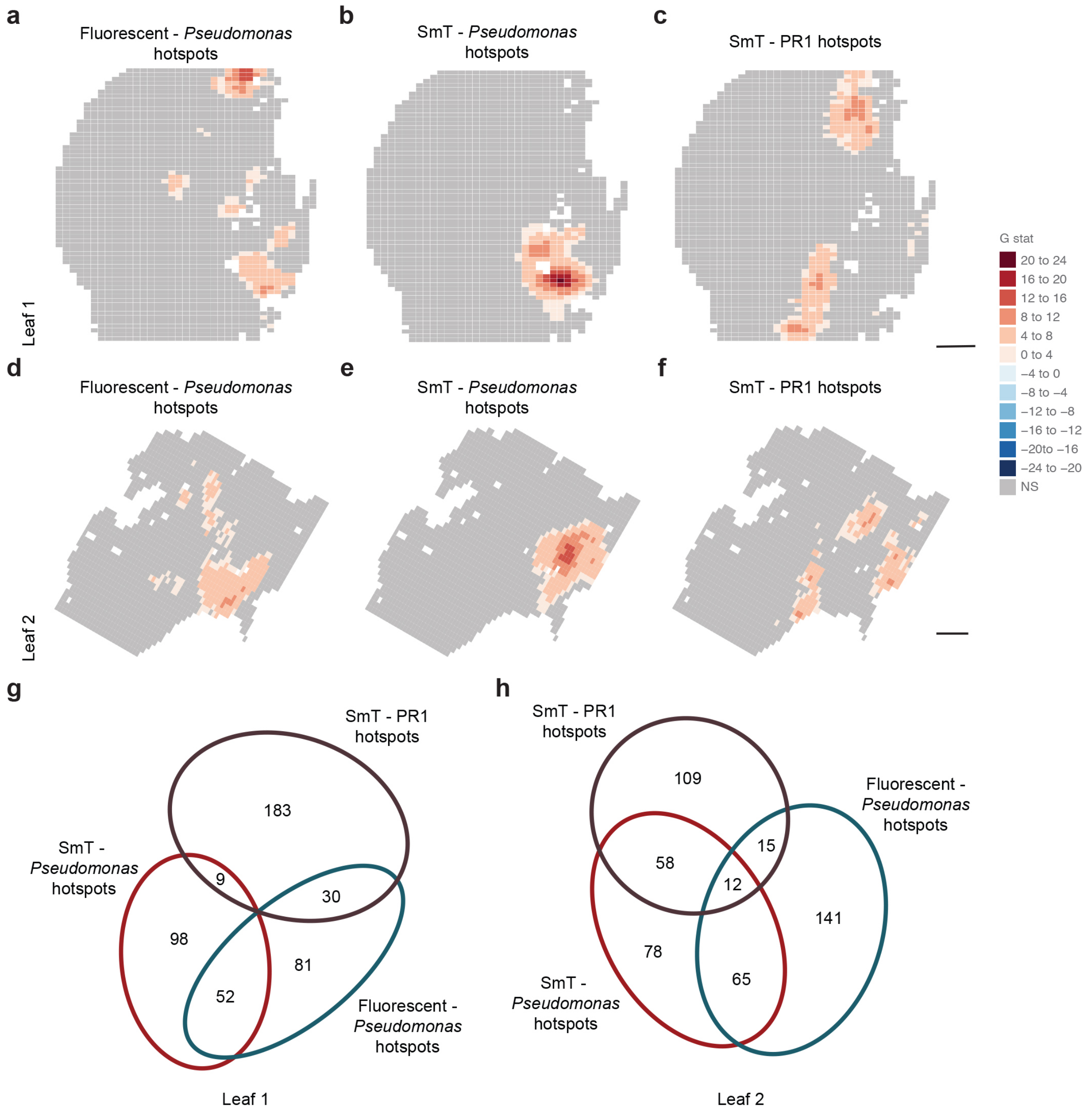

Supplementary Fig. 3: Significant hot- and cold-spots of *Pseudomonas* DC3000 as derived from the fluorescent signal (**a** and **d**) and SmT signal (**b** and **g**), and Significant (BH-FDR corrected p-value≲ 0.05) hot- and cold-spots of the expression of the gene *PR1* (**c** and **f**). Results are presented for two separate leaves (**a**-**c** and **d**-**f**). Overlap between significant hotspots is presented in panels **g** and **h**. For the fluorescent signals, only values above 45 and 120 were considered for leaf 1 and leaf 2, respectively. Scale bar is 1 mm.

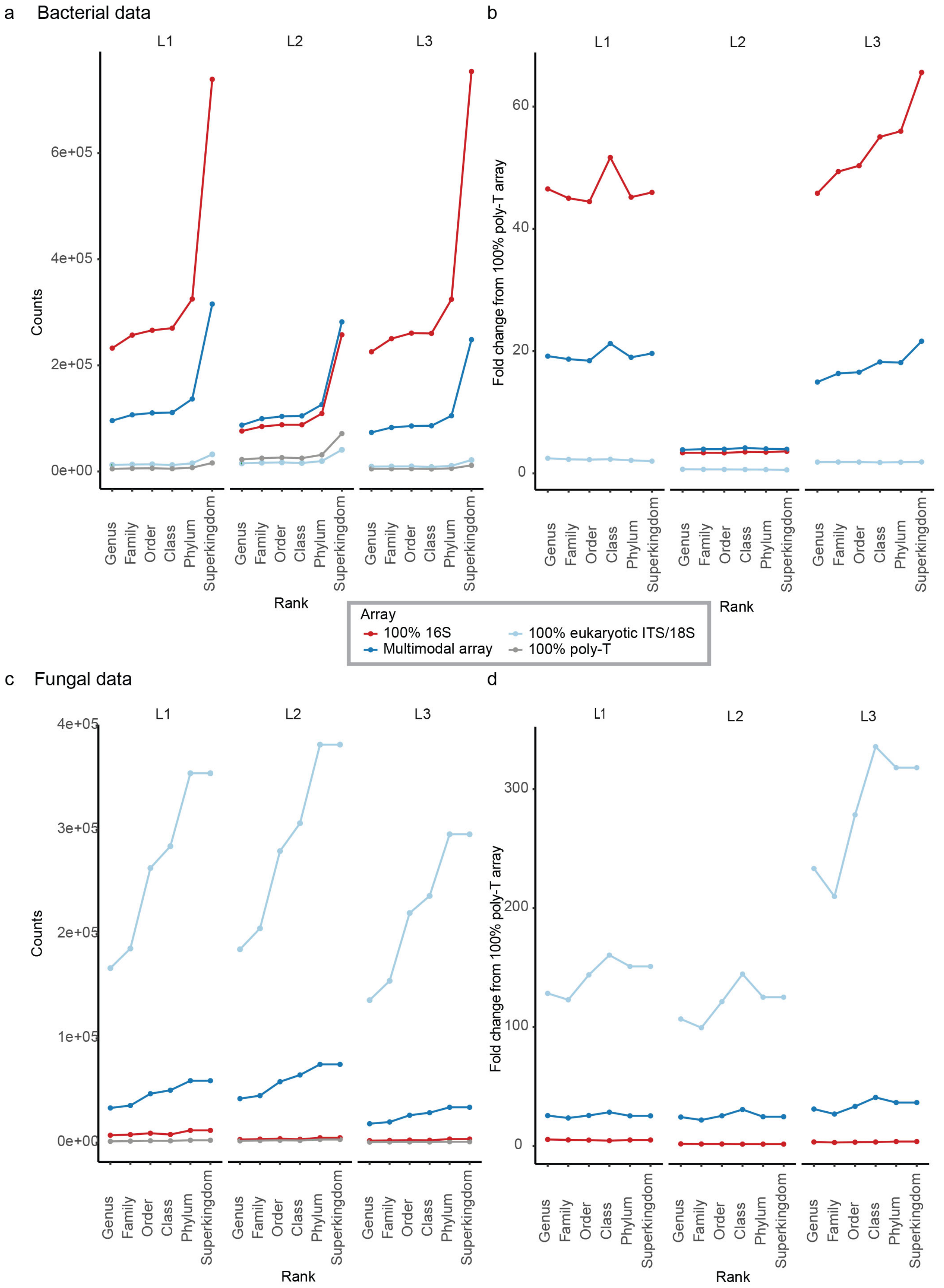

Supplementary Fig. 4: a, Number of unique molecules presented at each taxonomic level for bacterial data in each array and **b**, the fold change between different taxonomic levels from unspecific binding where an array of 100% poly-d(T) probes has been used as a baseline. Fungal unique molecules and the fold change as a function of taxonomic level are presented in **c** and **d**, respectively. L1, L2 and L3 stands for Leaf 1, Leaf 2 and Leaf 3, respectively.

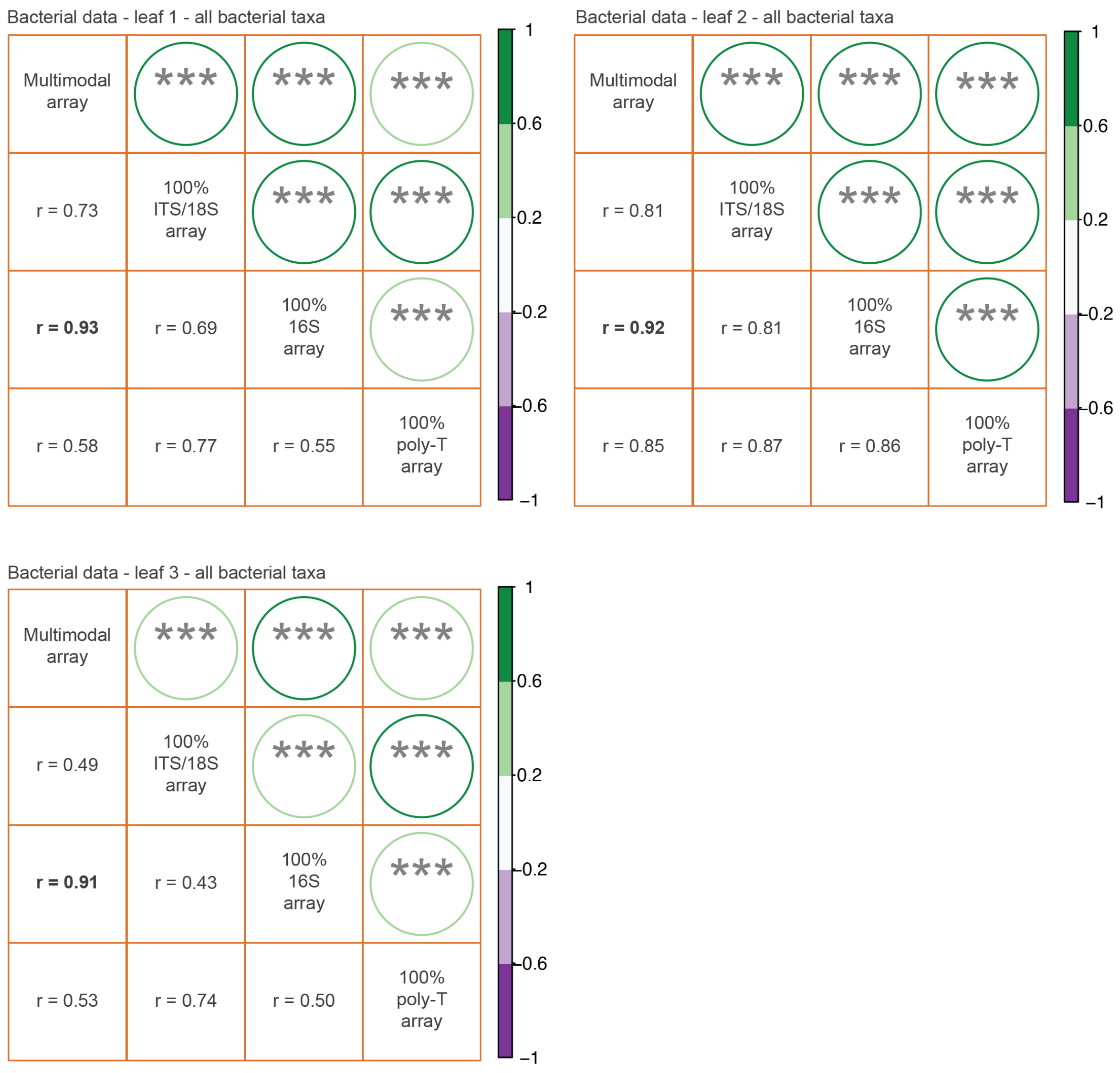

Supplementary Fig. 5: Pairwise Pearson correlation coefficients and the corresponding two-tailed significance test of bacterial 16S components between different array types in three leaves when the full bacterial profile with 1681 taxa is considered. *** indicates the p-value=0. Correlation between the multimodal array and the 100% 16S array is in bold.

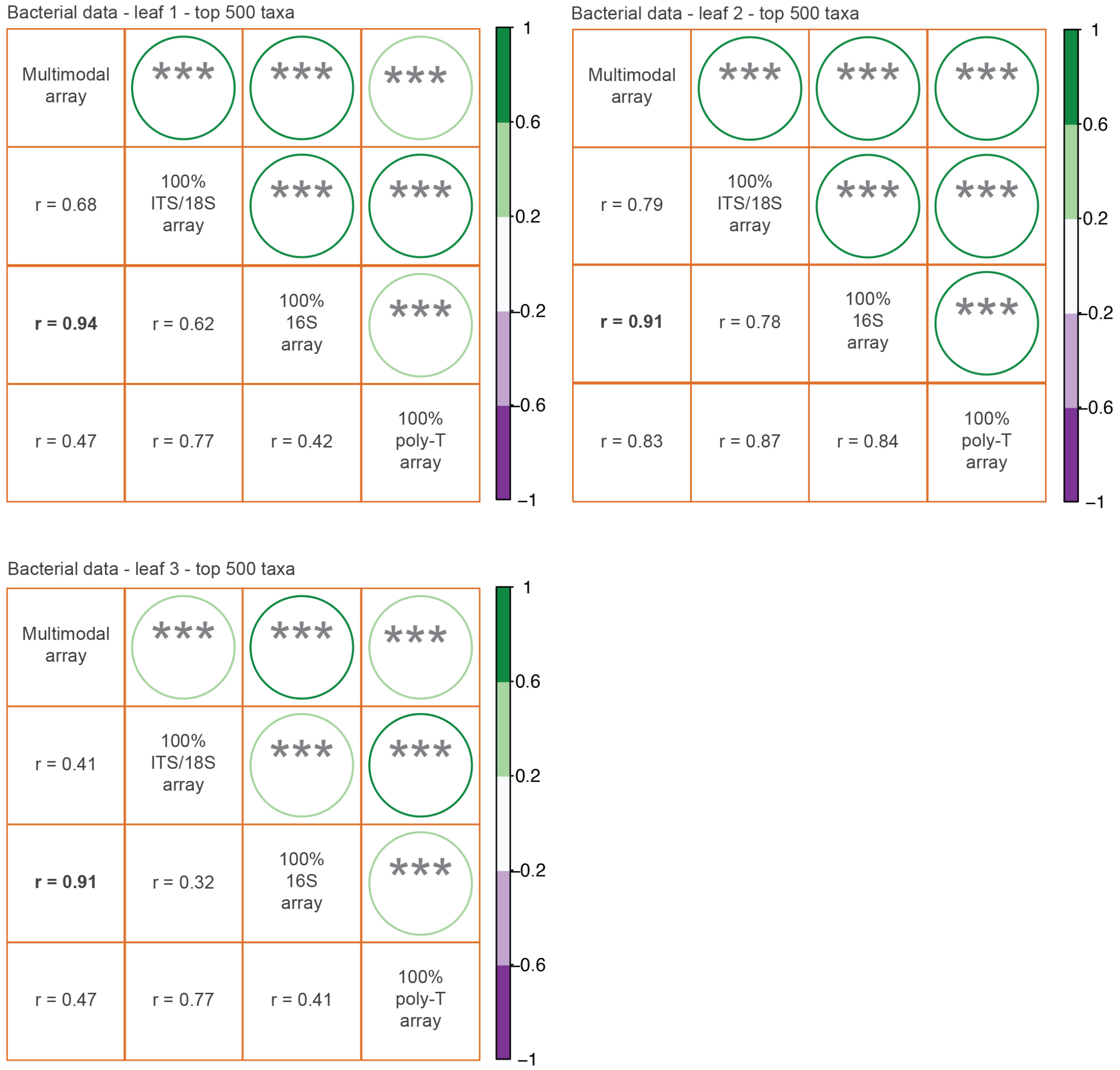

Supplementary Fig. 6: Pairwise Pearson correlation coefficients and the corresponding two-tailed significance test of bacterial 16S components between different array types in three leaves when the 500 most abundant bacterial taxa are considered. *** indicates the p-value=0. Correlation between the multimodal array and the 100% 16S array is in bold.

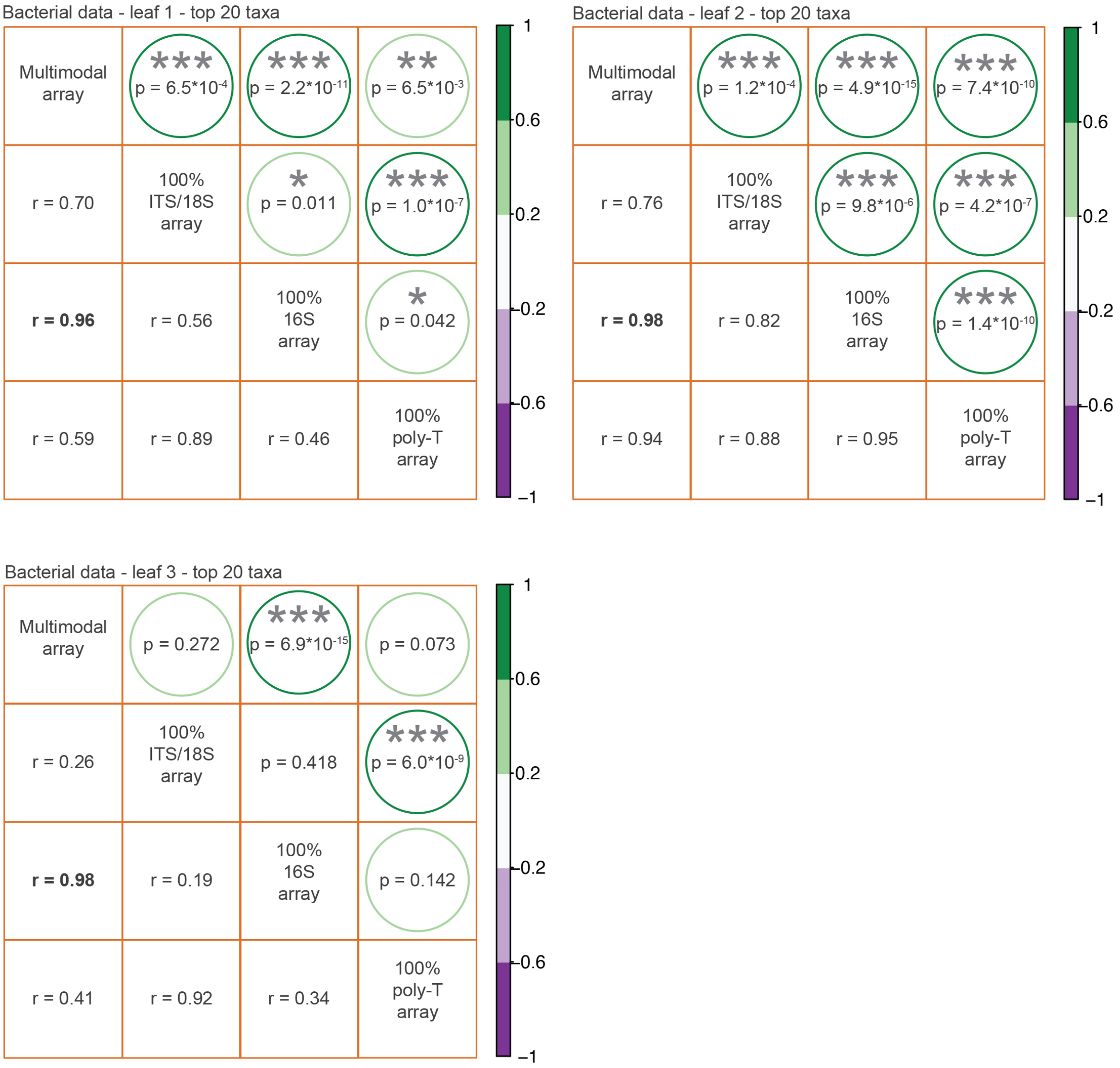

Supplementary Fig. 7: Pairwise Pearson correlation coefficients and the corresponding two-tailed significance test of bacterial 16S components between different array types in three leaves when the 20 most abundant bacterial taxa are considered. *: P<0.05, **: P<0.01, ***: P<0.001. Correlation between the multimodal array and the 100% 16S array is in bold.

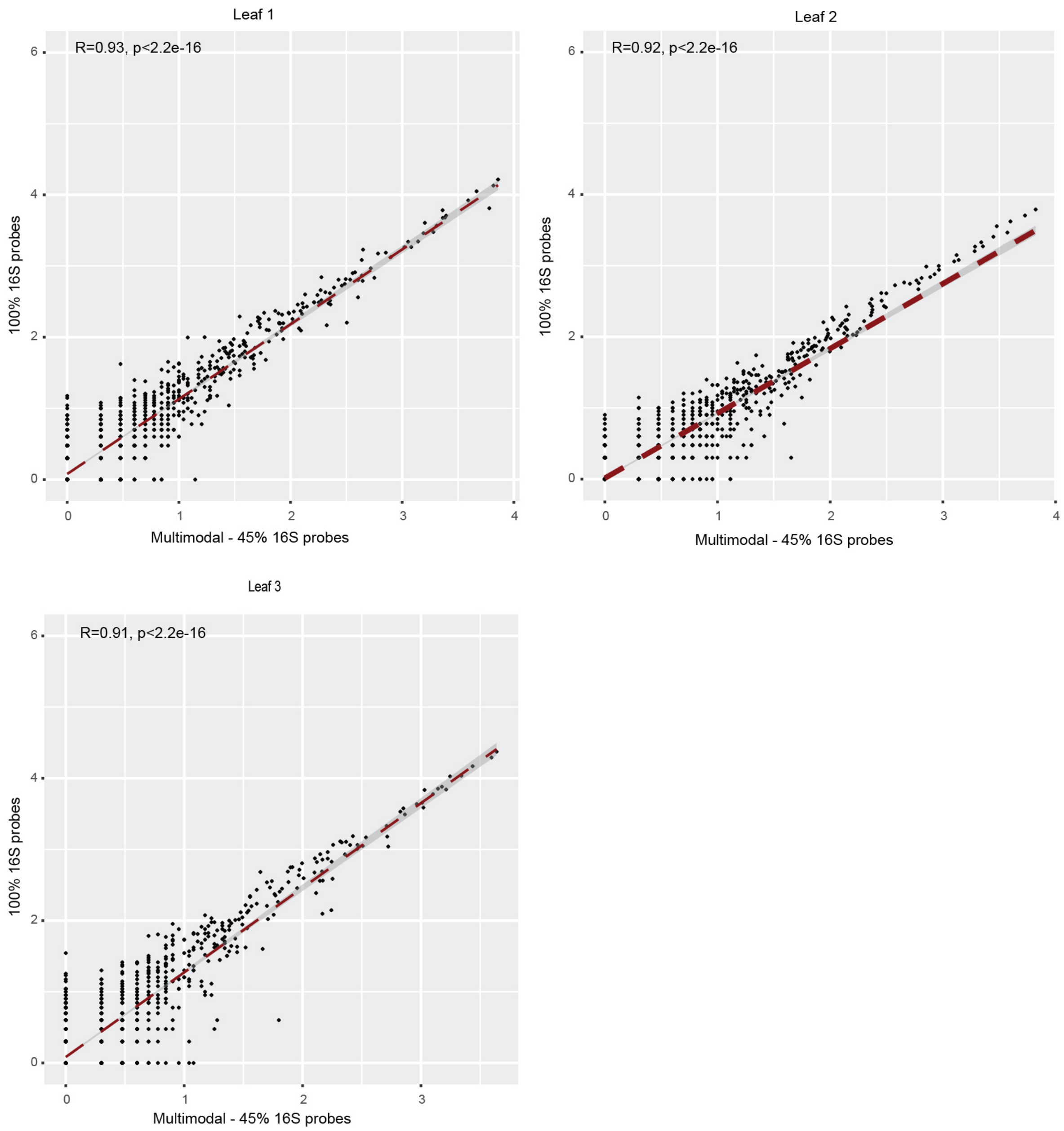

Supplementary Fig. 8. Scatter plots of log normalised unique bacterial molecules and the corresponding Pearson correlation coefficient and the corresponding two-tailed significance test for arrays with 100% and 45% bacterial 16S rRNA probes in leaves 1 to 3. For leaves 1, 2, and 3 the Pearson correlation values are 0.93, 0.92, and 0.91 ($p<2.2e-16$), respectively. The red line shows the fitted linear model with 0.95 confidence interval.

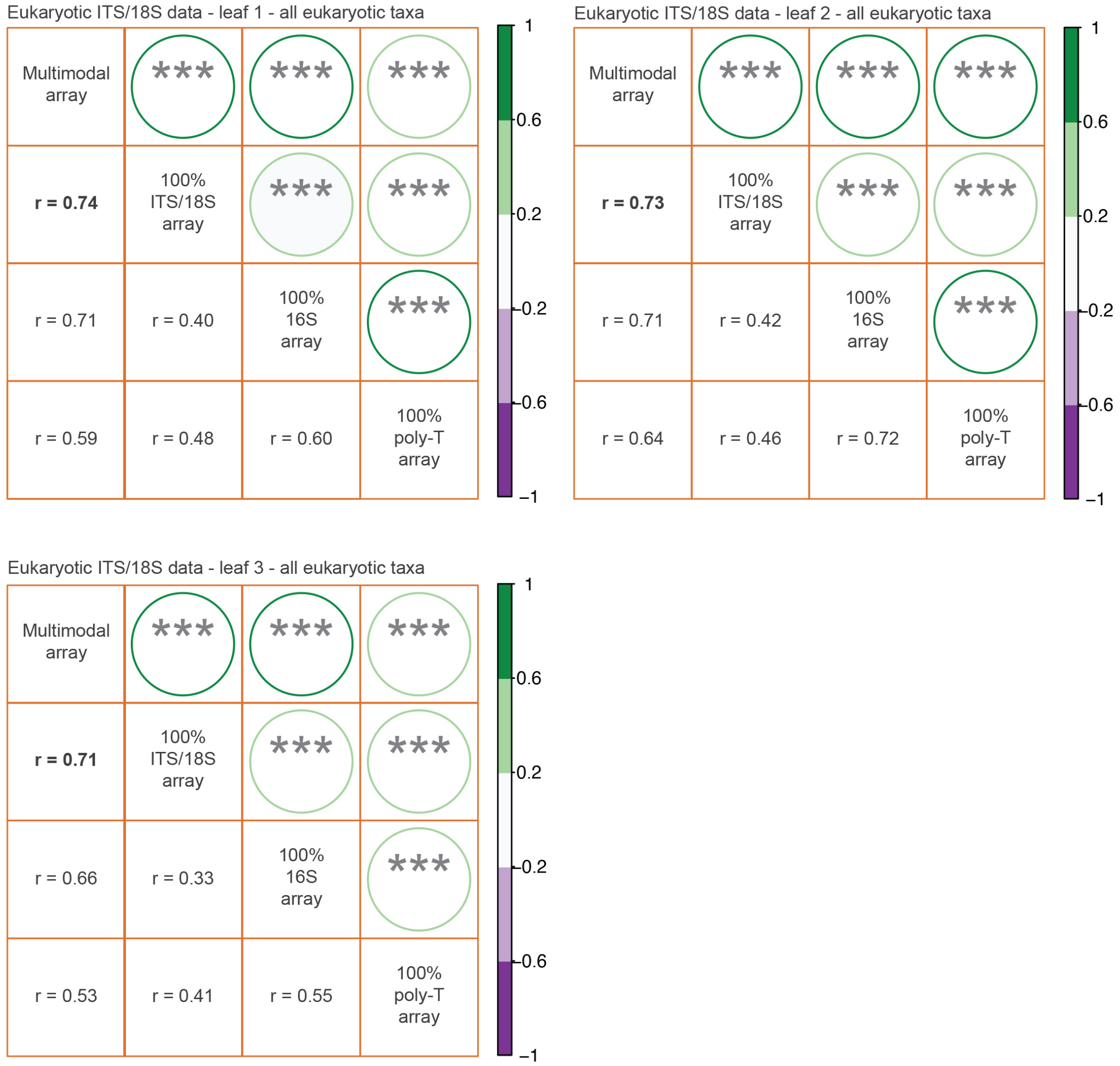

Supplementary Fig. 9: Pairwise Pearson correlation coefficients and the corresponding two-tailed significance test of eukaryotic ITS/18S components between different array types in three leaves when the full eukaryotic profile with 1660 taxa is considered. *** indicates the p-value=0. Correlation between the multimodal array and the 100 % Eukaryotic ITS/18S array is in bold.

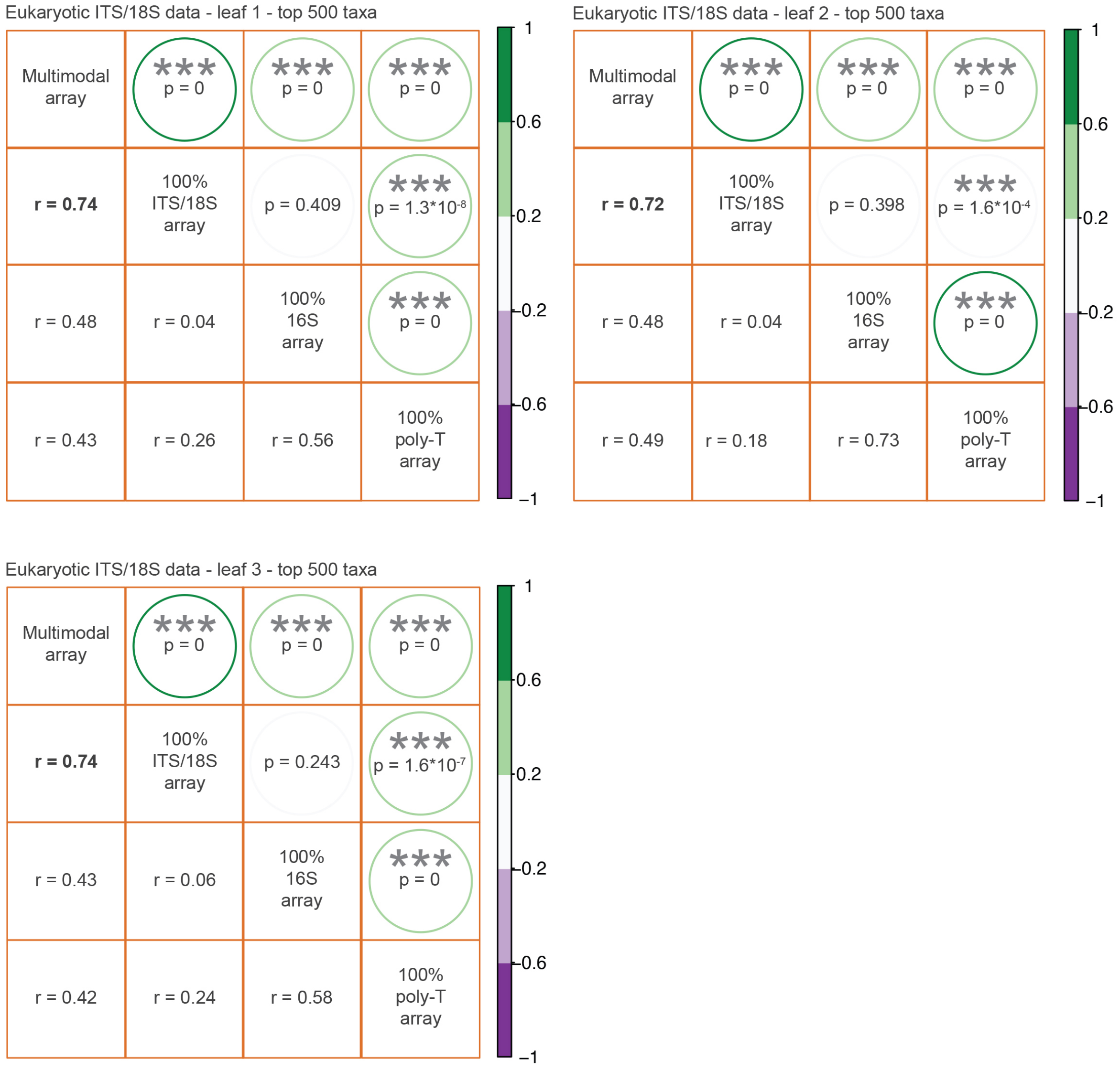

Supplementary Fig. 10: Pairwise Pearson correlation coefficients and the corresponding two-tailed significance test of eukaryotic ITS/18S components between different array types in three leaves when the 500 most abundant eukaryotic taxa are considered. *: P<0.05, **: P<0.01, ***: P<0.001. Correlation between the multimodal array and the 100% Eukaryotic ITS/18S array is in bold.

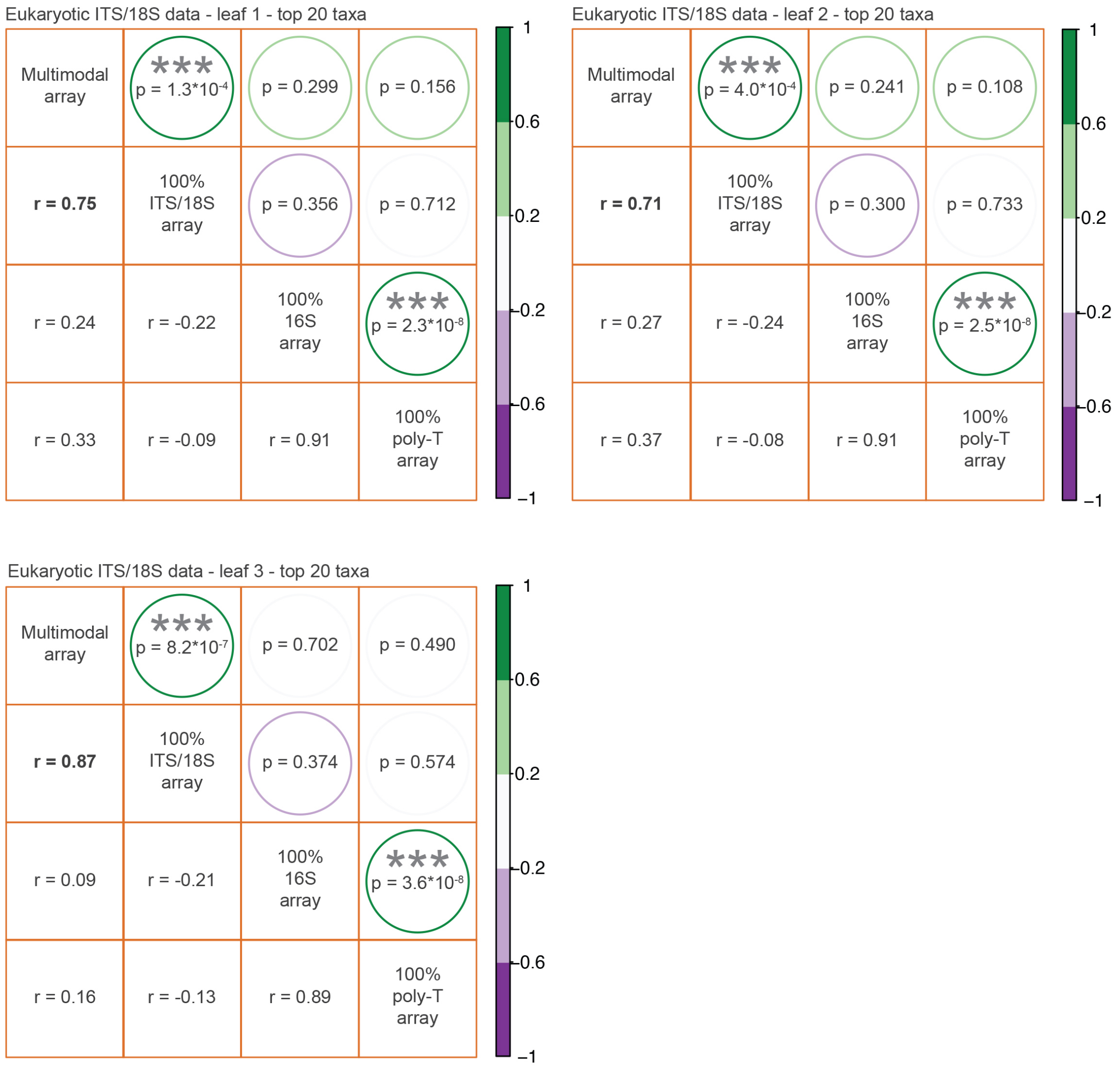

Supplementary Fig. 11: Pairwise Pearson correlation coefficients and the corresponding two-tailed significance test of eukaryotic ITS/18S component between different array types in three leaves when the 20 most abundant eukaryotic taxa are considered. *: P<0.05, **: P<0.01, ***: P<0.001. Correlation between the multimodal array and the 100% Eukaryotic ITS/18S array is in bold.

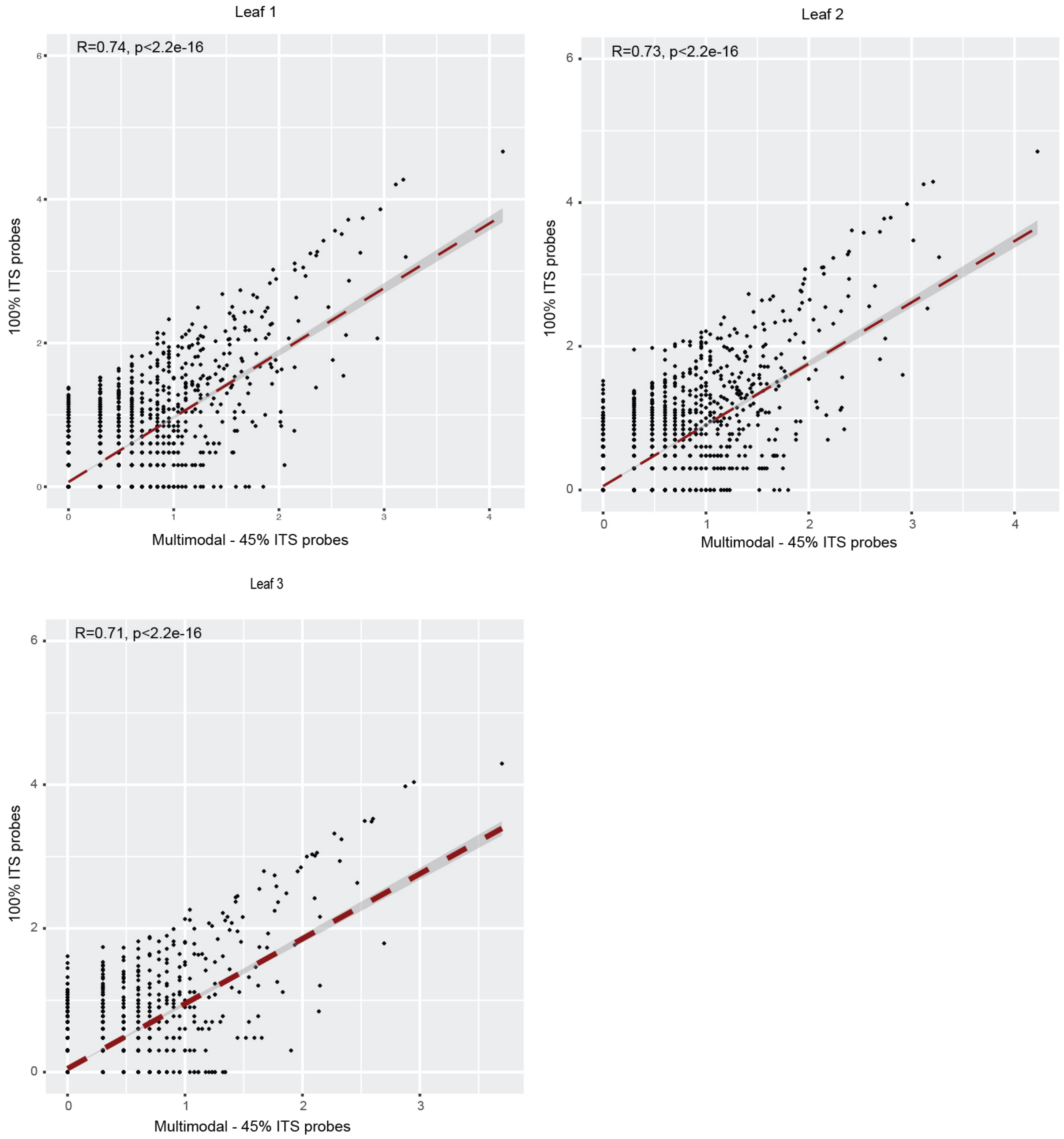

Supplementary Fig. 12: Scatter plots of log normalised unique fungal molecules and the corresponding Pearson correlation and the corresponding two-tailed significance test for arrays with 100% and 45% 18S rRNA/ITS probes in leaves 1 to 3. For leaves 1, 2, and 3 the Pearson correlation values are 0.74, 0.73, and 0.71 ($p<2.2e-16$), respectively. The red line shows the fitted linear model with 0.95 confidence interval.

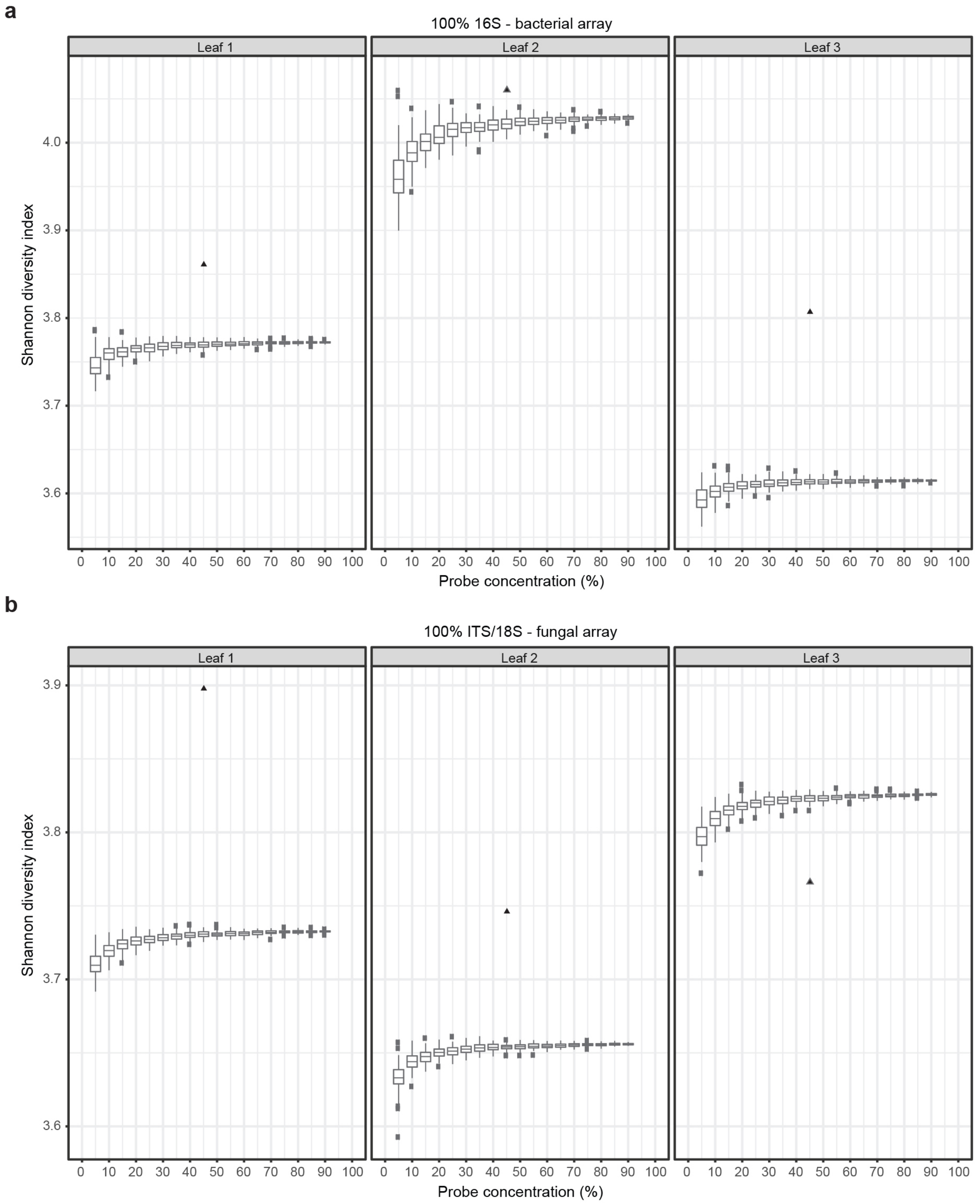

Supplementary Fig. 13: Shannon diversity index as a function of the simulated probe concentration performed by downsampling for 16S-capturing (**a**) and ITS/18S-capturing (**b**) probes. Boxplot for each probe concentration illustrates the median, first and third quartiles of the calculated Shannon diversity index, with whiskers extending 1.5 times the interquartile range. Each boxplot represent 100 repeats of the downsampling simulation (see **Methods** for more details).

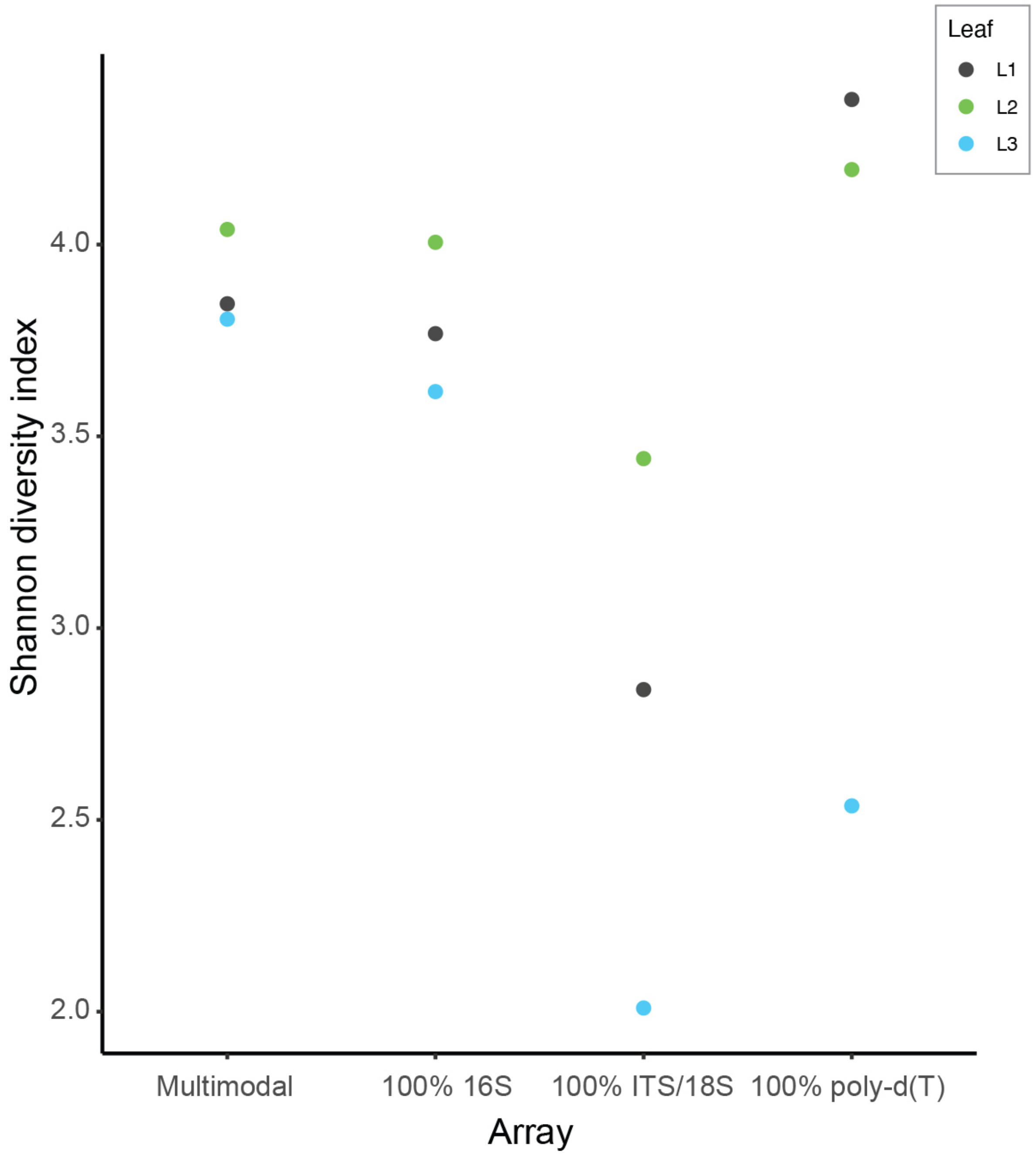

Supplementary Fig. 14: Shannon diversity index between different leaves in different arrays with bacterial assignment. L1, L2 and L3 stands for Leaf 1, Leaf 2 and Leaf 3, respectively.

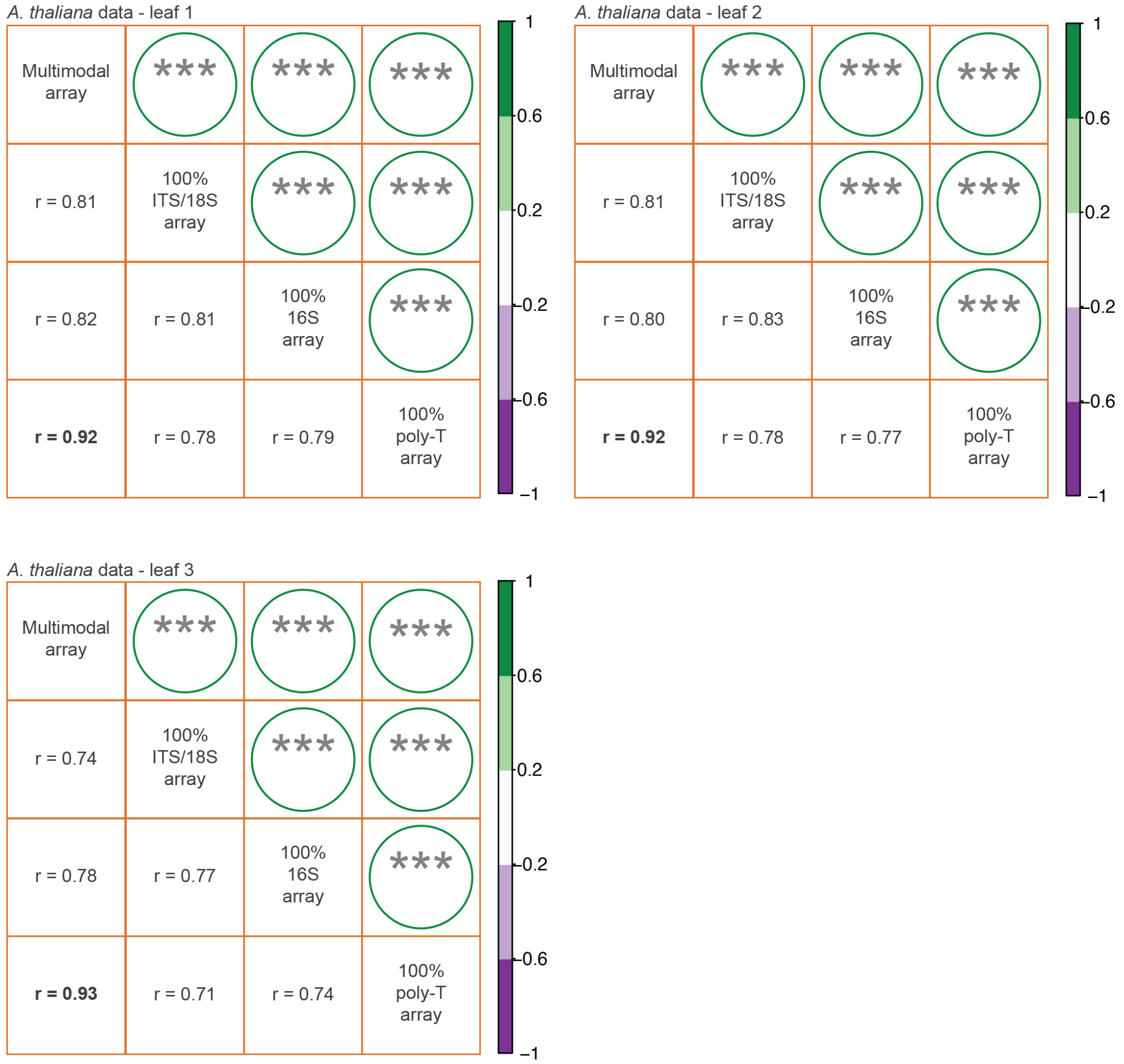

Supplementary Fig. 15: Pairwise Pearson correlation coefficients and the corresponding two-tailed significance test of the ribosomal, chloroplast, mitochondrial and non-coding filtered host *A. thaliana* component between different array types in three leaves. *** indicates the p-value=0. Correlation between the multimodal array and the 100% poly-d(T) array is in bold.

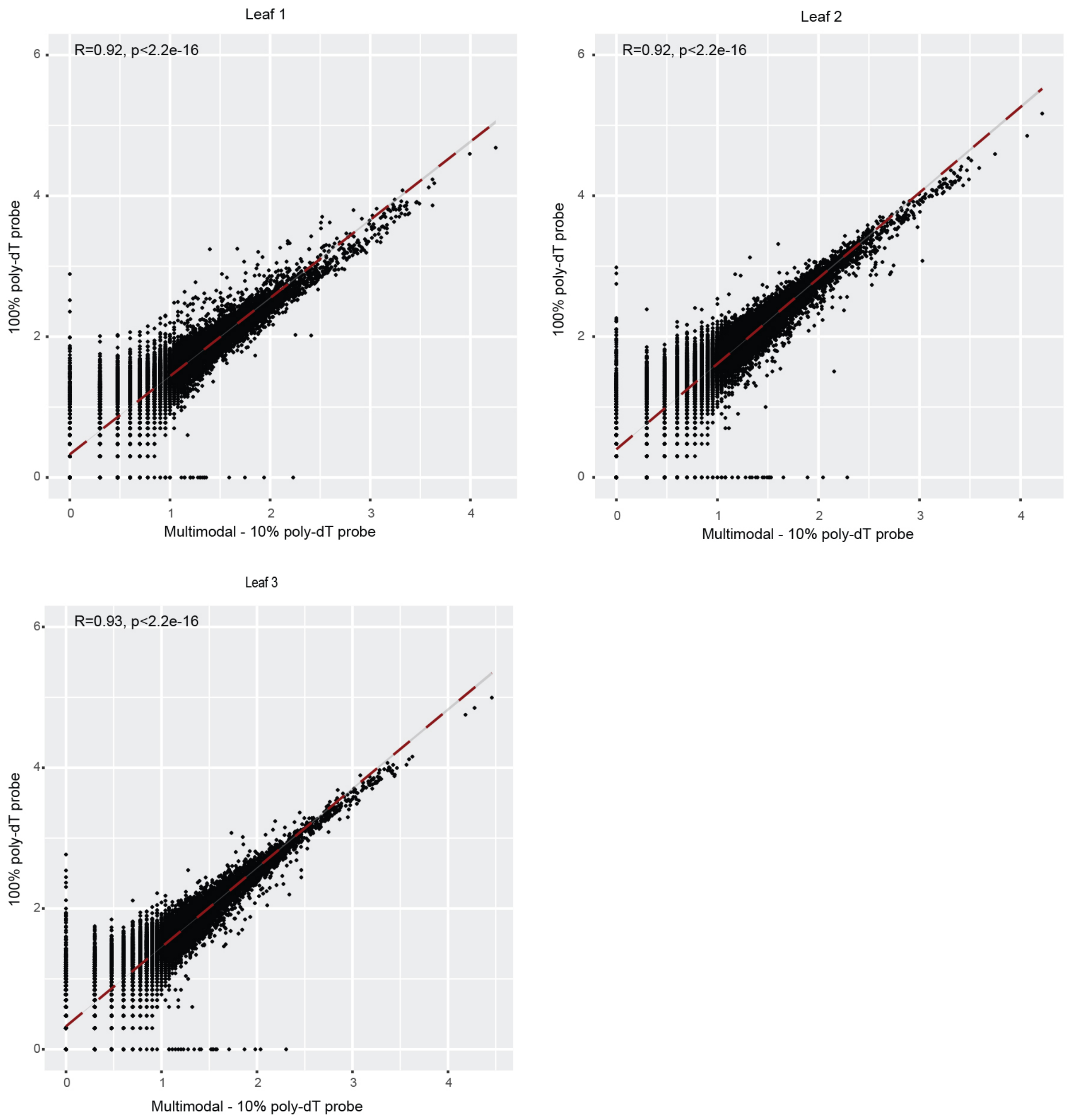

Supplementary Fig. 16. Scatter plots of log normalised unique host molecules and the corresponding Pearson correlation and the corresponding two-tailed significance test for arrays with 100% poly-(d)T and 10% poly-(d)T in leaves 1 to 3. For leaves 1, 2, and 3 the Pearson correlation values are 0.92, 0.92, and 0.93 ($p<2.2e-16$), respectively. The red line shows the fitted linear model with 0.95 confidence interval.

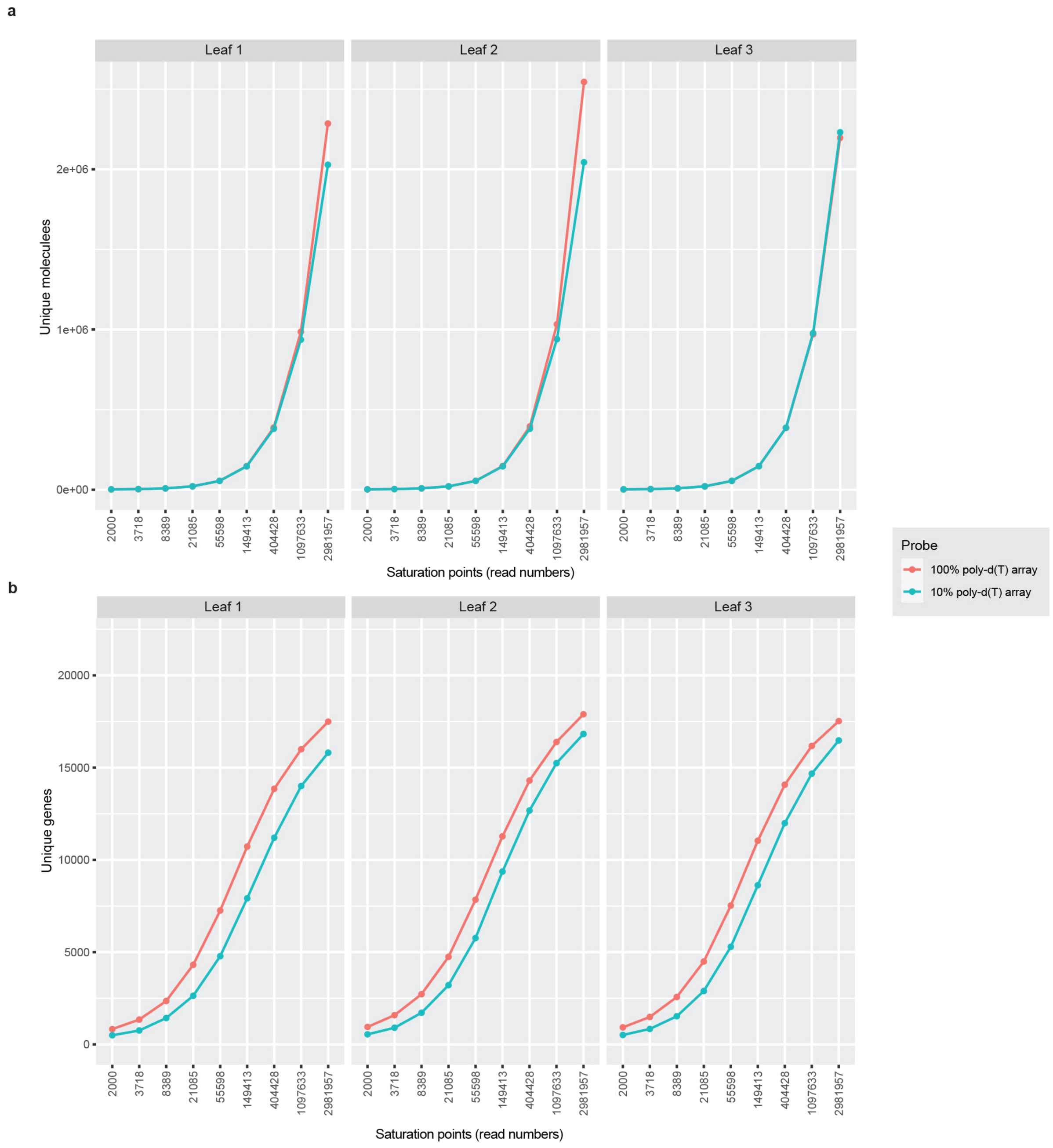

Supplementary Fig. 17: Saturation plots of the unique molecules (**a**) and genes (**b**) in 100% and 10% poly-d(T) (multimodal) array for three replicates.

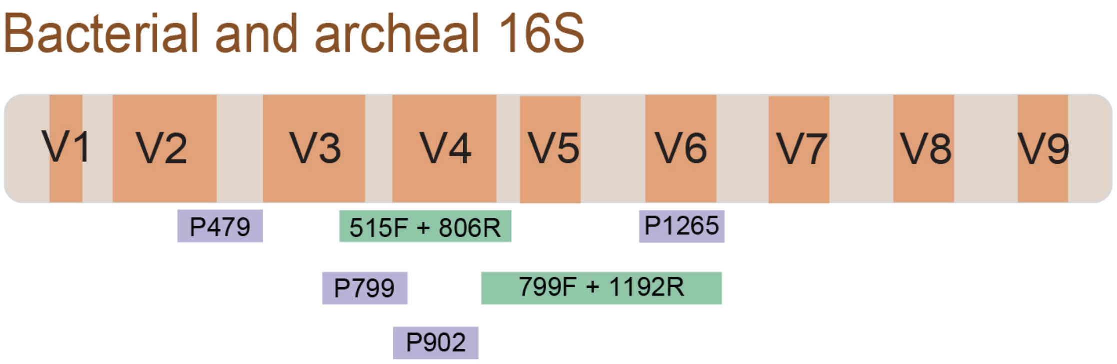

Supplementary Fig. 18: Bacterial and archeal target regions of the SmT probes (purple) and amp-seq primers (green).

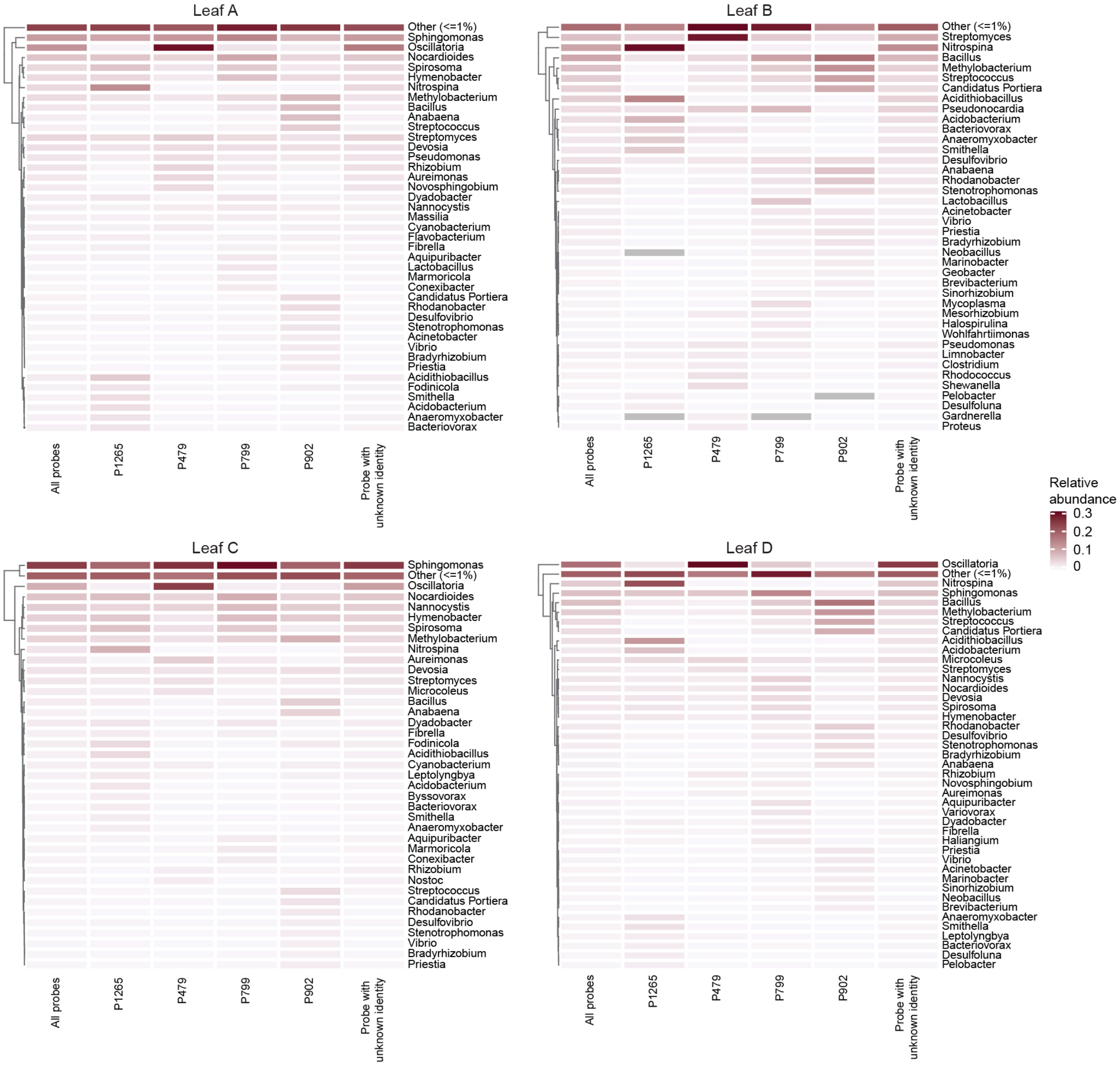

Supplementary Fig. 19: Bacterial relative abundance profiles of four leaf crude extracts (**a-d**), as captured by each of the four different probes (the initial P799 and P902 probes and two additional ones - P479 and P1265) and the unknown probes (reads that their probe of origin could not be detected), and the final profile that derived from all probes.

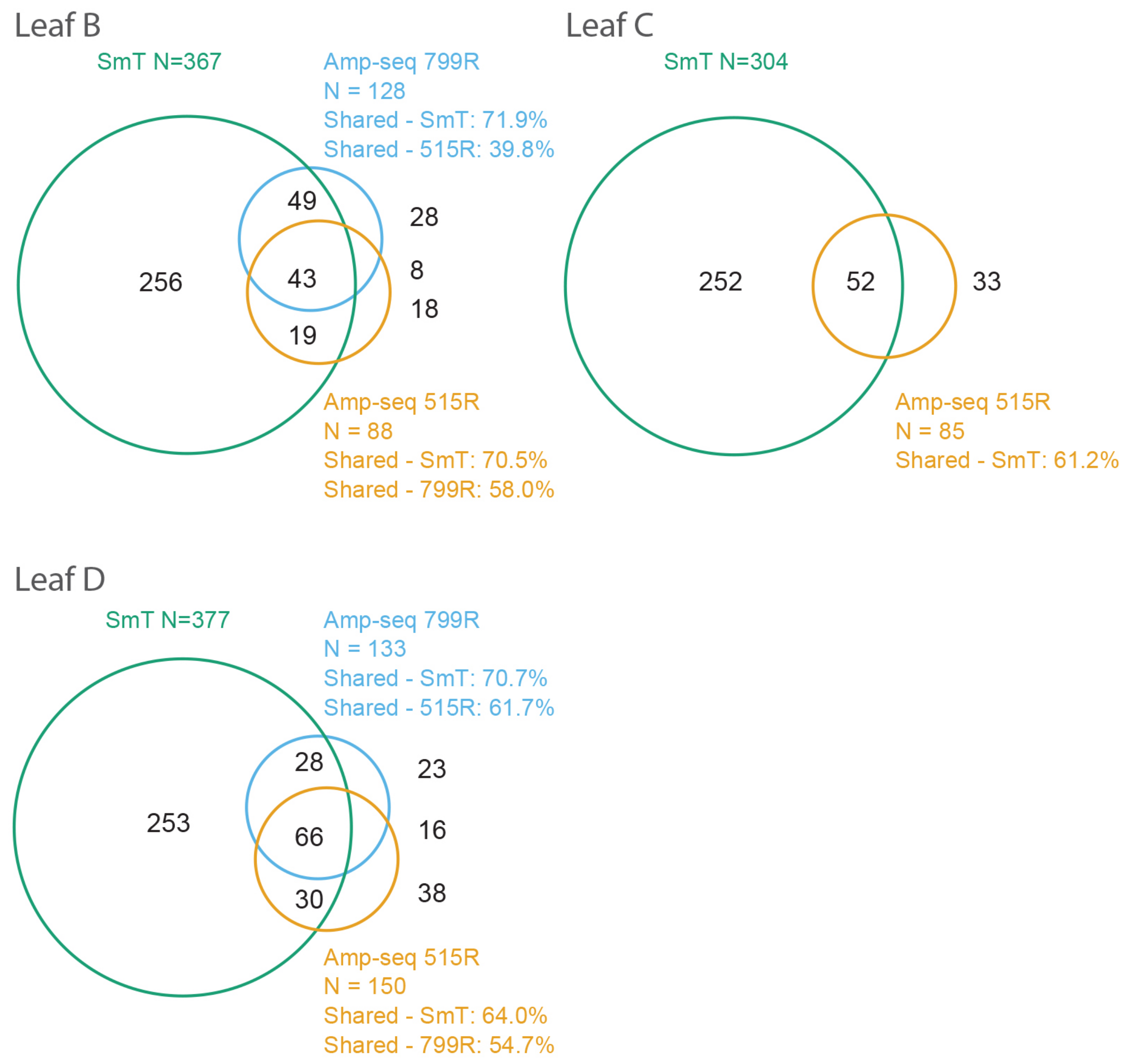

Supplementary Fig. 20: Taxa identified in different biological replicates presented in Venn diagrams presenting average values of subset samples after 100 iterations.

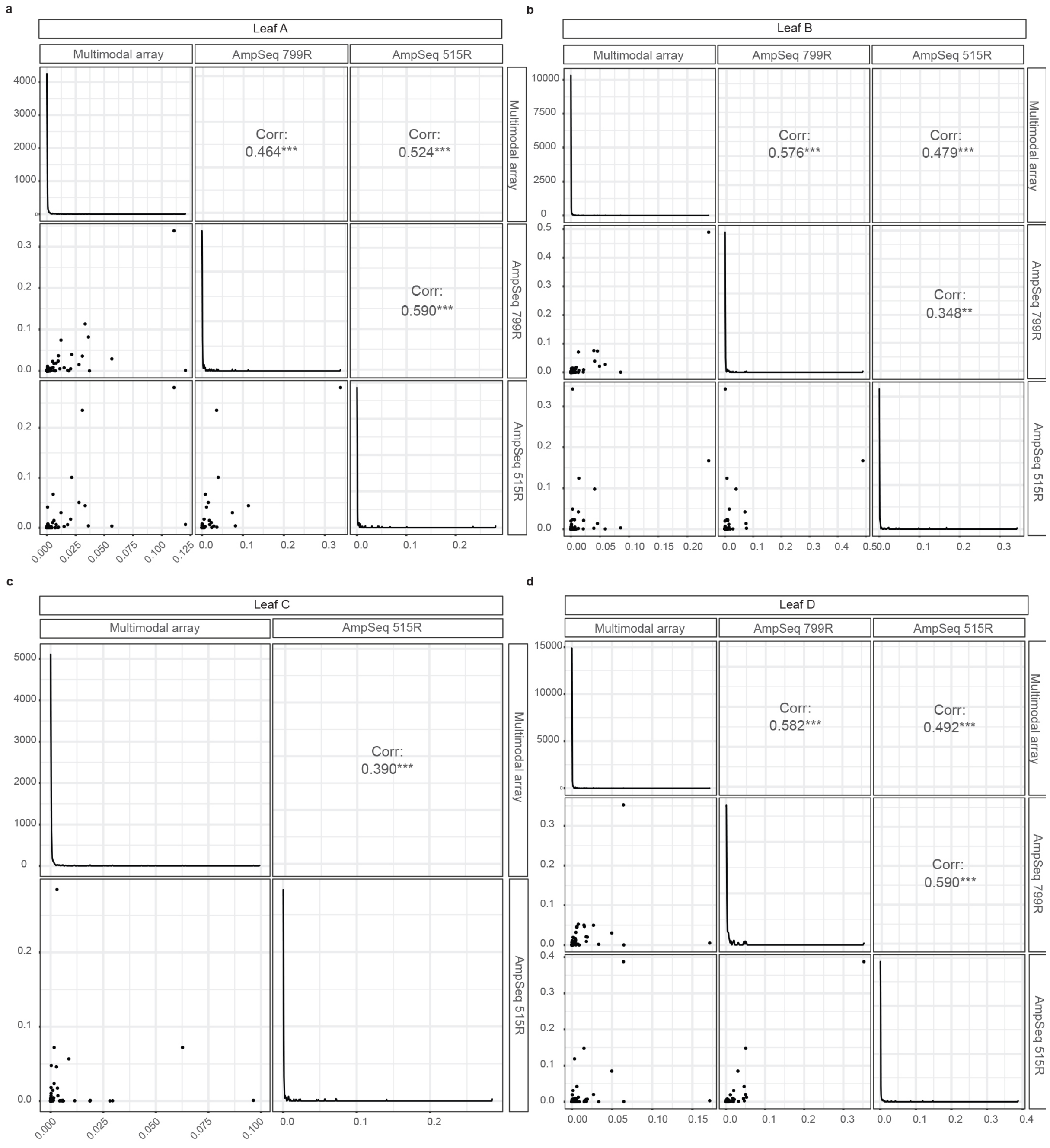

Supplementary Fig. 21: Spearman correlation coefficients of the relative abundance of shared bacterial genera, comparing all pairwise combinations between the multimodal array, amp-seq 799R and amp-seq 515R primer pairs, across four leaf crude extracts (**a-d**). In all cases p-value<0.001 (two-tailed test) denoted by ‘***’.

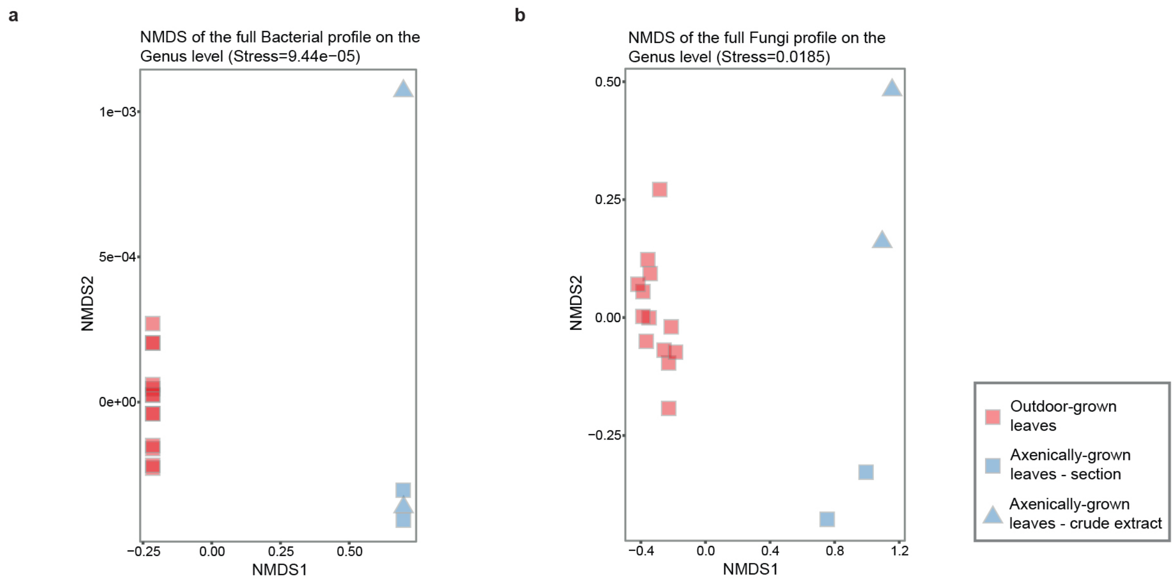

Supplementary Fig. 22: Analyses of axenically-grown leaves as a negative control. Non-metric multidimensional scaling (NMDS) of Bray-Curtis similarity for (a) bacterial and (b) fungal taxa between sections of outdoor-grown leaves and axenically-grown leaves (both sections and crude extracts, as indicated by the different shapes).

Outdoor and axenically-grown leaves - bacteria

Taxon	P2.L2.1	P2.L2.2	P2.L1.3	P2.L1.4	P2.L2.4	P2.L1.1	P1.L2.4	P1.L1.1	P1.L2.2	P1.L1.4	P1.L2.1	P1.L1.2	P2.L2.3	S2.L2	S2.L1
Other (<=1%)	22.38	23.14	25.14	24.49	25.12	23.66	23.17	24.59	27.45	25.40	26.32	26.02	28.48	20.09	15.79
Nitrospina	19.93	19.45	14.37	14.36	13.13	16.83	15.46	14.93	10.66	11.35	11.61	12.24	13.01	8.56	5.22
Bacillus	7.95	7.84	8.97	8.78	8.82	9.32	10.00	9.75	9.32	9.19	9.44	8.93	7.47	6.26	4.56
Acidithiobacillus	10.04	9.30	6.49	6.56	6.56	7.50	7.19	7.15	4.41	4.29	5.09	5.32	6.04	2.22	1.33
Methylobacterium	4.98	4.89	5.66	5.62	5.56	5.72	5.92	5.59	5.56	6.62	5.95	5.87	4.35	3.64	2.46
Streptomyces	2.62	2.59	3.86	4.85	4.19	2.22	2.40	2.85	5.77	3.75	4.85	4.98	5.36	3.20	3.91
Streptococcus	4.10	4.05	5.31	5.21	5.49	4.65	4.85	4.63	4.70	4.71	4.84	4.34	4.11	1.13	0.76
Candidatus Portiera	3.04	3.07	3.52	3.57	3.87	3.72	3.86	3.73	3.88	4.02	3.98	4.00	3.14	2.52	1.98
Stenotrophomonas	2.60	2.51	3.01	2.89	2.87	2.83	2.97	2.88	2.59	2.94	2.85	2.72	2.12	1.20	0.84
Anabaena	2.27	2.68	2.24	2.42	2.11	2.49	2.33	2.25	2.64	2.96	2.49	2.51	2.60	1.77	1.21
Rhodanobacter	2.14	2.05	2.38	2.39	2.47	2.37	2.53	2.37	2.52	2.71	2.47	2.36	1.87	1.33	0.79
Vibrio	2.26	2.11	2.07	1.81	1.65	2.57	2.44	2.33	1.94	2.55	2.25	2.17	1.40	1.78	1.04
Desulfovibrio	1.86	2.01	1.97	2.15	2.10	1.97	1.96	1.76	1.93	2.31	1.76	1.98	1.87	0.84	0.51
Bradyrhizobium	1.51	1.51	1.55	1.57	1.46	1.70	1.60	1.56	1.63	1.68	1.38	1.44	1.27	1.17	0.83
Acinetobacter	1.23	1.20	1.37	1.33	1.32	1.40	1.57	1.40	1.55	1.81	1.64	1.72	1.19	1.50	1.09
Acidobacterium	1.73	1.81	1.38	1.41	1.52	1.67	0.95	0.87	0.70	0.77	0.77	0.81	1.56	0.59	0.28
Mycoplasma	0.85	0.86	1.03	0.98	0.95	0.72	1.43	1.14	1.55	1.51	1.40	1.52	0.94	1.34	1.03
Smithella	0.63	0.75	0.93	0.96	0.76	0.67	0.75	0.59	1.52	1.62	1.29	1.53	0.84	1.20	1.46
Clostridium	0.59	0.78	0.99	0.86	0.62	0.90	0.75	0.90	1.48	1.41	1.32	1.39	1.19	0.89	0.82
Priestia	1.14	1.07	1.14	1.08	1.16	1.20	1.33	1.32	1.23	1.49	1.27	1.19	0.87	1.09	0.64
Marinobacter	1.03	0.91	0.89	0.82	0.78	1.08	1.37	1.12	0.93	1.31	1.12	1.25	0.58	1.06	0.71
Pseudomonas	1.04	1.21	1.08	1.04	1.01	1.04	0.91	0.93	0.91	1.03	0.91	0.94	1.16	0.80	0.59
Geobacter	0.69	0.69	1.00	1.06	1.11	0.82	0.74	0.69	0.91	0.93	0.87	0.76	0.73	0.58	0.43
Lactobacillus	0.43	0.58	0.84	0.86	0.64	0.50	0.44	0.36	0.84	0.93	0.71	1.01	0.67	1.20	1.36
Halospirulina	0.34	0.50	0.54	0.59	0.89	0.42	0.52	0.69	0.99	0.94	0.89	0.86	1.59	0.77	0.62
Cyanothece	0.26	0.31	0.47	0.35	0.28	0.40	0.69	1.08	0.91	0.72	0.95	0.86	0.62	0.88	0.64
Flavobacterium	0.41	0.40	0.44	0.35	0.37	0.54	0.77	1.04	0.60	0.54	0.76	0.63	0.44	0.47	0.25
Rhodobium	1.25	0.81	0.46	0.36	0.98	0.53	0.73	1.07	0.39	0.16	0.40	0.29	2.00	0.05	0.02
Shewanella	0.40	0.51	0.47	0.68	1.22	0.29	0.17	0.18	0.20	0.15	0.19	0.15	1.32	0.07	0.05
Proteus	0.29	0.38	0.40	0.55	0.95	0.25	0.19	0.21	0.21	0.16	0.20	0.17	1.18	0.10	0.06
Paenibacillus	0.03	0.03	0.04	0.03	0.03	0.03	0.04	0.04	0.04	0.04	0.04	0.03	0.03	31.70	48.72

Relative abundance (%)
40
30
20
10
0

Supplementary Fig. 23: Relative abundance of the bacterial taxa and their hierarchical clustering in the whole tissue area in axenically-grown and outdoor-grown leaves. 'P' - plant, 'L' - leaf.

Outdoor and axenically-grown leaves - fungi

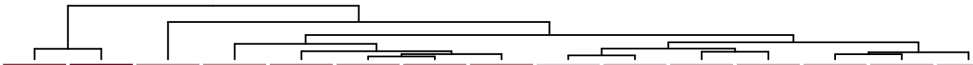

S2.L2	S2.L1	P2.L2.4	P2.L2.3	P1.L2.1	P1.L1.4	P1.L1.2	P1.L2.2	P2.L2.1	P2.L1.1	P1.L2.4	P1.L1.1	P2.L1.3	P2.L1.4	P2.L2.2	
33.86	39.99	18.98	19.67	21.89	18.92	20.74	22.09	8.42	11.28	12.88	13.99	13.11	12.75	10.06	Other (<=1%)
0.00	0.00	0.43	30.95	18.21	24.20	24.41	22.39	18.71	19.33	14.23	14.79	22.62	26.81	26.09	Phaeosphaeria
9.20	4.51	16.12	9.52	11.65	12.42	11.25	10.54	17.58	16.77	15.84	17.76	12.30	11.60	15.51	Rhizophagus
5.97	3.00	12.62	7.42	10.02	10.85	10.20	9.71	14.57	14.06	13.07	13.85	11.62	10.77	12.75	Chloromonas
1.47	1.27	10.46	4.64	5.68	4.08	4.06	4.63	5.31	5.22	5.70	4.96	6.05	6.14	4.70	Femsjonia
2.21	1.08	6.69	3.92	4.97	4.52	4.22	4.11	7.15	6.52	6.04	6.42	6.95	6.45	5.87	Amanita
1.80	1.38	6.42	3.58	3.90	3.42	3.37	3.52	5.74	5.13	4.72	4.82	5.59	5.14	4.78	Elaphomyces
0.96	0.36	4.34	2.28	2.70	2.36	2.49	2.26	4.19	3.34	2.81	2.82	3.86	3.64	3.36	Roccella
0.67	0.58	3.12	1.72	2.30	2.34	1.93	2.07	3.53	3.06	2.65	2.71	3.17	3.09	2.83	Helminthosporium
0.81	0.34	2.52	1.29	1.75	1.87	1.73	1.52	2.67	2.36	2.20	2.63	1.88	1.68	2.23	Marasmius
0.48	0.19	2.10	0.96	1.85	1.75	1.50	1.51	2.37	2.27	2.50	2.54	1.86	1.81	1.96	Geopora
0.97	0.44	2.04	1.13	1.54	1.61	1.61	1.35	2.06	2.00	1.93	2.36	1.52	1.29	1.80	Elmerina
0.25	0.13	2.37	1.14	1.22	1.14	1.03	1.23	1.86	1.59	1.24	1.44	1.79	1.81	1.49	Willeya
3.07	5.71	0.85	2.57	2.67	2.15	2.68	3.12	0.34	0.69	1.15	1.85	0.98	0.71	0.67	Plasmodium
3.72	5.27	0.09	0.13	0.08	0.08	0.13	0.08	0.01	0.02	0.02	0.02	0.03	0.04	0.02	Gregarina
1.84	2.47	2.06	1.80	0.90	1.13	0.89	1.24	0.33	0.59	0.50	0.34	0.67	0.90	0.80	Saccharomyces
2.74	2.49	0.65	1.67	1.50	1.25	1.45	1.63	0.26	0.68	0.56	0.96	0.83	0.65	0.44	Aspergillus
1.31	1.92	0.43	1.36	1.38	1.01	1.37	1.67	0.20	0.35	0.49	0.85	0.46	0.41	0.33	Strongyloides
3.07	2.19	0.10	0.09	0.09	0.07	0.12	0.17	0.05	0.07	0.22	0.05	0.10	0.08	0.07	Chlamydomonas
1.41	1.72	0.16	0.35	0.46	0.40	0.42	0.34	0.04	0.09	0.13	0.17	0.20	0.16	0.08	Albugo
1.97	2.43	0.12	0.17	0.19	0.17	0.26	0.19	0.03	0.08	0.08	0.09	0.16	0.08	0.06	Clavispora
0.78	1.10	0.08	0.33	0.19	0.27	0.41	0.21	0.02	0.08	0.03	0.07	0.20	0.14	0.04	Compsopogon
0.32	1.36	0.01	0.03	0.11	0.08	0.09	0.12	0.00	0.01	0.05	0.07	0.00	0.01	0.00	Tritia
1.46	1.12	0.08	0.16	0.19	0.15	0.15	0.19	0.03	0.05	0.07	0.10	0.08	0.06	0.04	Chlorella
1.53	1.18	0.04	0.04	0.06	0.08	0.04	0.05	0.03	0.03	0.02	0.05	0.05	0.05	0.03	Pyramimonas
1.09	0.58	0.02	0.02	0.06	0.14	0.05	0.07	0.01	0.00	0.04	0.09	0.00	0.01	0.01	Ostreobium
0.17	0.06	1.13	0.55	1.04	0.96	0.99	0.81	1.18	1.24	1.27	1.08	1.20	1.06	1.03	Phlyctochytrium
0.43	0.22	1.24	0.70	0.84	0.76	0.67	0.68	1.10	1.03	0.90	0.89	0.84	0.78	0.94	Talaromyces
0.34	0.18	0.97	0.47	0.75	0.67	0.73	0.81	0.90	0.83	1.06	1.03	0.59	0.52	0.79	Phellinus
0.27	0.36	1.07	0.46	0.64	0.40	0.44	0.52	0.62	0.55	0.61	0.56	0.62	0.65	0.55	Malassezia
0.07	0.10	1.10	0.46	0.37	0.34	0.15	0.37	0.49	0.38	0.39	0.34	0.45	0.44	0.44	Trichophyton
0.10	0.01	1.58	0.41	0.60	0.33	0.37	0.42	0.21	0.26	0.56	0.32	0.21	0.23	0.21	Phyllozyma
0.00	0.00	0.00	0.01	0.18	0.01	0.01	0.36	0.00	0.00	6.02	0.00	0.02	0.00	0.00	Dictyochloris
15.65	16.25	0.02	0.03	0.02	0.05	0.03	0.03	0.02	0.01	0.02	0.00	0.01	0.02	0.02	Peltigera

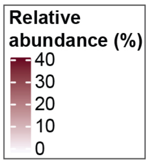

Supplementary Fig. 24: Relative abundance of the bacterial taxa and their hierarchical clustering in the whole tissue area in axenically-grown and outdoor-grown leaves. ‘P’ - plant, ‘L’ - leaf.

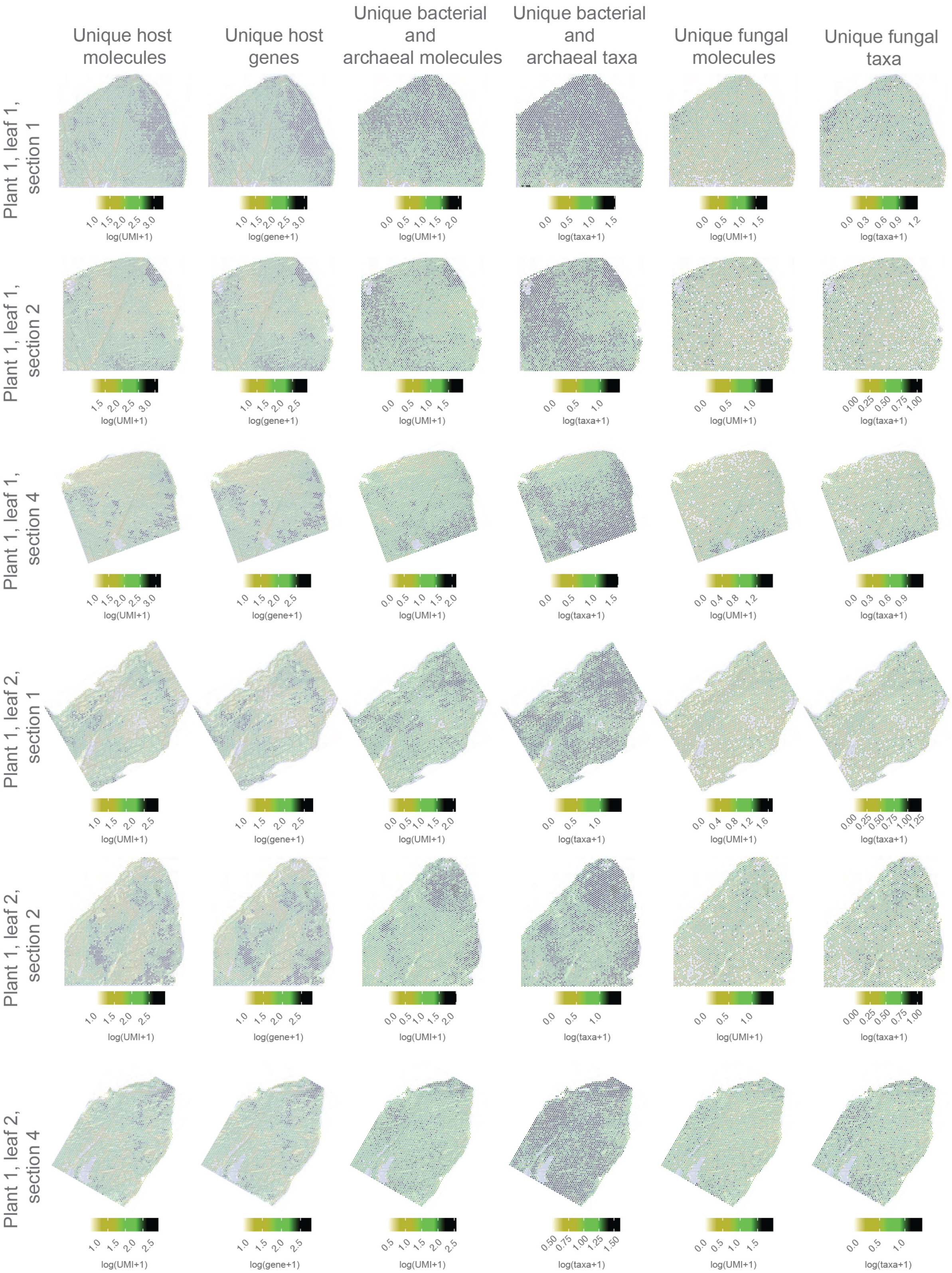

Unique host molecules
Unique host genes
Unique bacterial and archaeal molecules
Unique bacterial and archaeal taxa
Unique fungal molecules
Unique fungal taxa
Plant 1, leaf 1, section 1
Plant 1, leaf 1, section 2
Plant 1, leaf 1, section 4
Plant 1, leaf 2, section 1
Plant 1, leaf 2, section 2
Plant 1, leaf 2, section 4
log(UMI+1)
log(gene+1)
log(taxa+1)

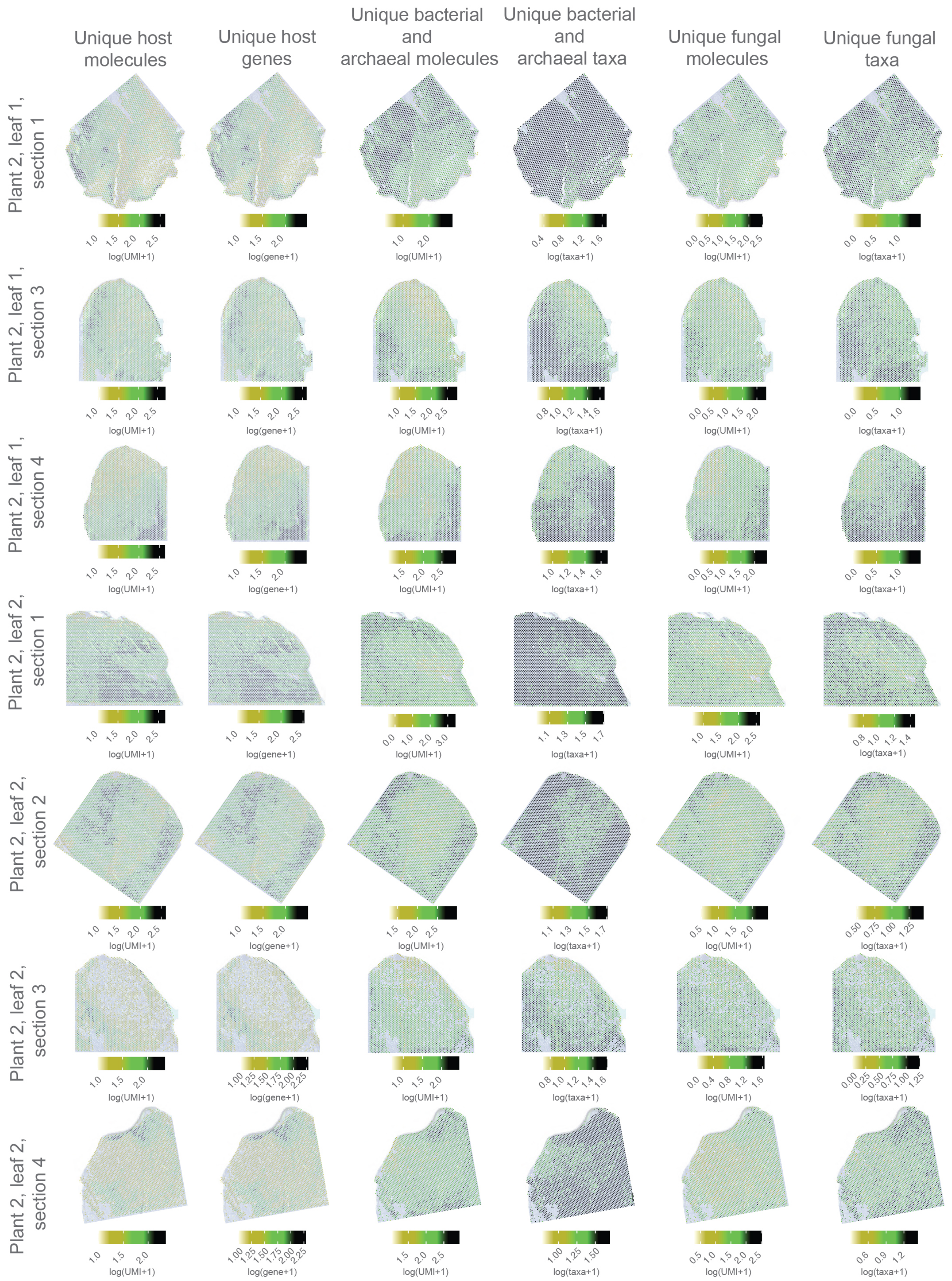

Supplementary Fig. 25: Unique host molecules, unique genes, unique bacterial and archaeal molecules, unique bacterial and archaeal taxa, unique fungal molecules and unique fungal taxa in $\log_{10}$ scale for all the sections in the dataset.

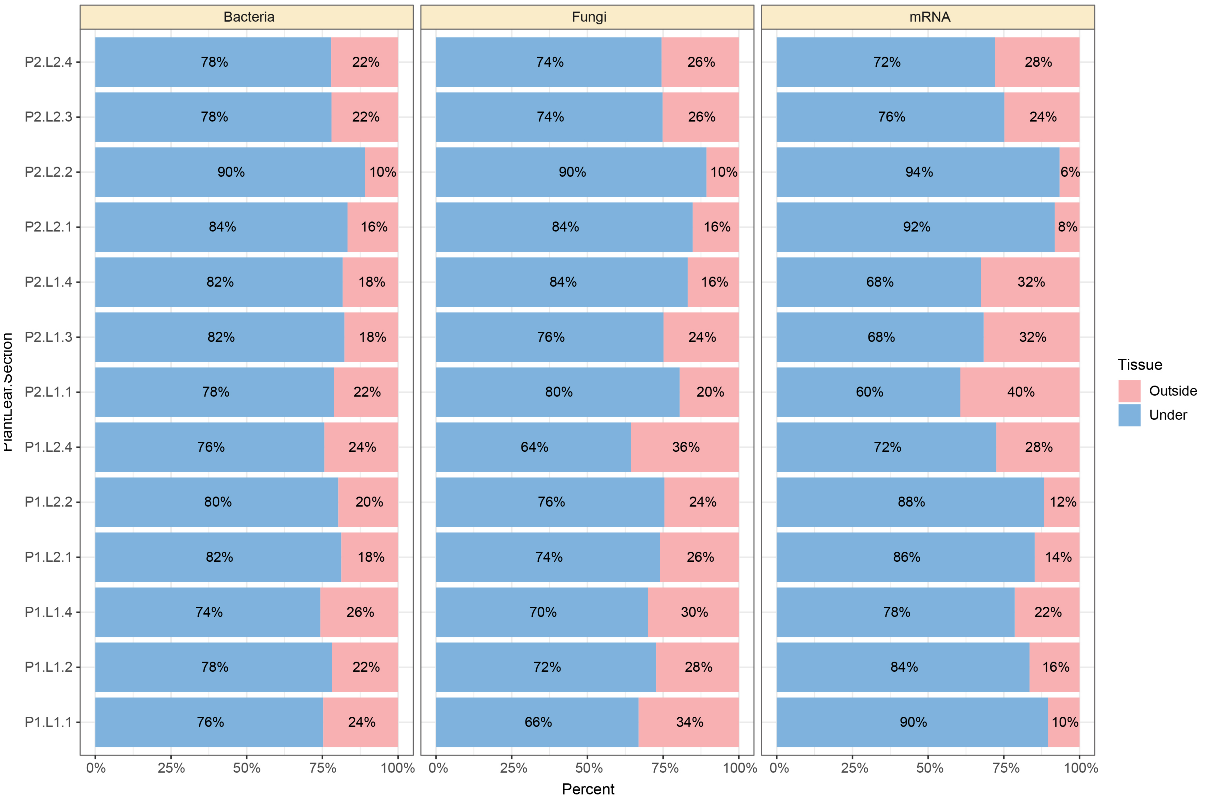

Supplementary Fig. 26: Proportion of unique molecules under the tissue and outside the tissue in each of the leaf sections.

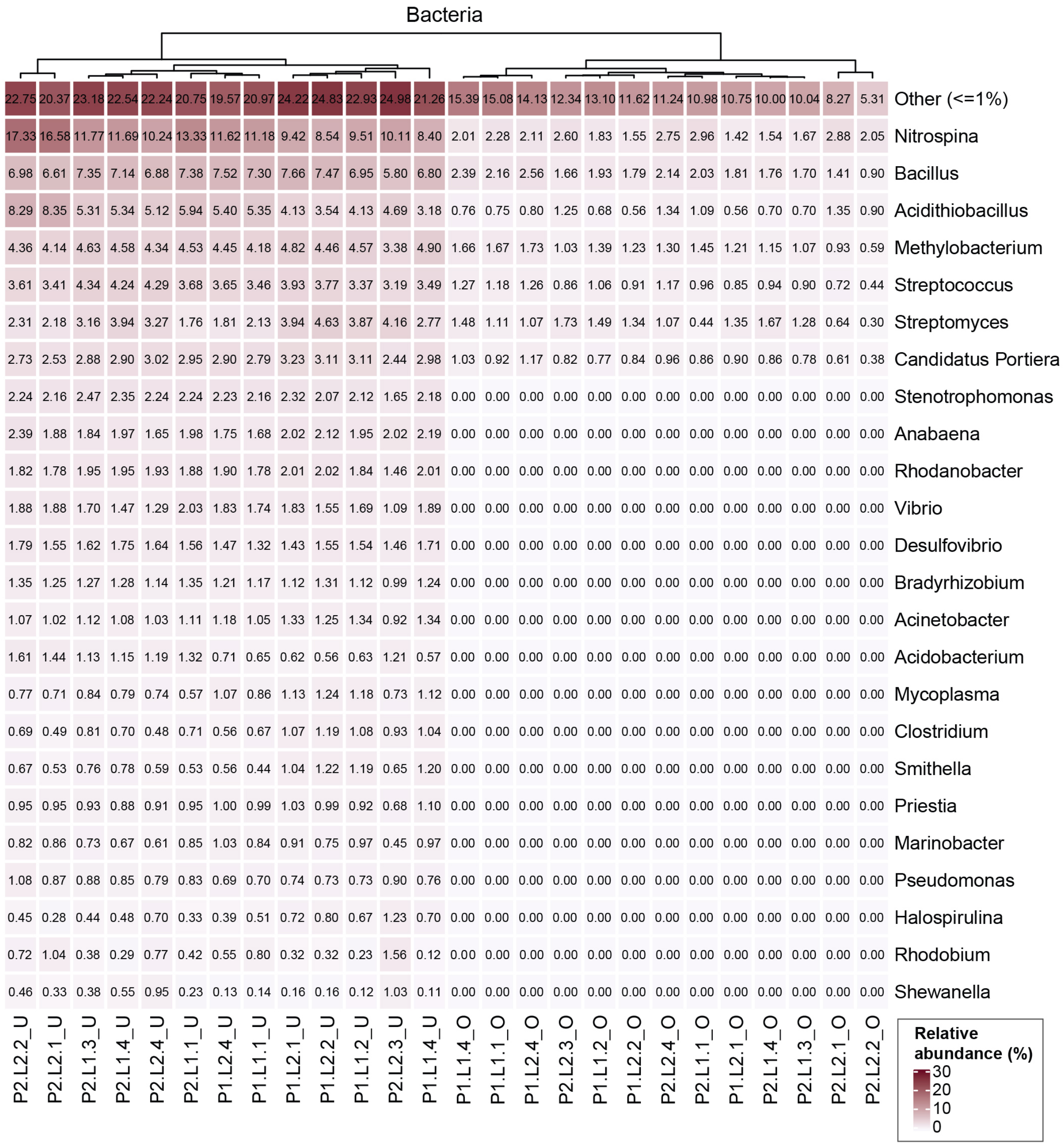

Supplementary Fig. 27: Relative bacterial abundance profiles under- and outside-the-tissue grouped by hierarchical clustering. The suffix 'O' refers to outside-the-tissue and the suffix 'U' refers to under-the-tissue.

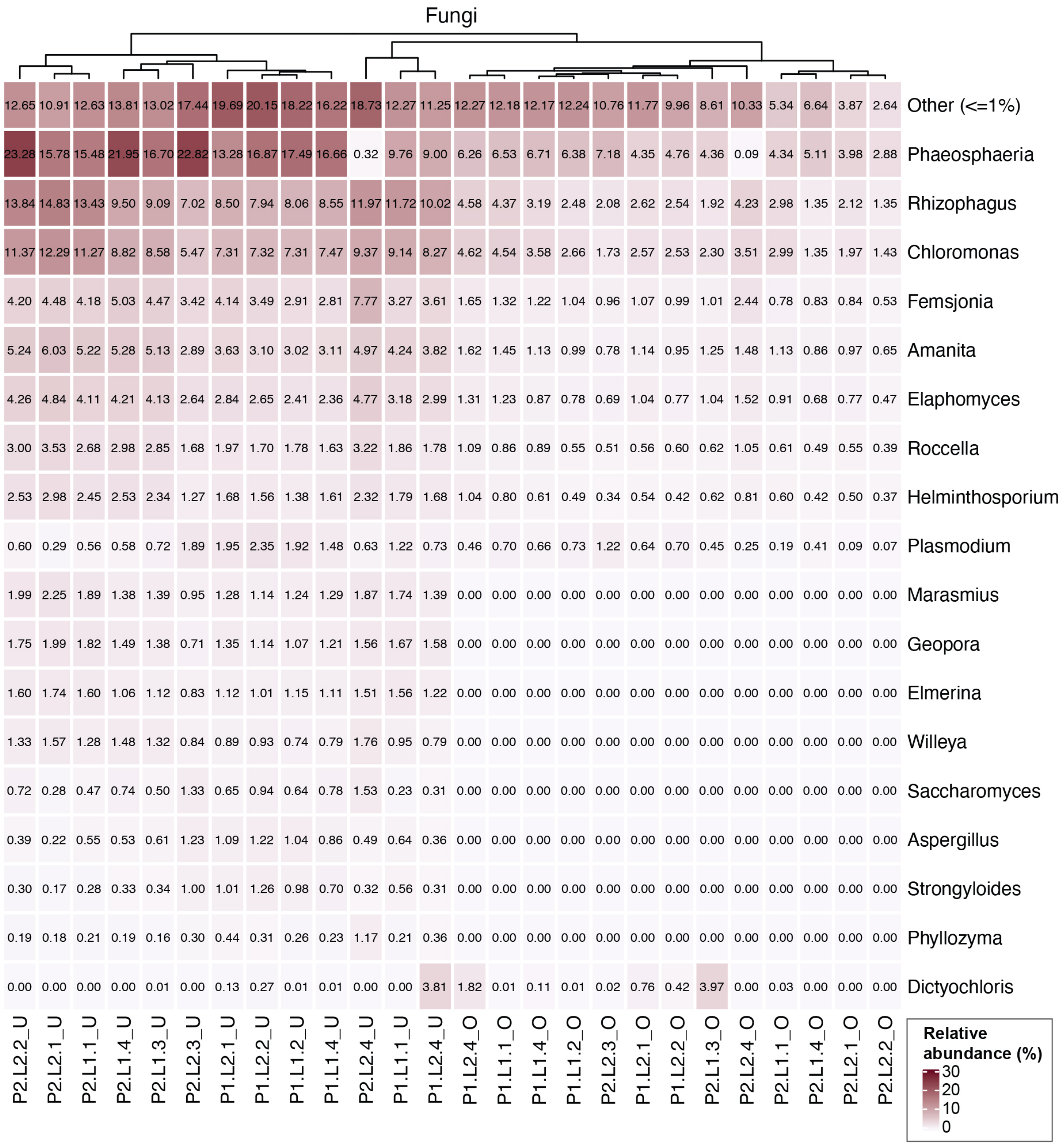

Supplementary Fig. 28: Relative fungal abundance profiles under- and outside-the-tissue grouped with hierarchical clustering. The suffix 'O' refers to outside-the-tissue and the suffix 'U' refers to under-the-tissue.

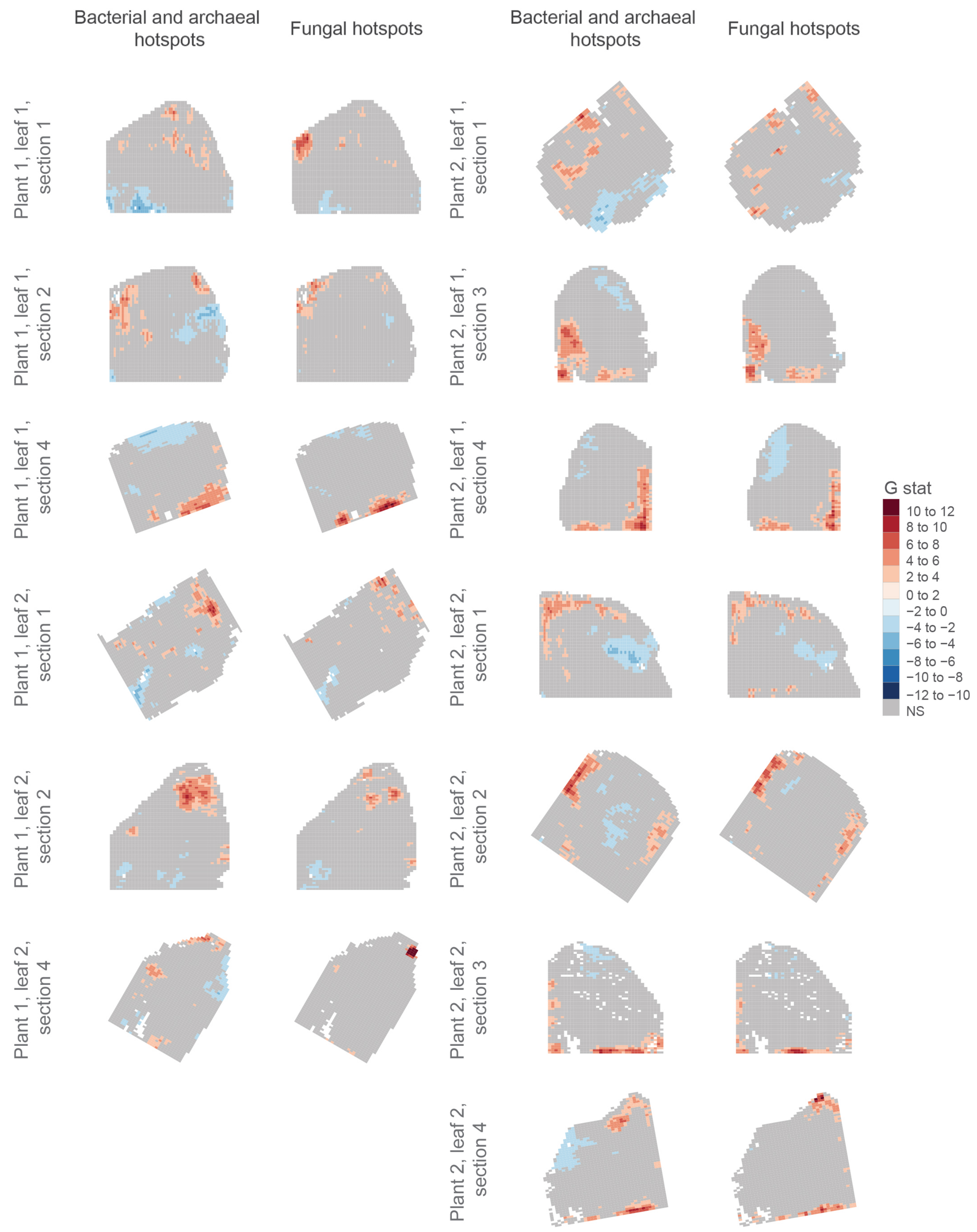

Supplementary Fig. 29: Bacterial and archaeal and fungal hotspots for each of the leaf sections.

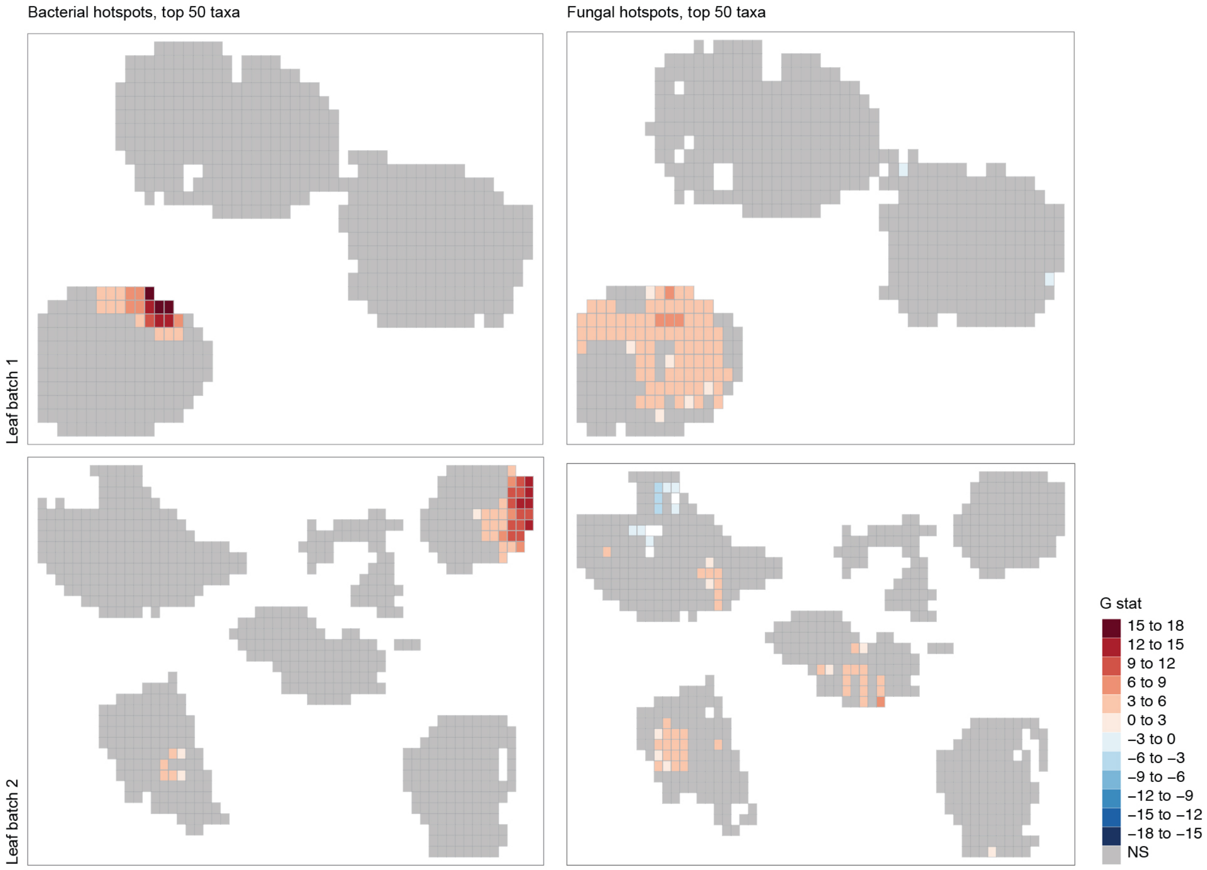

Supplementary Fig. 30: Significant hot- and cold-spots for bacteria and fungi in axenically-grown leaf sections.

Outdoor and axenically-grown leaves - bacteria

Taxon	S2.L1	S2.L2	P2.L2.2	P2.L2.1	P1.L1.4	P1.L1.2	P1.L2.1	P1.L2.2	P2.L2.4	P1.L1.1	P1.L2.4	P2.L2.3	P2.L1.4	P2.L1.1	P2.L1.3
Paenibacillus	93.00	83.11	0.00	0.00	0.00	0.00	0.00	0.00	0.00	0.00	0.00	0.00	0.00	0.00	0.00
Nitrospina	0.86	2.50	24.49	23.95	14.27	14.09	15.23	14.86	17.93	17.96	17.41	19.10	21.45	20.87	19.72
Bacillus	1.80	2.89	8.88	8.75	10.80	10.15	11.17	10.14	10.41	11.12	11.64	9.24	9.90	10.75	10.32
Other (<=1%)	0.64	2.20	8.65	8.65	9.22	9.32	9.68	9.80	9.64	9.30	8.42	8.49	8.66	9.09	8.77
Acidithiobacillus	0.25	0.65	11.97	12.34	5.38	6.48	6.52	6.81	8.46	8.40	8.05	8.25	9.87	9.67	9.06
Methylobacterium	0.38	0.80	5.45	5.51	7.93	7.51	6.40	5.38	6.61	6.51	7.02	5.32	6.32	6.38	6.63
Streptococcus	0.12	0.37	4.30	4.68	5.16	5.04	5.32	4.82	5.97	5.88	5.67	4.18	5.13	5.26	5.70
Candidatus.Portiera	0.24	0.41	3.31	3.30	4.76	4.61	4.66	3.88	4.33	4.41	4.60	3.64	3.77	4.18	4.05
Streptomyces	0.39	0.77	2.56	3.04	4.36	6.47	5.16	6.13	4.20	3.34	3.06	5.31	3.87	2.45	3.43
Anabaena	0.15	0.56	3.21	2.37	3.24	3.25	3.16	3.82	2.51	2.52	2.82	3.40	3.08	2.82	2.71
Stenotrophomonas	0.17	0.25	2.76	2.90	3.47	3.02	3.20	2.87	3.18	3.38	3.26	2.66	3.17	3.24	3.39
Vibrio	0.09	0.50	2.59	2.29	3.16	2.51	3.03	2.54	2.32	2.86	3.06	2.54	3.08	2.91	3.03
Rhodanobacter	0.12	0.24	2.29	2.38	3.18	2.60	2.62	2.52	2.88	2.56	3.16	2.22	2.55	2.79	2.74
Desulfovibrio	0.10	0.24	2.28	2.01	2.56	2.32	2.03	2.10	2.41	2.21	2.28	2.40	2.27	2.36	2.14
Bradyrhizobium	0.07	0.25	1.72	1.62	2.14	1.66	1.42	1.73	1.82	2.09	1.83	1.88	1.94	1.98	1.87
Acinetobacter	0.15	0.48	1.27	1.29	2.30	2.11	2.16	1.95	1.54	1.51	1.95	1.64	1.56	1.62	1.72
Acidobacterium	0.03	0.26	2.22	2.06	0.94	1.11	1.12	1.05	1.82	0.99	0.99	2.14	1.99	2.01	1.85
Rhodobium	0.00	0.00	1.23	2.04	0.00	0.00	0.48	0.87	1.38	1.47	0.82	2.77	0.56	0.78	0.84
Priestia	0.11	0.35	1.24	1.32	1.87	1.34	1.35	1.34	1.33	1.36	1.49	1.28	1.41	1.34	1.44
Marinobacter	0.16	0.37	1.10	1.08	1.59	1.54	1.37	1.16	1.07	1.25	1.69	1.01	1.32	1.24	1.34
Pseudomonas	0.09	0.29	1.37	1.25	1.27	1.13	1.00	1.12	1.22	1.17	1.02	1.38	1.20	1.26	1.26
Mycoplasma	0.09	0.34	0.87	0.93	1.77	2.04	1.81	1.97	1.00	1.25	1.47	0.97	0.95	0.77	1.11
Smithella	0.20	0.48	0.71	0.75	1.99	1.99	1.46	1.94	0.80	0.56	1.18	0.86	0.72	0.69	0.65
Clostridium	0.14	0.34	0.86	0.61	1.54	1.70	1.57	1.74	0.76	0.84	0.82	1.43	0.99	0.98	1.09
Geobacter	0.07	0.13	0.74	0.82	1.22	0.81	0.90	1.03	1.17	0.83	1.06	0.63	0.97	0.87	1.00
Lactobacillus	0.27	0.26	0.51	0.52	1.07	1.33	0.83	0.94	0.64	0.37	0.73	0.56	0.50	0.55	0.52
Bacteriovorax	0.06	0.17	0.60	0.58	1.00	0.94	0.71	0.72	0.55	0.46	0.68	0.44	0.64	0.63	0.73
Sphingomonas	0.05	0.16	0.35	0.41	0.46	0.99	0.73	1.26	0.46	0.61	0.62	0.62	0.35	0.37	0.42
Halospirulina	0.05	0.17	0.61	0.43	1.07	1.03	1.09	1.47	0.88	0.97	0.69	1.27	0.44	0.56	0.54
Cyanothece	0.09	0.26	0.38	0.27	0.91	1.12	1.43	1.61	0.33	1.38	0.88	0.93	0.41	0.53	0.63
Flavobacterium	0.03	0.07	0.54	0.45	0.74	0.85	1.20	1.08	0.53	1.19	0.71	0.85	0.59	0.71	0.68
Cyanobacterium	0.04	0.13	0.00	0.00	0.65	0.94	1.18	1.34	0.00	0.51	0.55	0.65	0.00	0.00	0.33
Chromohalobacter	0.00	0.00	0.50	0.83	0.00	0.00	0.00	0.00	0.64	0.74	0.37	1.17	0.00	0.00	0.00
Shewanella	0.00	0.00	0.44	0.56	0.00	0.00	0.00	0.00	1.23	0.00	0.00	0.78	0.35	0.32	0.31

Relative abundance (%): 30, 20, 10, 0

Supplementary Fig. 31: Relative abundance of the bacterial taxa and their hierarchical clustering in the significant hotspots in axenically-grown and outdoor-grown leaves. 'P' - plant, 'L' - leaf.

Outdoor and axenically-grown leaves - fungi

Taxon	P1.L2.4	S2.L1	S2.L2	P2.L2.4	P2.L2.3	P1.L1.2	P1.L1.4	P2.L1.1	P2.L1.3	P2.L1.4	P2.L2.2	P2.L2.1	P1.L2.2	P1.L1.1	P1.L2.1
Phaeosphaeria	4.96	0.00	0.00	0.33	28.60	26.44	24.04	16.32	19.41	20.21	19.98	18.39	17.88	18.59	19.48
Rhizophagus	4.50	5.85	9.48	22.90	17.31	13.59	15.33	19.56	17.30	16.44	19.70	19.36	14.65	15.77	13.33
Chloromonas	4.76	3.03	6.12	16.73	12.16	11.12	13.76	16.86	15.08	15.17	15.78	14.93	12.97	14.53	12.60
Dictyochloris	66.46	0.00	0.00	0.00	0.00	0.00	0.00	0.00	0.01	0.00	0.00	0.00	2.04	0.00	0.00
Amanita	1.10	1.52	2.75	7.44	4.48	7.00	6.10	7.58	7.26	7.43	6.76	7.58	4.92	7.48	6.39
Femsjonia	1.08	1.95	2.60	8.97	4.34	5.02	4.45	5.06	6.21	6.08	4.64	5.72	4.77	4.15	6.51
Elaphomyces	0.93	2.45	2.29	7.24	4.69	4.61	4.74	5.83	5.74	5.92	5.30	6.05	5.12	4.40	4.14
Other (<=1%)	0.99	11.77	7.34	4.67	4.42	3.62	3.57	4.15	3.61	3.63	3.65	3.77	4.42	3.80	6.03
Roccella	0.71	0.87	1.99	4.41	2.12	3.79	3.37	4.27	4.24	4.18	3.79	4.42	2.63	4.49	2.74
Helminthosporium	0.85	1.16	0.31	3.50	1.74	2.47	3.29	3.38	3.27	3.62	3.34	3.81	2.48	2.91	3.16
Marasmius	0.48	0.65	1.38	3.67	2.25	2.14	2.45	3.01	2.73	2.46	2.84	2.89	1.94	3.80	2.37
Geopora	0.42	0.00	0.46	3.22	1.38	1.57	1.93	2.66	2.28	2.71	2.65	2.33	1.89	2.22	2.56
Elmerina	0.48	0.58	1.22	3.16	1.63	1.48	1.44	2.32	2.35	1.76	2.71	2.36	1.54	2.18	2.19
Plasmodium	0.65	7.51	3.98	0.70	3.12	2.88	1.97	0.50	0.90	0.65	0.51	0.28	4.02	1.32	2.07
Aspergillus	0.28	2.53	3.98	0.45	1.93	1.81	1.32	0.44	0.70	0.63	0.33	0.20	1.79	1.15	1.46
Saccharomyces	0.17	3.97	3.36	0.80	0.92	1.32	1.00	0.34	0.45	0.58	0.34	0.38	1.34	0.64	0.73
Strongyloides	0.25	2.82	1.99	0.23	1.30	2.06	0.84	0.27	0.35	0.43	0.30	0.16	1.69	0.77	1.28
Gregarina	0.00	4.91	5.81	0.00	0.00	0.00	0.00	0.00	0.00	0.00	0.00	0.00	0.00	0.00	0.00
Clavispora	0.00	3.61	2.91	0.10	0.19	0.58	0.16	0.05	0.15	0.08	0.00	0.00	0.15	0.04	0.24
Chlamydomonas	0.48	2.02	4.43	0.11	0.00	0.00	0.00	0.00	0.09	0.00	0.11	0.00	0.45	0.04	0.12
Willeya	0.25	0.00	0.00	2.66	0.95	1.73	1.73	1.72	1.90	2.03	1.64	1.94	1.99	2.18	1.64
Phlyctochytrium	0.34	0.00	0.00	1.62	1.06	1.73	1.77	1.40	1.57	1.58	1.29	1.20	0.94	1.28	1.58
Talaromyces	0.23	0.00	0.46	1.55	0.84	0.82	1.40	1.09	1.29	1.03	1.18	1.21	0.65	0.98	1.22
Phellinus	0.17	0.00	0.31	1.41	0.62	0.91	1.08	1.00	0.81	0.81	1.16	0.97	0.89	1.20	1.64
Diplosphaera	0.23	0.00	0.31	1.15	1.00	0.41	0.92	1.13	0.90	0.90	0.94	0.89	1.09	0.51	0.79
Pyropia	0.23	0.00	0.61	0.00	0.00	0.74	0.96	0.00	0.00	0.00	0.00	0.00	0.00	2.95	0.37
Trichophyton	0.08	0.00	0.00	1.10	0.35	0.16	0.40	0.44	0.46	0.57	0.45	0.55	0.60	0.64	0.79
Phyllozyma	0.06	0.00	0.00	1.27	0.38	0.33	0.36	0.24	0.16	0.21	0.23	0.28	0.65	0.51	1.40
Crassostrea	0.03	1.23	0.46	0.00	0.35	0.16	0.16	0.00	0.08	0.06	0.00	0.00	0.55	0.17	0.24
Berthella	0.00	1.01	0.31	0.00	0.30	0.33	0.12	0.00	0.00	0.00	0.00	0.00	0.45	0.09	0.61
Acropora	0.00	1.16	0.31	0.15	0.16	0.25	0.00	0.09	0.09	0.22	0.06	0.14	0.15	0.00	0.37
Mychonastes	1.25	0.00	0.00	0.00	0.00	0.00	0.00	0.00	0.00	0.00	0.00	0.00	0.00	0.00	0.00
Albugo	0.03	2.60	1.83	0.12	0.27	0.25	0.32	0.01	0.14	0.10	0.04	0.00	0.40	0.21	0.55
Compsopogon	0.00	1.81	1.38	0.00	0.24	0.33	0.28	0.05	0.11	0.10	0.00	0.00	0.15	0.00	0.43
Tritia	0.00	2.67	0.76	0.00	0.00	0.00	0.00	0.00	0.00	0.00	0.00	0.00	0.00	0.00	0.00
Toxoplasma	0.00	2.02	0.76	0.00	0.00	0.00	0.00	0.00	0.00	0.00	0.00	0.00	0.00	0.00	0.00
Chlorella	0.06	1.23	2.29	0.00	0.05	0.16	0.08	0.00	0.08	0.00	0.00	0.00	0.40	0.04	0.12
Pyramimonas	0.00	1.73	2.14	0.00	0.00	0.00	0.00	0.00	0.00	0.00	0.00	0.00	0.00	0.00	0.00
Komagataella	0.00	0.79	1.22	0.33	0.43	0.00	0.40	0.19	0.21	0.22	0.21	0.18	0.30	0.34	0.61
Pecten	0.06	1.08	1.22	0.00	0.33	0.16	0.04	0.00	0.00	0.06	0.00	0.00	0.65	0.13	0.24
Ostreobium	0.00	0.51	1.83	0.00	0.00	0.00	0.20	0.00	0.00	0.00	0.00	0.00	0.00	0.47	0.00
Multicilia	0.00	0.94	1.38	0.00	0.00	0.00	0.00	0.00	0.00	0.00	0.00	0.00	0.00	0.00	0.00
Chlorococcum	0.00	0.00	0.00	0.00	0.05	0.00	0.00	0.06	0.09	0.10	0.07	0.00	3.48	0.00	0.00
Ankyra	3.80	0.00	0.00	0.00	0.00	0.00	0.00	0.00	0.00	0.00	0.00	0.00	0.00	0.00	0.00
Spermatozopsis	3.63	0.00	0.00	0.00	0.00	0.00	0.00	0.00	0.00	0.00	0.00	0.00	0.00	0.00	0.00
Peltigera	0.00	24.04	20.03	0.00	0.00	0.00	0.00	0.00	0.00	0.00	0.00	0.00	0.00	0.00	0.00

Relative abundance (%): 30, 20, 10, 0

Supplementary Fig. 32: Relative abundance of the fungal taxa and their hierarchical clustering in the significant hotspots in axenically-grown and outdoor-grown leaves. ‘P’ - plant, ‘L’ - leaf.

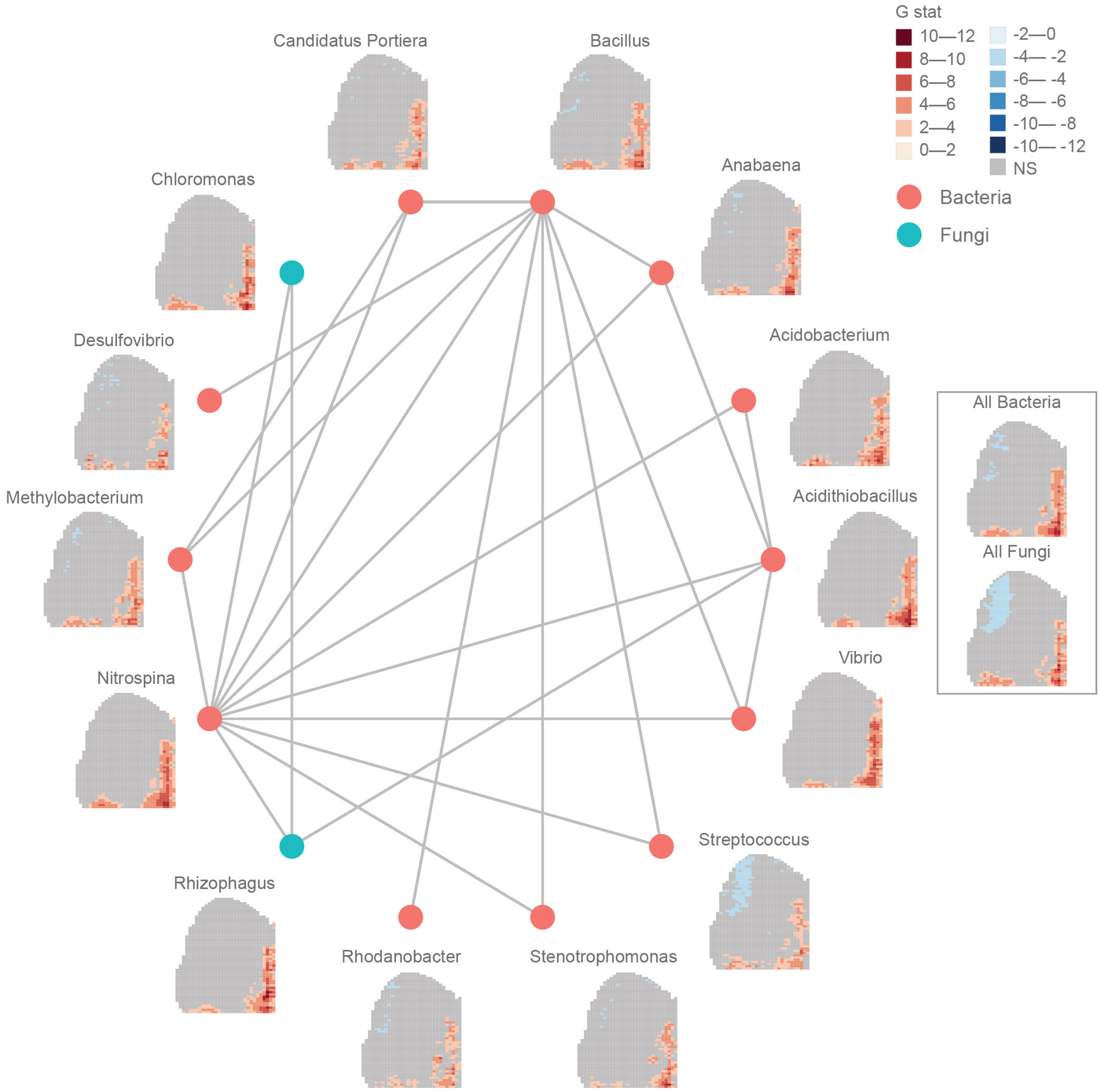

Supplementary Fig. 33: Subnetwork of 14 bacterial and fungal taxa strongly associated with microbial abundance in all leaf sections analysed. An edge connects two taxa if their average pairwise SRCC, across all sections, is above or equal to 0.35. The hotspot pattern for each genus in a representative leaf section (P2.L1.4) is shown next to the network nodes.

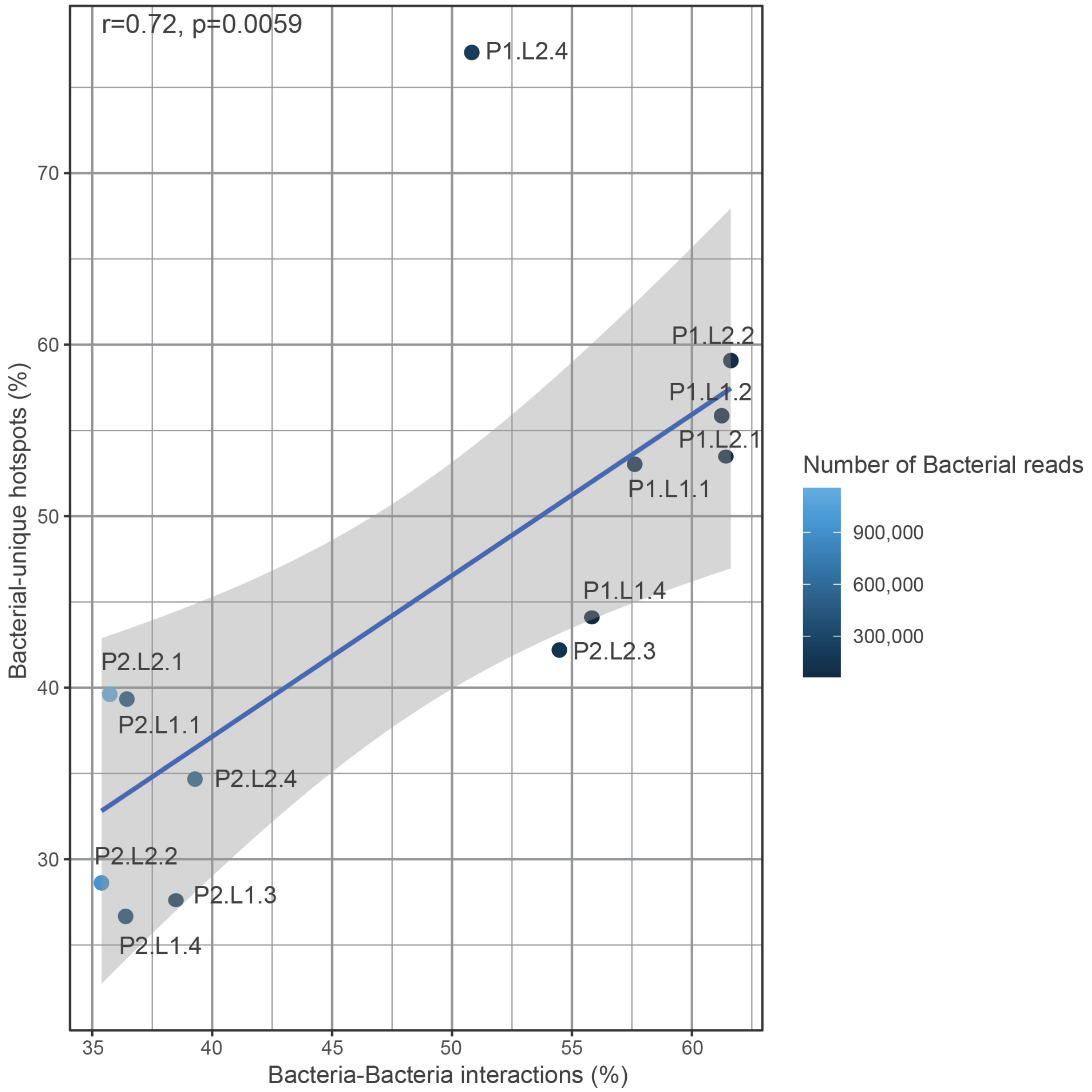

Supplementary Fig. 34: The proportion of bacteria-bacteria interactions as a function of the proportion of bacterial-unique hotspots. Spearman's rank correlation coefficient ρ=0.72, p-value=0.059 (two-tailed test).

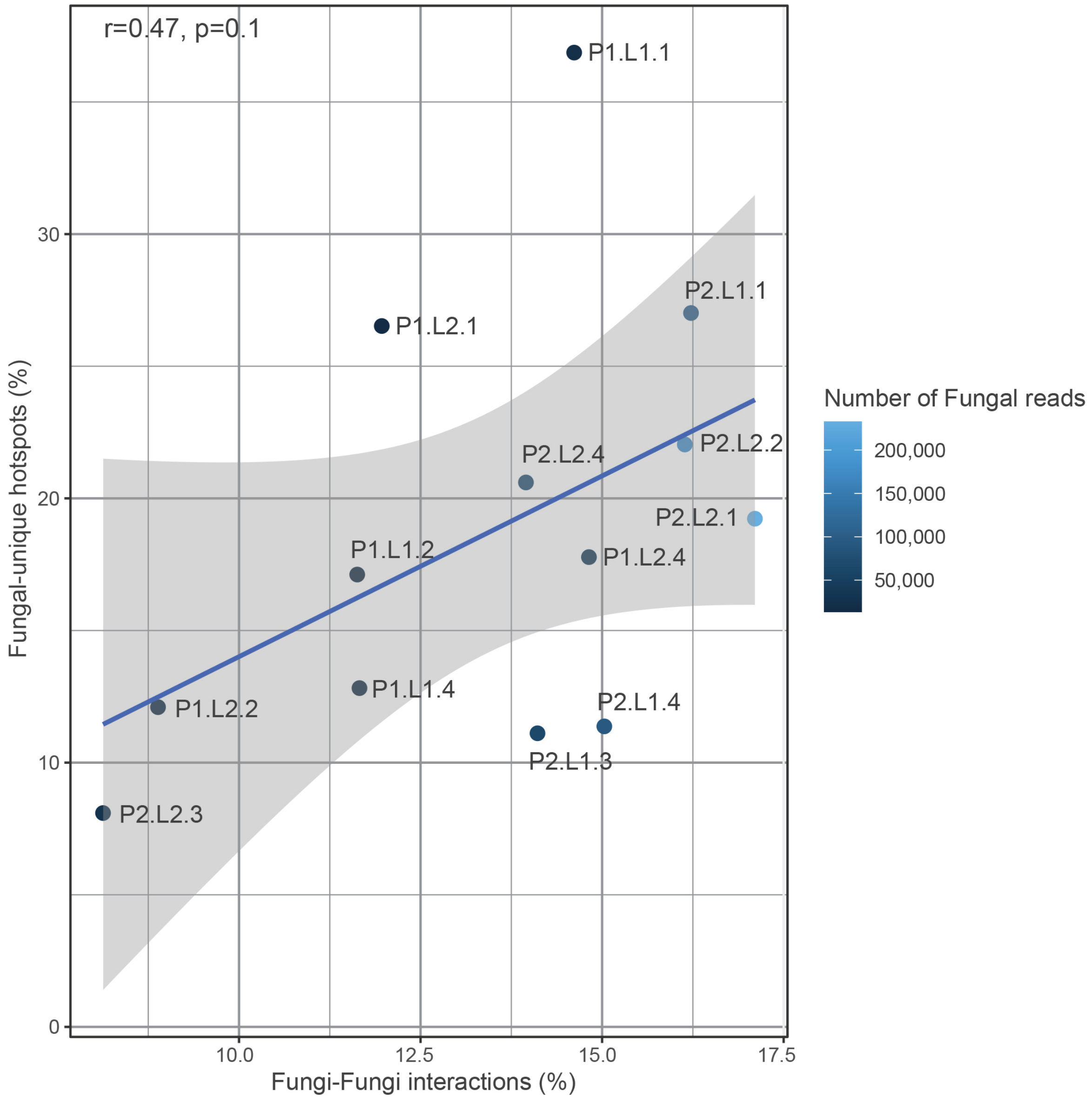

Supplementary Fig. 35: The proportion of fungi-fungi interactions as a function of the proportion of fungi-unique hotspots. Spearman's rank correlation coefficient ρ=0.47, p-value=0.1 (two-tailed test).

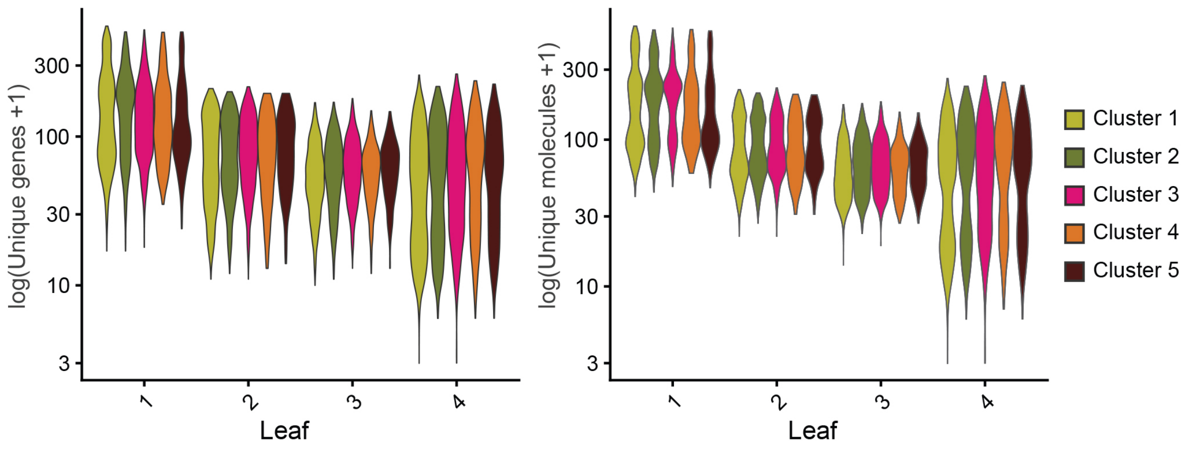

Supplementary Fig. 36: Normalised unique genes and unique molecules for each host gene expression unsupervised cluster in each leaf.

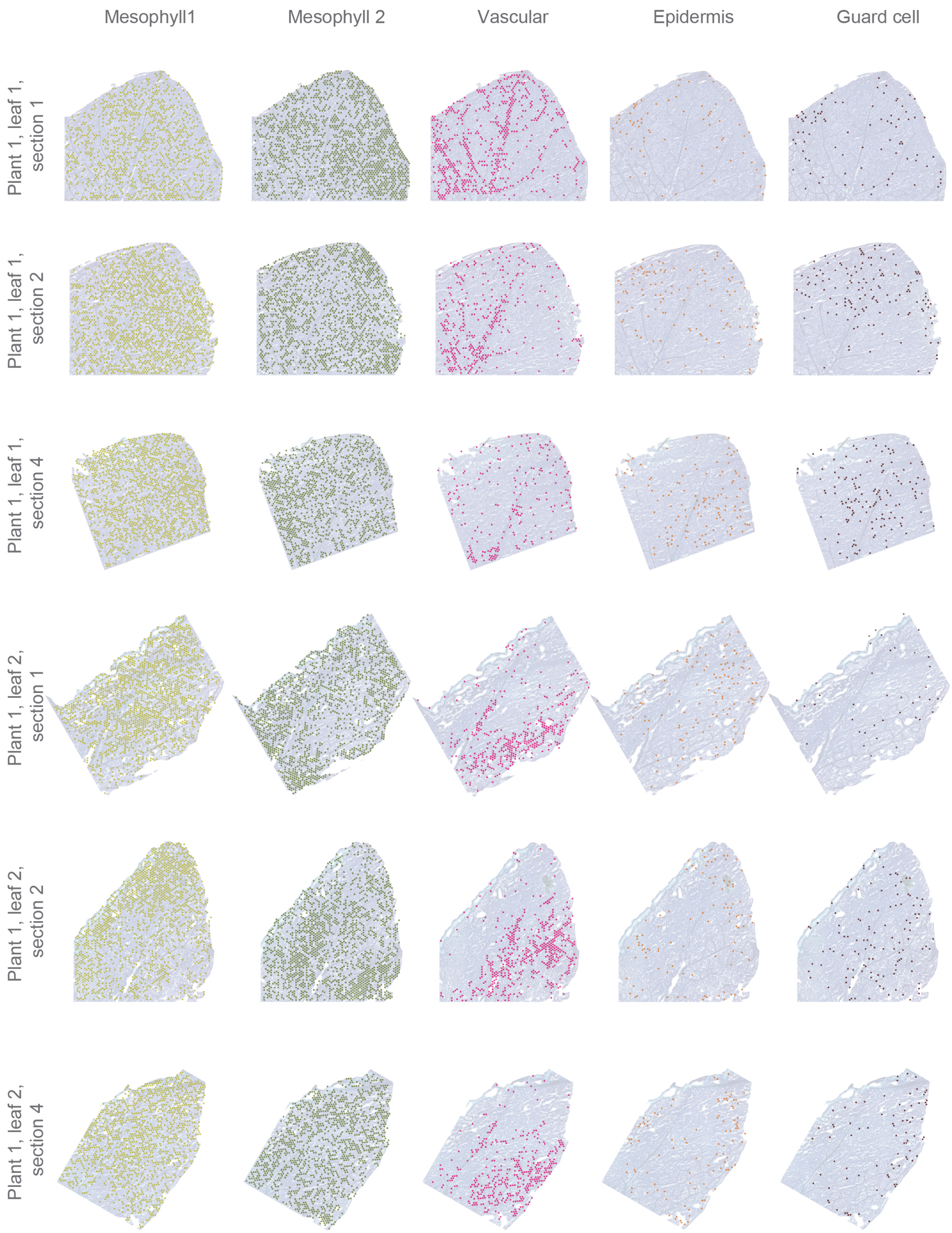

Mesophyll1
Mesophyll 2
Vascular
Epidermis
Guard cell
Plant 1, leaf 1, section 1
Plant 1, leaf 1, section 2
Plant 1, leaf 1, section 4
Plant 1, leaf 2, section 1
Plant 1, leaf 2, section 2
Plant 1, leaf 2, section 4

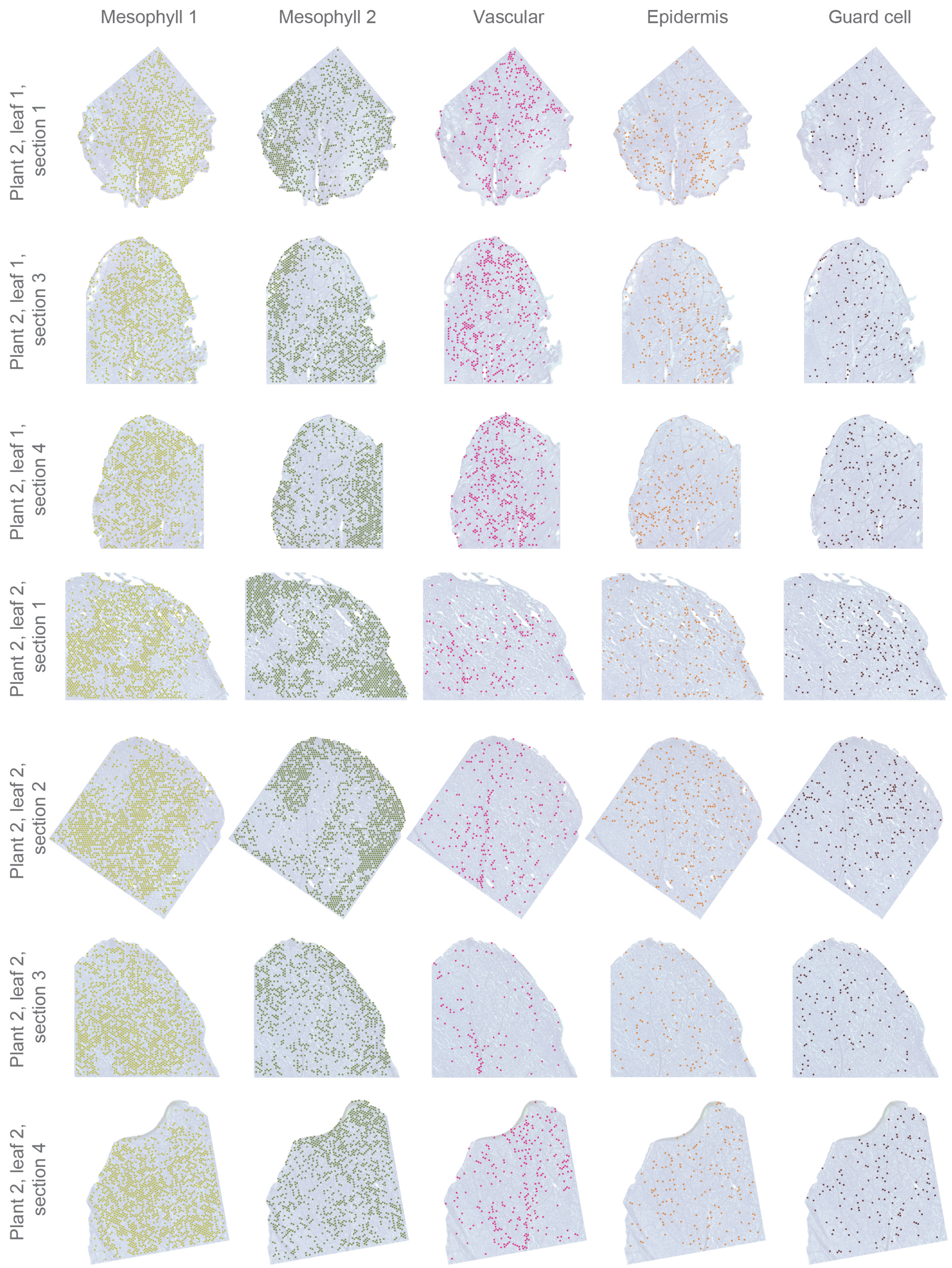

Supplementary Fig. 37: All five UMAP clusters individually visualised in each tissue section.

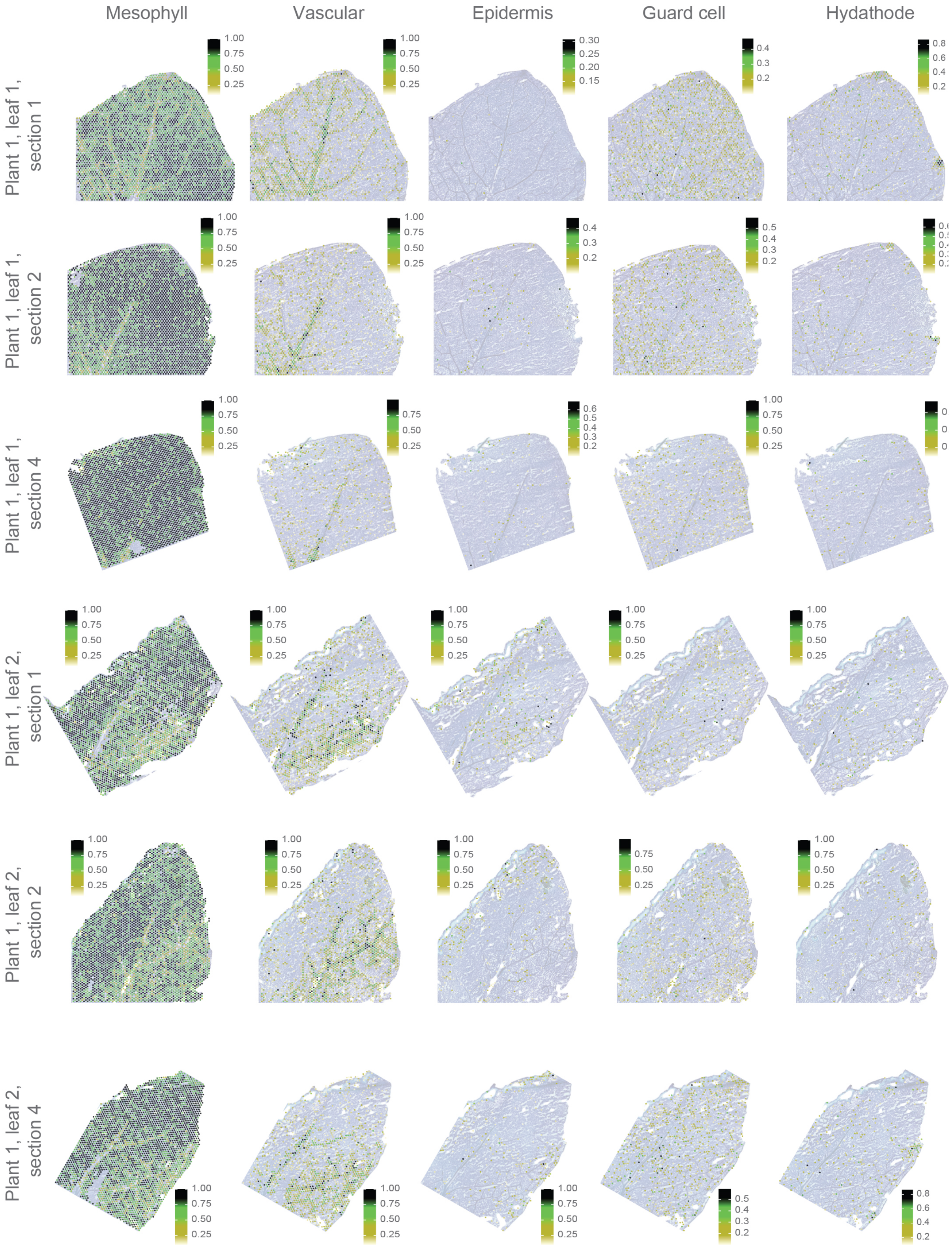
Mesophyll
Vascular
Epidermis
Guard cell
Hydathode
Plant 1, leaf 1, section 1
Plant 1, leaf 1, section 2
Plant 1, leaf 1, section 4
Plant 1, leaf 2, section 1
Plant 1, leaf 2, section 2
Plant 1, leaf 2, section 4

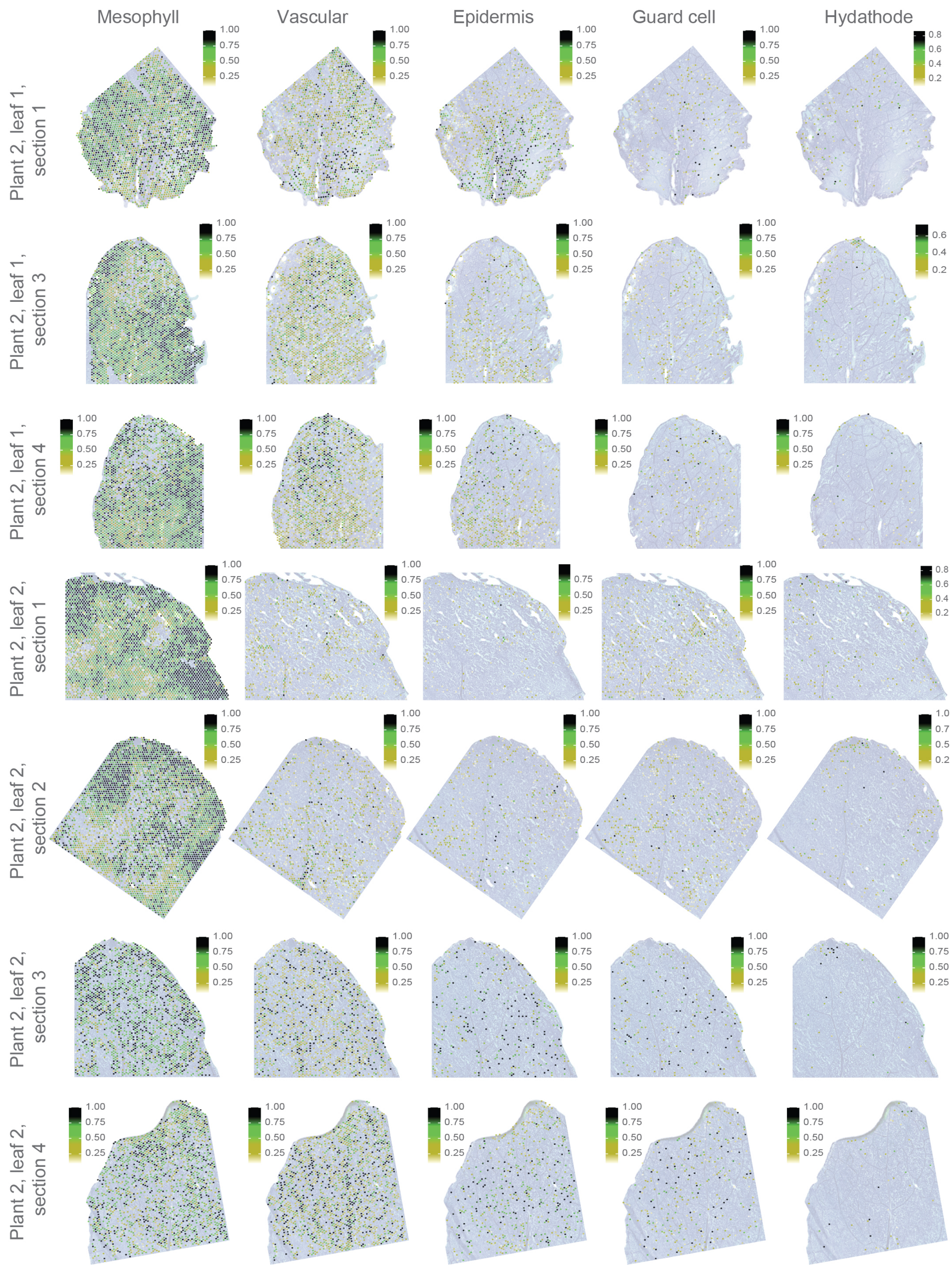

Supplementary Fig. 38: Single-cell proportions of the merged main cell types projected on each of the tissue sections. To improve the visualisation the values presented here are scaled quantiles (see **Methods**), and only spots with a cell type proportion greater than zero are displayed.

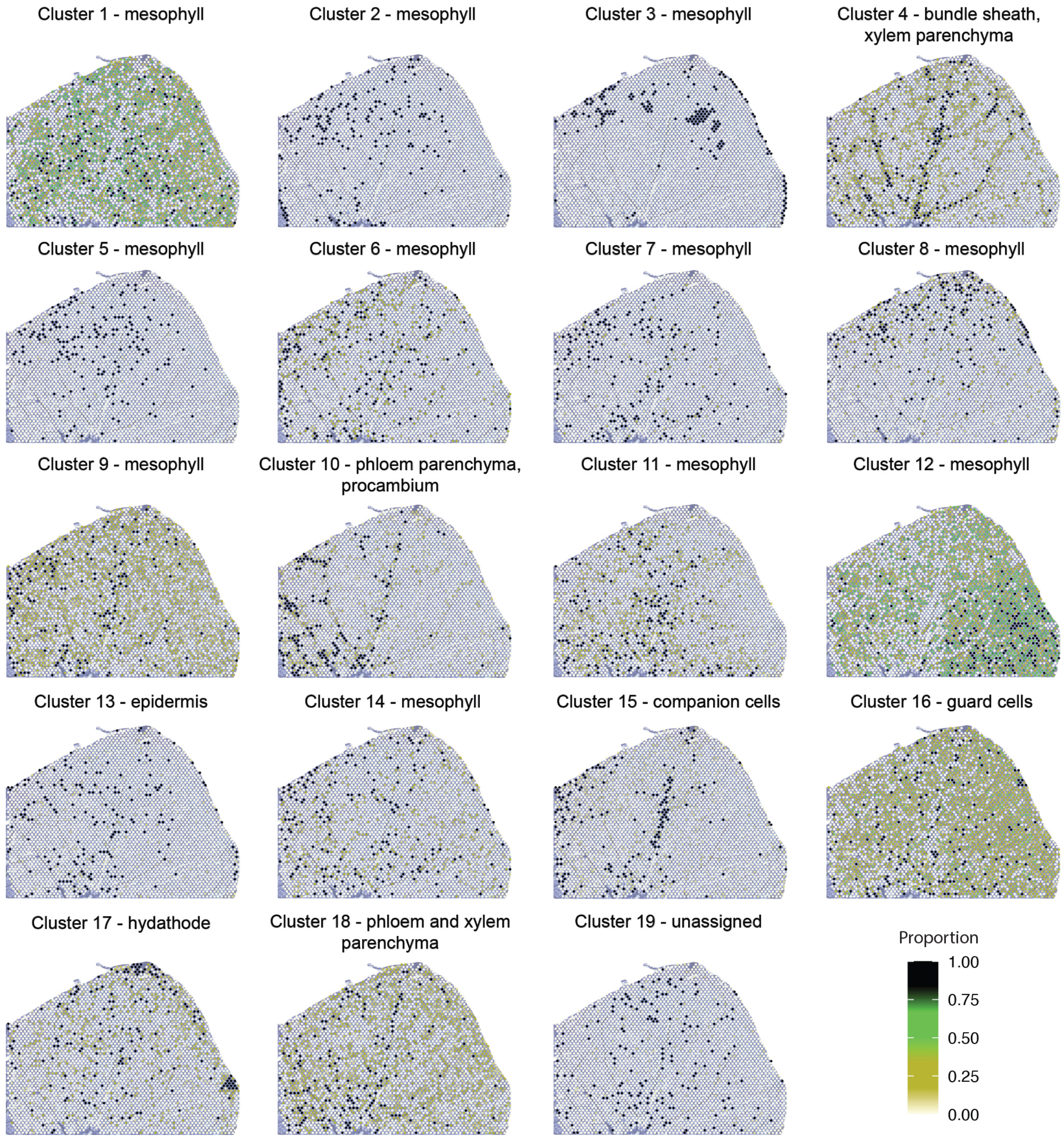
Cluster 1 - mesophyll
Cluster 2 - mesophyll
Cluster 3 - mesophyll
Cluster 4 - bundle sheath, xylem parenchyma
Cluster 5 - mesophyll
Cluster 6 - mesophyll
Cluster 7 - mesophyll
Cluster 8 - mesophyll
Cluster 9 - mesophyll
Cluster 10 - phloem parenchyma, procambium
Cluster 11 - mesophyll
Cluster 12 - mesophyll
Cluster 13 - epidermis
Cluster 14 - mesophyll
Cluster 15 - companion cells
Cluster 16 - guard cells
Cluster 17 - hydathode
Cluster 18 - phloem and xylem parenchyma
Cluster 19 - unassigned
Proportion
1.00
0.75
0.50
0.25
0.00

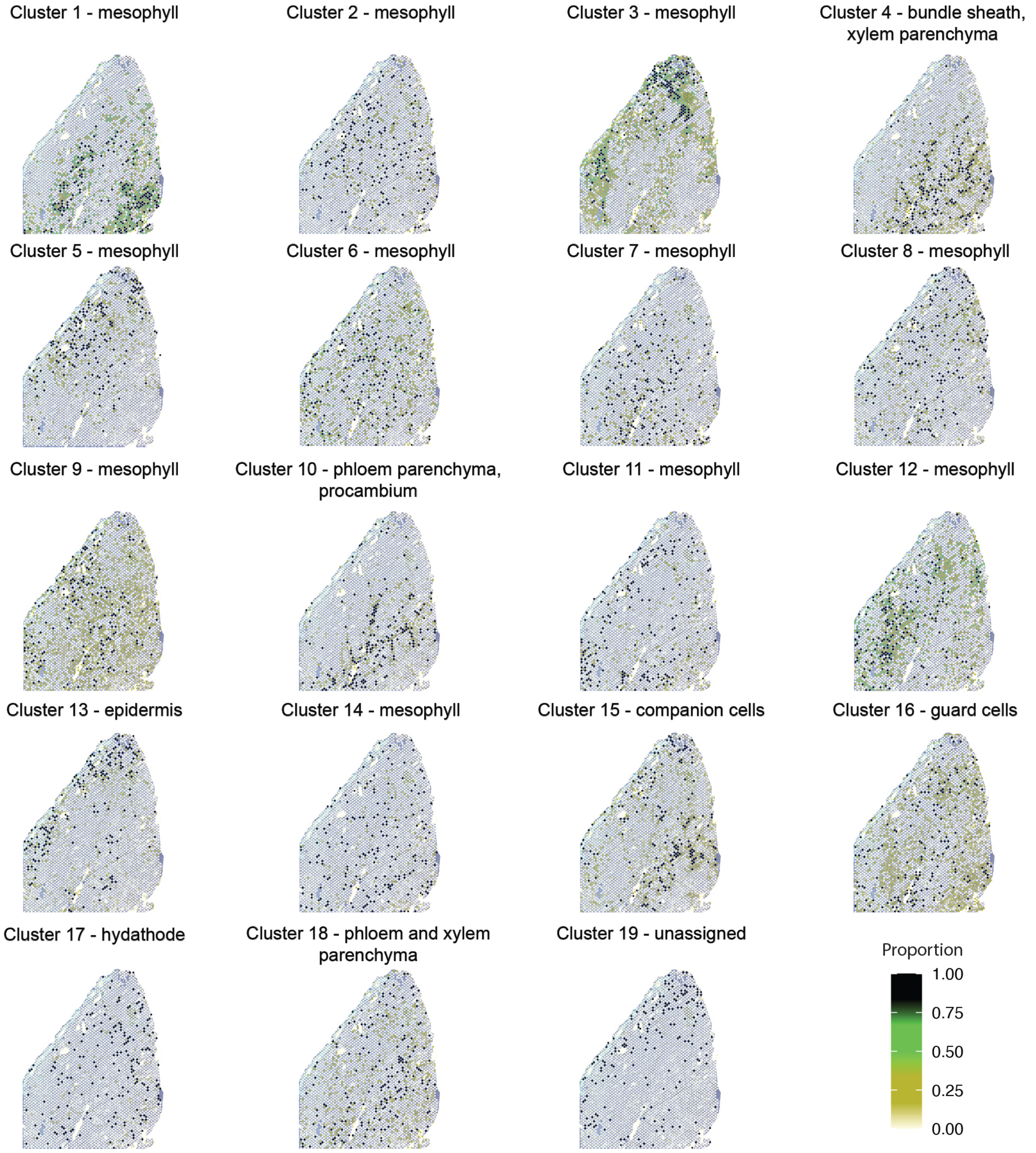

Supplementary Fig. 39: Single-cell proportions of all the cell types projected on two representative tissue sections. To improve the visualisation the values presented here are scaled quantiles (see **Methods**), and only spots with a cell type proportion greater than zero are displayed.

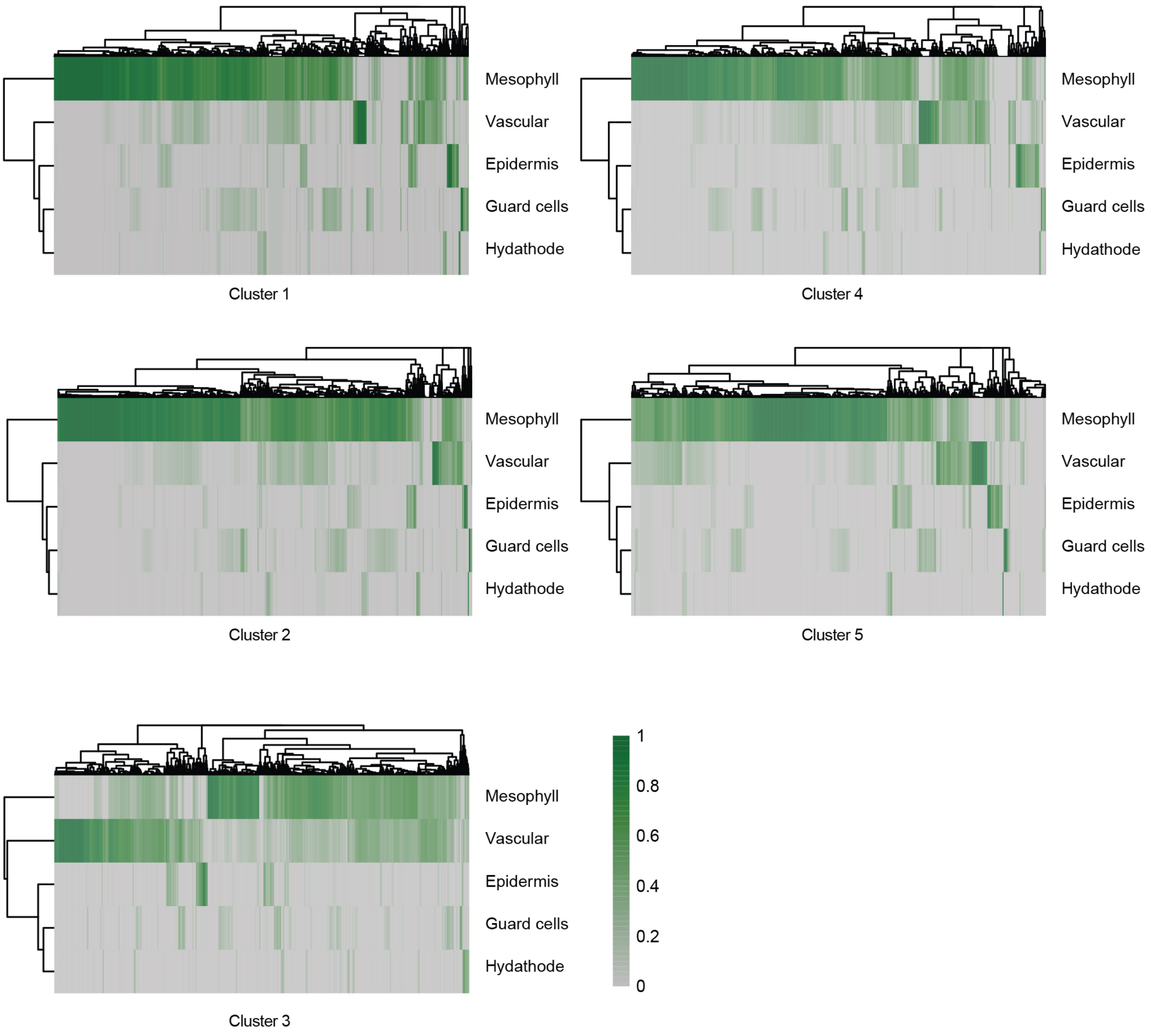

Supplementary Fig. 40: Estimated cell type proportion values from 0-1 for each of the clusters and hierarchical clustering for each spot and cell type.

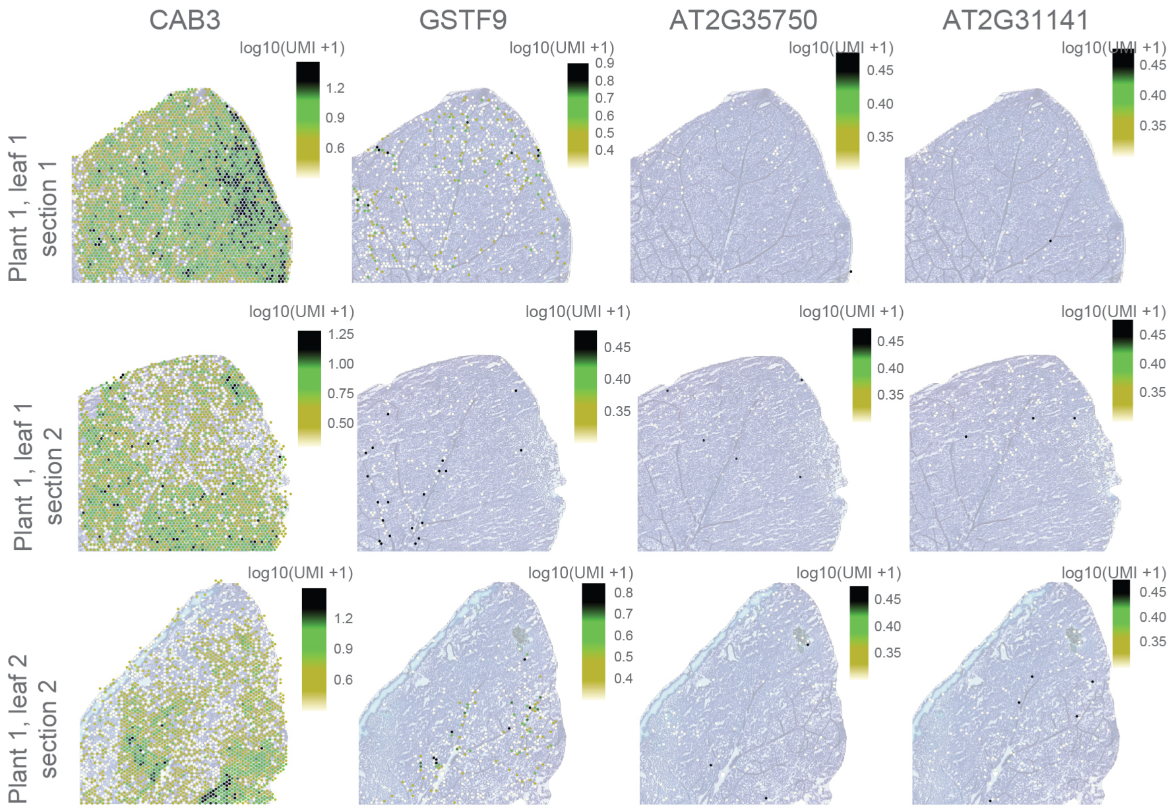

Supplementary Fig. 41: Representative markers for some of the clusters for three representative sections. *CAB3*: CHLOROPHYLL A/B BINDING PROTEIN 3 (Cluster 2 marker), *GSTF9*: GLUTATHIONE S-TRANSFERASE PHI 9 (Cluster 3 marker), AT2G35750: transmembrane protein (Cluster 4 marker), AT2G31141: potential nitrate responsive gene (Cluster 5 marker).

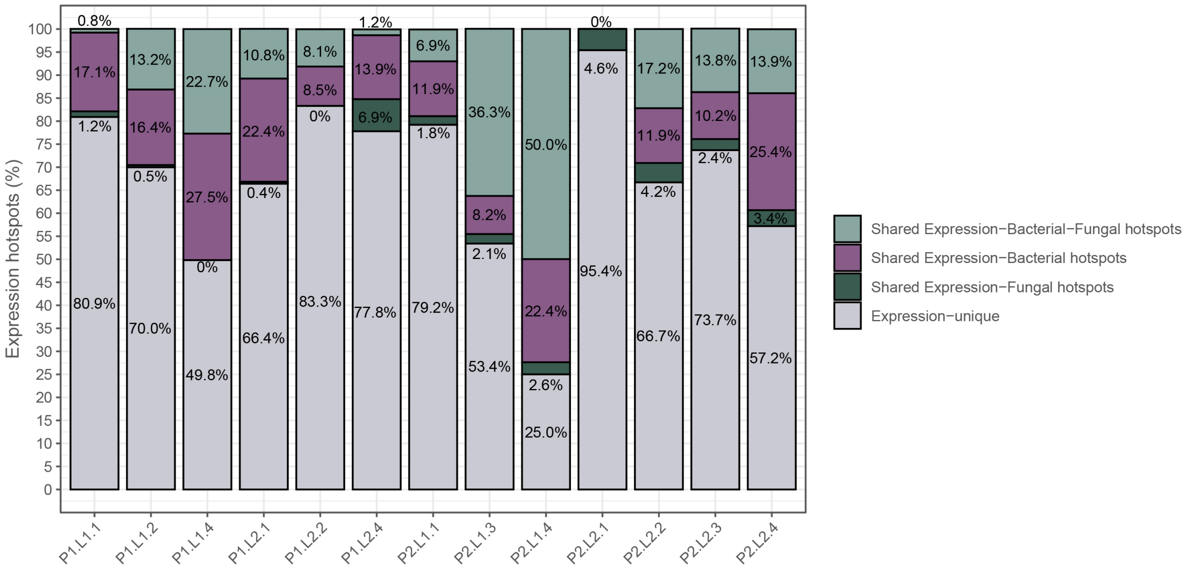

Supplementary Fig. 42: Proportion of hotspots shared between the host *A. thaliana*, bacterial taxa, and fungal taxa in each of the sections.

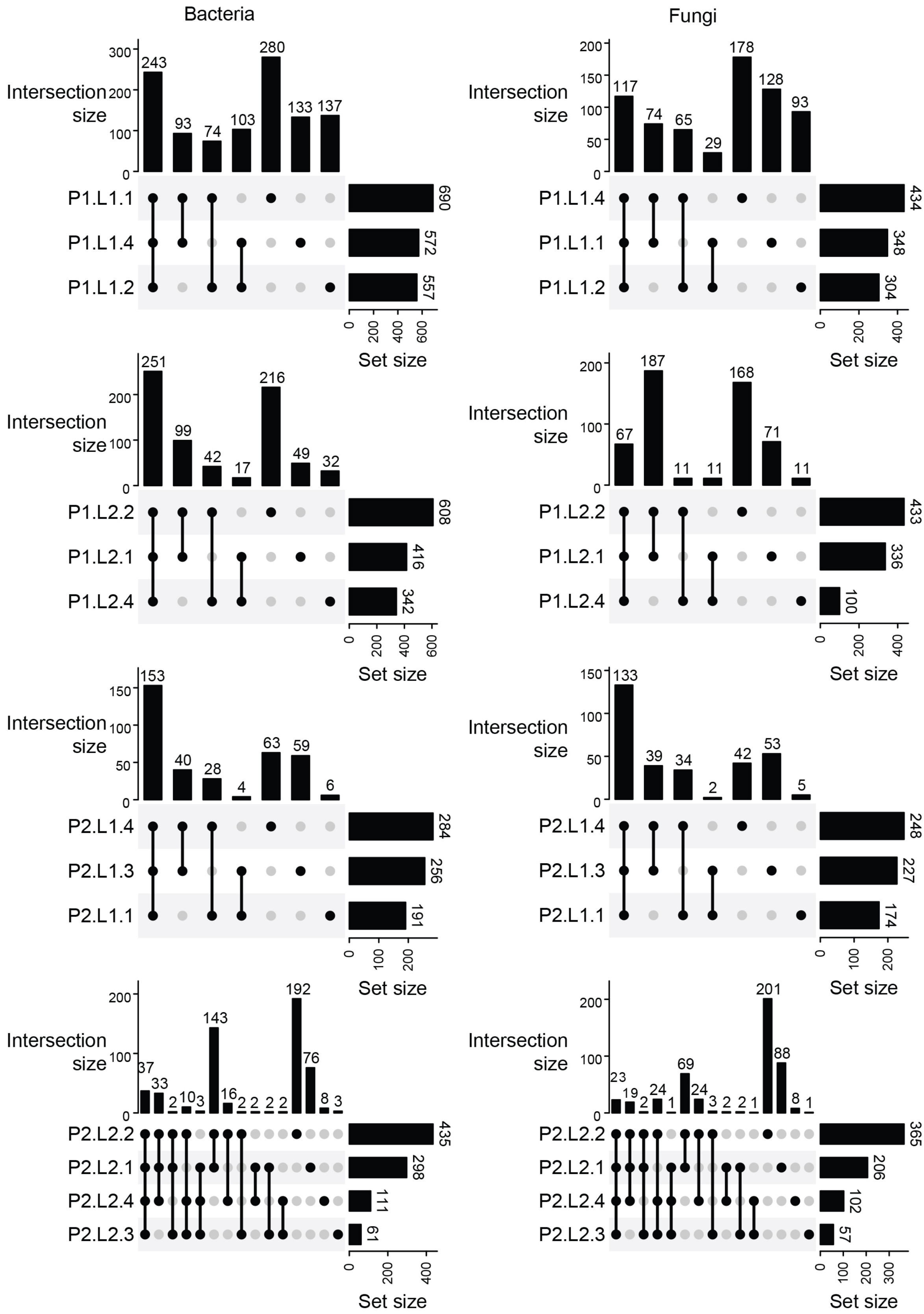

Supplementary Fig. 43: Set of genes selected by Boruta as explanatory of the total bacterial or fungal abundance in a given leaf section were intersected across the leaf sections. Intersection size of the different groups are shown.

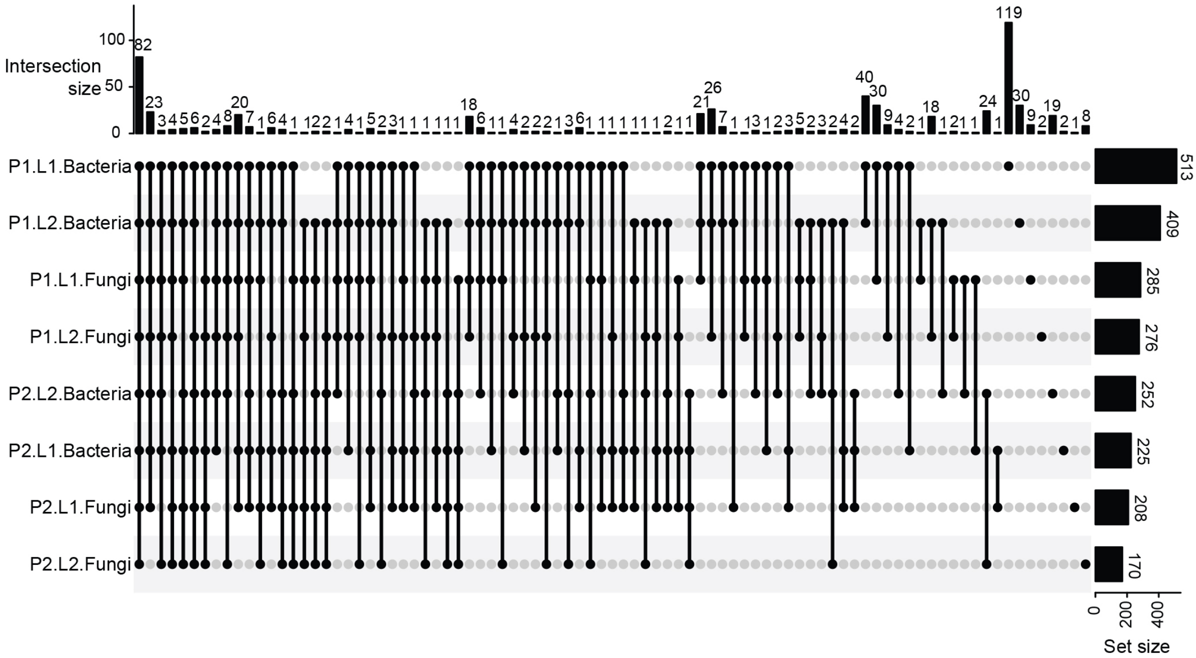

Supplementary Fig. 44: Intersection of bacterial and fungal associated genes across different leaves. Most of the genes are common to bacteria and fungi.

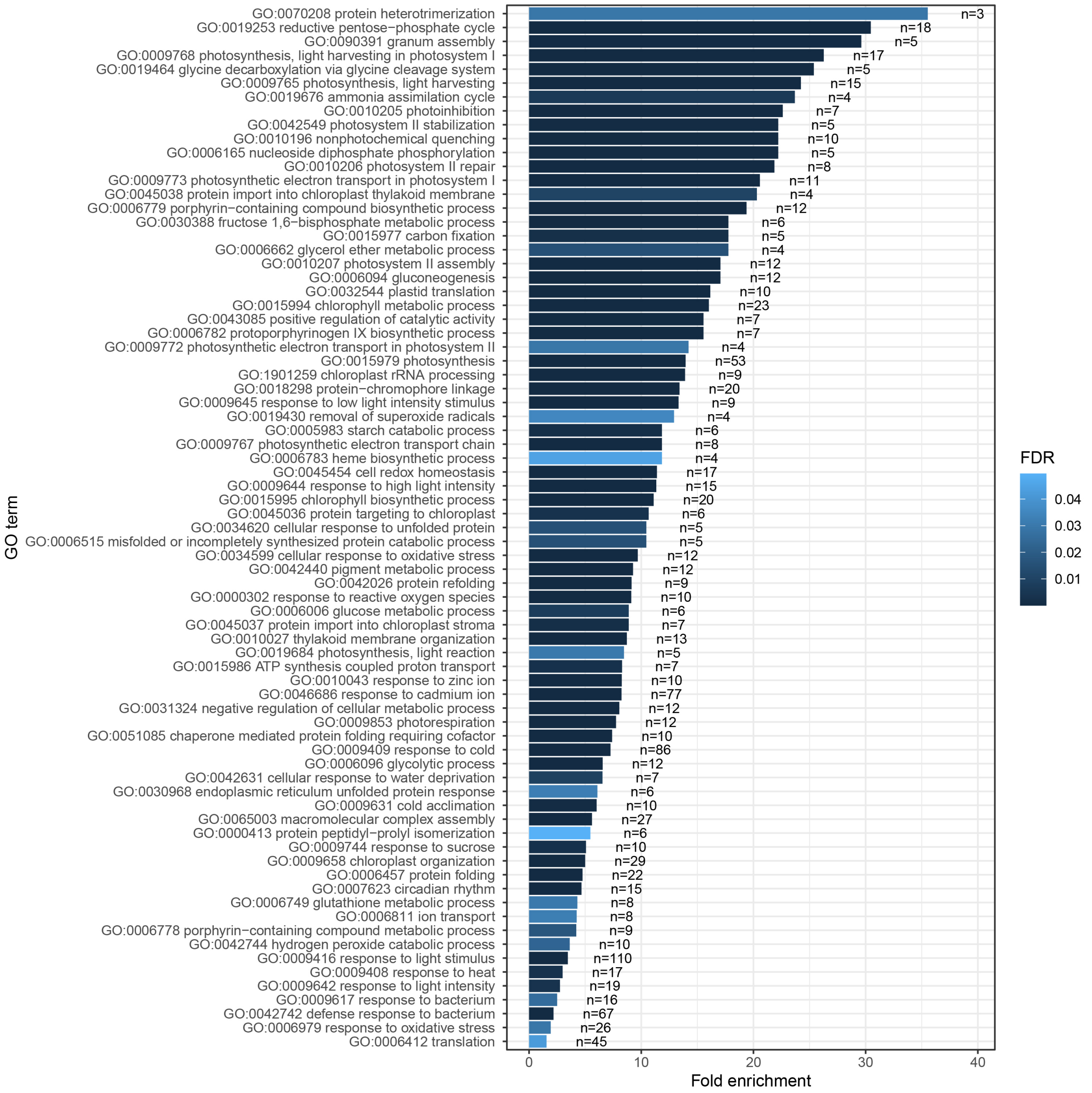

Supplementary Fig. 45: Overrepresented GO terms of all genes significantly associated with microbial abundance (n=the number of genes assigned with a specific GO term).

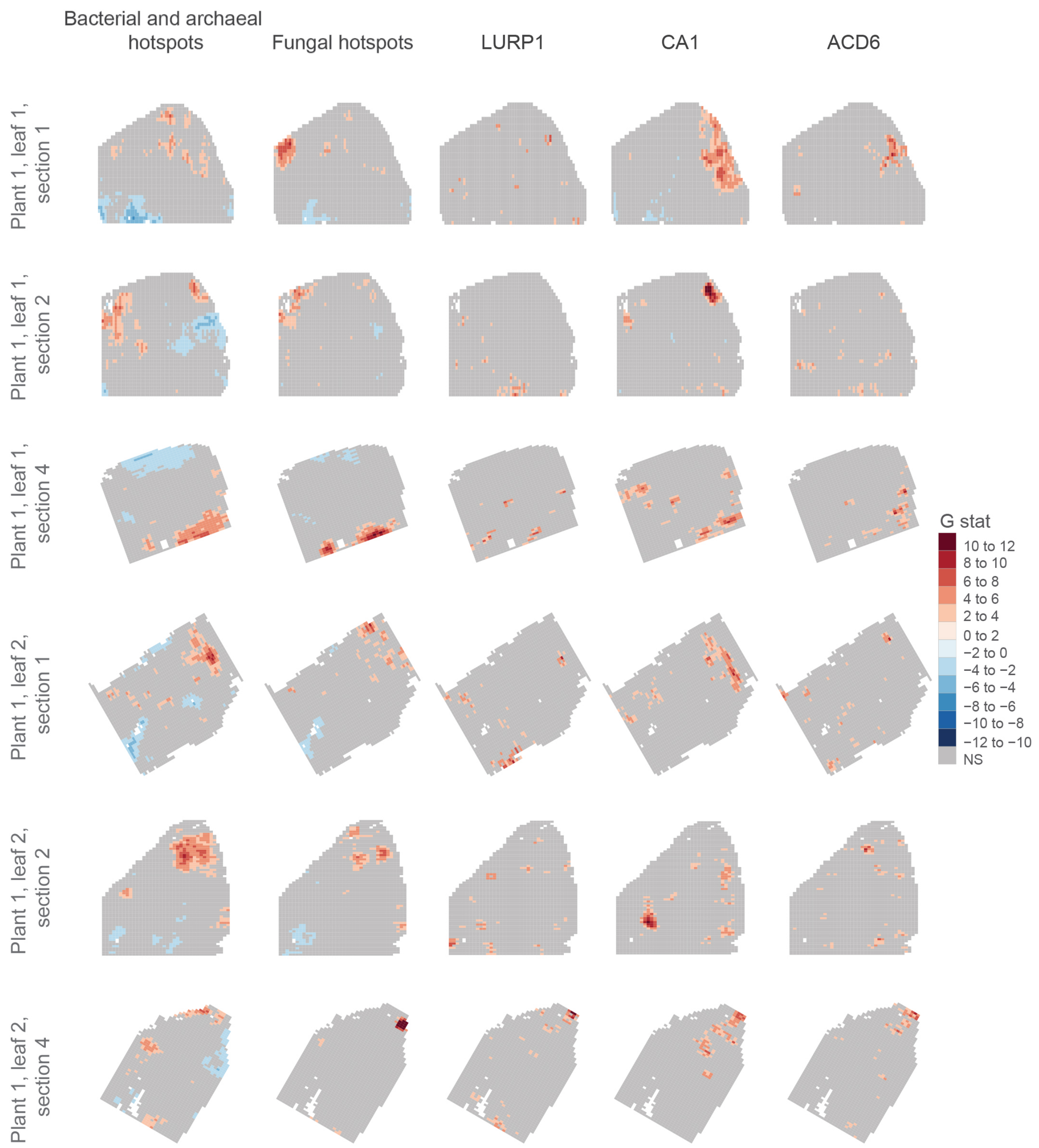
Bacterial and archaeal hotspots
Fungal hotspots
LURP1
CA1
ACD6
Plant 1, leaf 1, section 1
Plant 1, leaf 1, section 2
Plant 1, leaf 1, section 4
Plant 1, leaf 2, section 1
Plant 1, leaf 2, section 2
Plant 1, leaf 2, section 4
G stat
10 to 12
8 to 10
6 to 8
4 to 6
2 to 4
0 to 2
−2 to 0
−4 to −2
−6 to −4
−8 to −6
−10 to −8
−12 to −10
NS

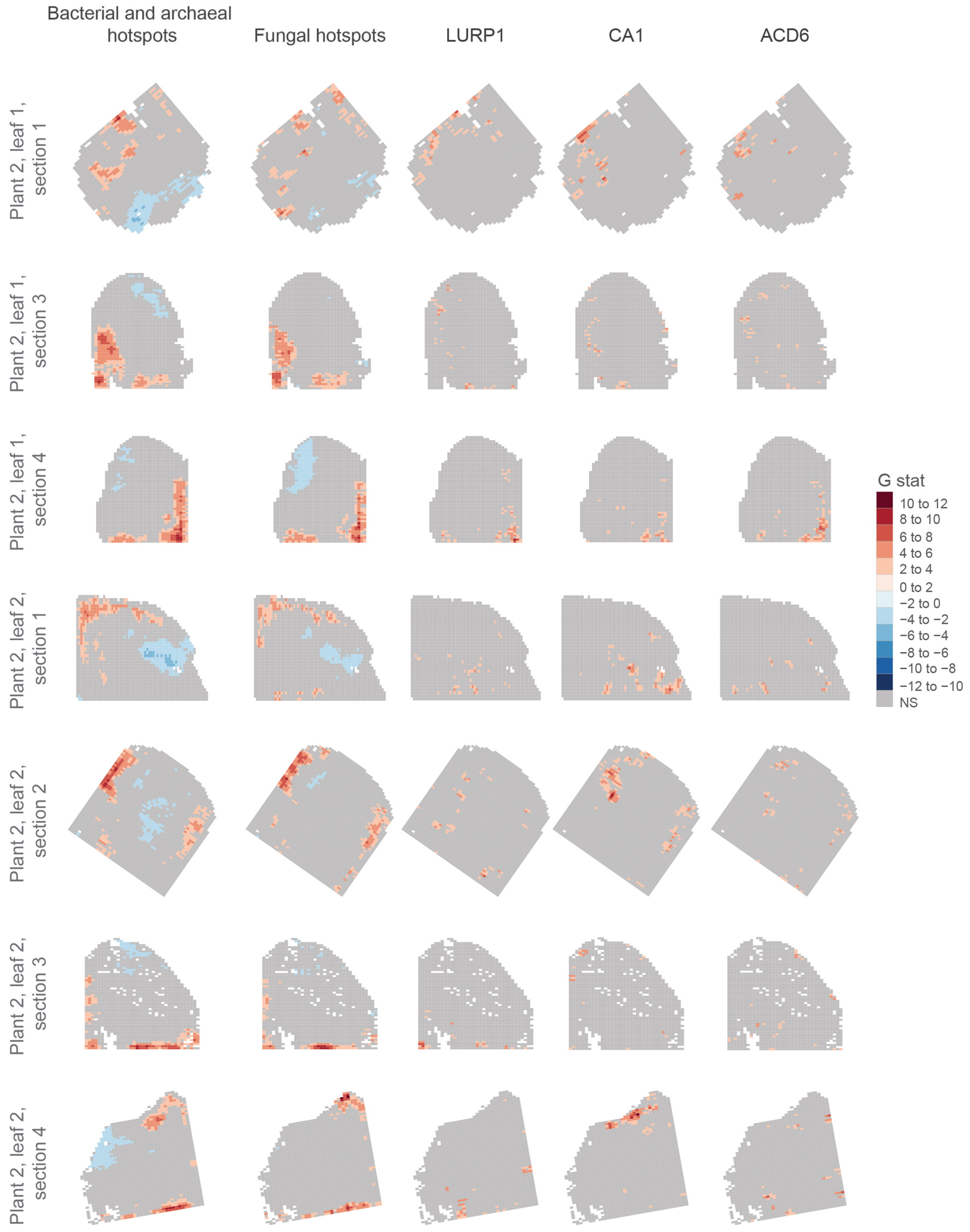

Supplementary Fig. 46: Spatial distribution of significant hotspots of bacteria, fungi, and three defence-related genes: *CA1* (AT3G01500), *LURP1* (AT2G14560), and *ACD6* (AT4G14400).

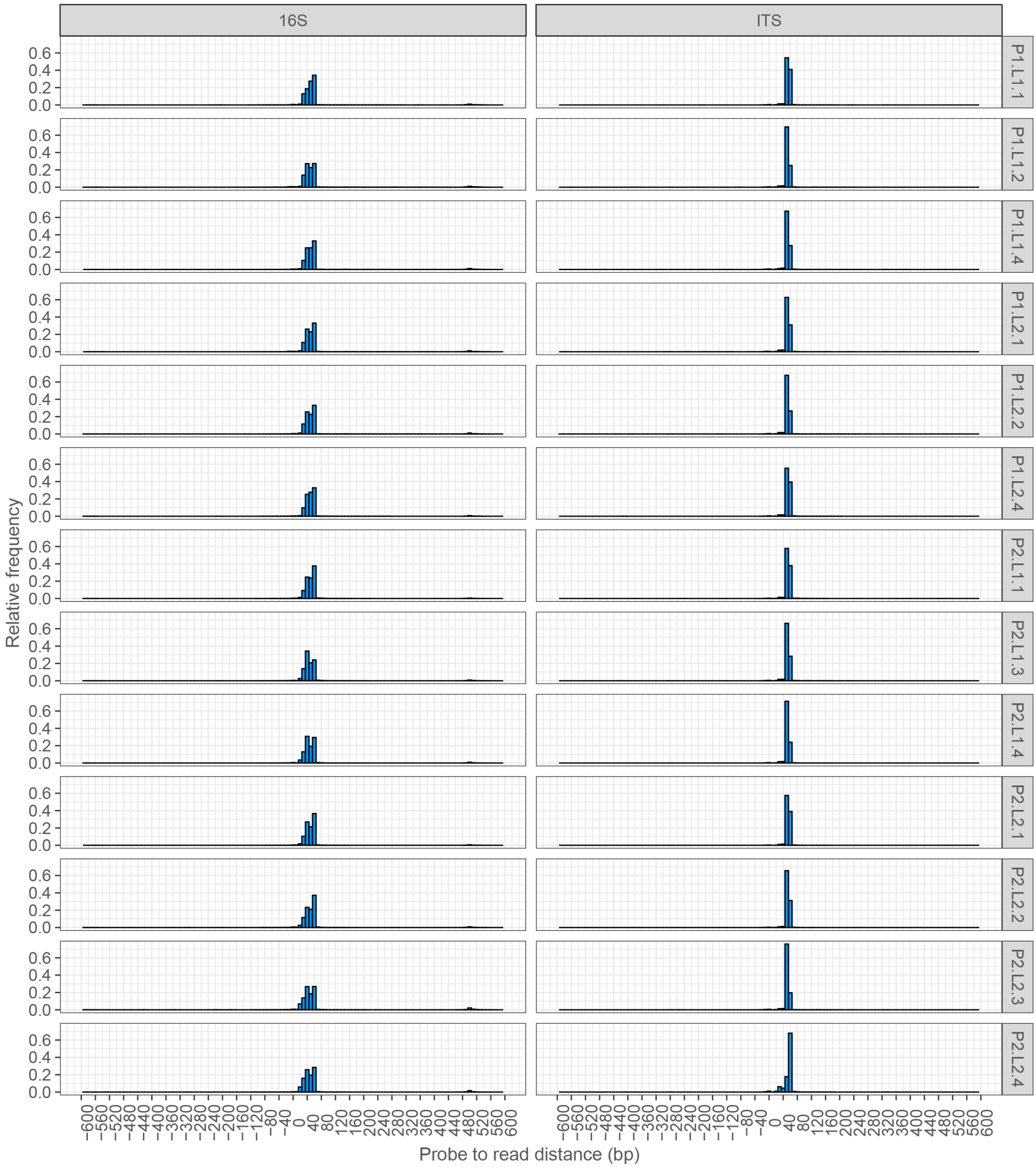

Supplementary Fig. 47: The distribution of distance between reads annotated as microbes to their respective probes (after mapping the reads to NCBI 'nt' database). The result of each section among the different outdoor-grown leaves is presented. 16S analysis comprises the pooled four 16S probes used in this study, while ITS comprises the two probes used. The probe-to-read distance for the vast majority of reads is 0-60 bp, confirming that reads were captured by their expected probes.
