# Supplementary material for: Spatial metatranscriptomics resolves host–bacteria–fungi interactomes
Source: Nat Biotechnol. 2023 Nov 20;42(9):1384–93. doi: 10.1038/s41587-023-01979-2 (PMC11392817; doi:10.1038/s41587-023-01979-2)
Supplement: Supplementary file 2 — Reporting Summary [file 41587_2023_1979_MOESM2_ESM.pdf]

nature portfolio

Corresponding author(s): Stefania Giacomello

Last updated by author(s): Sep 4, 2023

Reporting Summary

Nature Portfolio wishes to improve the reproducibility of the work that we publish. This form provides structure for consistency and transparency in reporting. For further information on Nature Portfolio policies, see our Editorial Policies and the Editorial Policy Checklist.

Statistics

For all statistical analyses, confirm that the following items are present in the figure legend, table legend, main text, or Methods section.

n/a	Confirmed	
☐	☒	The exact sample size (*n*) for each experimental group/condition, given as a discrete number and unit of measurement
☐	☒	A statement on whether measurements were taken from distinct samples or whether the same sample was measured repeatedly
☐	☒	The statistical test(s) used AND whether they are one- or two-sided<br>*Only common tests should be described solely by name; describe more complex techniques in the Methods section.*
☐	☒	A description of all covariates tested
☐	☒	A description of any assumptions or corrections, such as tests of normality and adjustment for multiple comparisons
☐	☒	A full description of the statistical parameters including central tendency (e.g. means) or other basic estimates (e.g. regression coefficient) AND variation (e.g. standard deviation) or associated estimates of uncertainty (e.g. confidence intervals)
☐	☒	For null hypothesis testing, the test statistic (e.g. *F*, *t*, *r*) with confidence intervals, effect sizes, degrees of freedom and *P* value noted<br>*Give P values as exact values whenever suitable.*
☐	☒	For Bayesian analysis, information on the choice of priors and Markov chain Monte Carlo settings
☐	☒	For hierarchical and complex designs, identification of the appropriate level for tests and full reporting of outcomes
☐	☒	Estimates of effect sizes (e.g. Cohen's *d*, Pearson's *r*), indicating how they were calculated

Our web collection on statistics for biologists contains articles on many of the points above.

Software and code

Policy information about availability of computer code

Data collection

For the whole leaves infected with bacteria ZEN 2.1 software package was used for the bright field and fluorescent image acquisition.
Bright field images for all the sections were acquired using Metafer Slide Scanning system (v. 3.14.2, Metasystems).
The fluorescent signal for for tissue optimization experiment was acquired using Mapix image analysis software (v. 9.1.0, Innopsys).

Data analysis

The novel probe sequences were designed using the SILVA 16S database (v138.1) using CDHIT (v4.8.1). Representative sequences were aligned using MAFFT (v7.245), and the sequence profile was plotted using weblogo (v3.7.5).

Raw reads were quality trimmed and filtered with cutadapt (v. 2.9) and spot locations were identified with Loupe Browser (v. 5.1.0). Gene expression matrices were generated by ST Pipeline (version 1.7.9) and aligned to Arabidopsis thaliana reference genome (TAIR10) with STAR (v. 2.7.7a). Within ST Pipeline the annotation was performed using htseq-count (v. 1.0) and the demultiplexing with Taggd (v. 0.3.6)

Taxonomic assignment of microbial reads was performed using usearch (v. 11.0.667) and MMseqs2 (version 1f30213) against the NCBI NT database [downloaded January 2021]. This was followed by applying the LCA algorithm part of TaxonKit (version 0.7.2) R package using the NCBI taxonomy database and followed by UMI filtering. The procedure is detailed at the 'Taxonomic assignment of microbial reads' section of the method. The result of this analysis is a spatial matrix with reads count assigned to each Bacterial/Fungi genus.

The probe concentration experiments and the experiments with fluorescently labeled Pseudomonas DC3000 were analyzed with R (version 4.0.5) and STUtility (v. 0.1.0), Seurat (v. 4.1.0, major dependence in ST Pipeline) and ggplot2 (v. 3.3.5). Microbial content was analyzed with vegan (v. 2.5-7). Correlation plots were generated with corrplot (v. 0.92). Matlab (2022a) and k-nearest-neighbor algorithm was used to extract the maximum fluorescent values per spot. R (v. 4.2.2) and Eulerr (v. 7.0.0) package were used to generate the venn-diagrams. Comparison between SmT and amp-seq was analyzed in R (version 4.0.3) using ggpairs function part of GGally package (v. 2.1.0).

Microbial hotspots (based on 16S rRNA/ITS reads) were analyzed in R (version 4.0.3) using the Getis-Ord G statistic as implemented in the localG function of the R spdep package (version 1.1.11). The p.adjustSP function of the R spdep package was used with the BH-FDR method to correct the G stats p-values while accounting for the number of neighbors of each region. Hotspot spatial maps were plotted using the R tmap package (v. 3.3-2).

Microbial interaction network were inferred based on the Spearman rank correlation coefficient (SRCC) corrected p-value (BH-FDR) values of the reads count associated with each pair of genera. The procedure to account also for the spatial organization of microbes in the array is described in the 'Microbial interaction network analysis' section of the methods section. The microbial network was calculated using R package igraph (v. 1.2.6) and visualized with R package ggraph (v. 2.0.5).

Host, bacterial and fungal modalities were visualized in R (version 4.0.5) and STUtility (v. 0.1.0) and Seurat (v. 4.1.0). Harmony (version 0.1.0). For host expression each section was normalized with Seurat (v. 4.1.0) function sctransform and Harmony (version 0.1.0). Markers were identified using Seurat's commands LogNormalize and FindAllMarkers. Spot cell type deconvolution for the host data was performed with Stereoscope (v. 0.3) and further visualized in R (v. 4.0.5) with STUtility (v. 0.1.0) and pheatmap (v. 1.0.12) packages. Host saturation points were calculated with STPipeline (v. 1.7.9) and visualised in R (v. 4.0.5) using ggplot2 (v. 3.3.5).

Host-response analyses was performed by applying the Boruta algorithm to determine which set of A. thaliana genes is important to explain the microbial load on each spot of the array. For this purpose we used the R (version 4.0.3) implementation of the Boruta package (v. 7.0.0) with 1000 trees. Overall, a gene was considered further if it was found to be significant by Boruta and if its SRCC p-value (after FDR correction) was below 0.01. GO enrichment analyses were performed with the DAVID webserver with the DAVID knowledgebase v2022q1. The procedure is detailed at the 'Host-response analyses' section of the method.

All internal scripts and code written to analyze the data and produce the figures of this manuscript are available on Github (https://github.com/giacomellolab/SpatialMetaTranscriptomics).

For manuscripts utilizing custom algorithms or software that are central to the research but not yet described in published literature, software must be made available to editors and reviewers. We strongly encourage code deposition in a community repository (e.g. GitHub). See the Nature Portfolio guidelines for submitting code & software for further information.

Data

Policy information about availability of data

All manuscripts must include a data availability statement. This statement should provide the following information, where applicable:

- Accession codes, unique identifiers, or web links for publicly available datasets
- A description of any restrictions on data availability
- For clinical datasets or third party data, please ensure that the statement adheres to our policy

All sequencing data have been deposited at NCBI-SRA under the Bioproject PRJNA784452. Source data files (bright field images, alignment matrices, putative microbial reads and annotation files, and gene/taxa matrices) have been deposited to Zenodo (DOI:). Scripts written for the analyses described in this paper are available on Github (https://github.com/giacomellolab/SpatialMetaTranscriptomics).

Human research participants

Policy information about studies involving human research participants and Sex and Gender in Research.

Reporting on sex and gender	n/a
Population characteristics	n/a
Recruitment	n/a
Ethics oversight	n/a

Note that full information on the approval of the study protocol must also be provided in the manuscript.

Field-specific reporting

Please select the one below that is the best fit for your research. If you are not sure, read the appropriate sections before making your selection.

☒ Life sciences ☐ Behavioural & social sciences ☐ Ecological, evolutionary & environmental sciences

For a reference copy of the document with all sections, see nature.com/documents/nr-reporting-summary-flat.pdf

Life sciences study design

All studies must disclose on these points even when the disclosure is negative.

Sample size	The sample size for outdoor-grown Arabidopsis thaliana samples was determined by sample availability:

Sample size	- The proof-of-concept experiment with fluorescently labelled Pseudomonas DC3000 was conducted on 2 biological replicates.<br>- The validation experiment comparing amp-seq vs SmT was conducted on 4 biological replicates.<br>- The probe concentration experiments were conducted on 3 leaves with 4 sections from each leaf for different probe concentrations.<br>- The axenically-grown leaves derive from experiments from 8 leaves one section from each, profiled in two separate arrays.<br>- The detection of microbial populations and analysis of A. thaliana gene expression patterns were carried out on a total of 42,389 capture spots deriving from 13 tissue sections across 4 leaves of 2 different plants.
Data exclusions	No data was excluded from the analysis.
Replication	Multiple plants and leaves and sections were included to enhance the reproducibility of the method. Multiple consecutive sections from each leaf sample were run with the same experimental conditions, where possible, to enhance the reproducibility of the approach.<br><br>- The proof-of-concept experiment with fluorescently labelled Pseudomonas DC3000 was conducted on two biological replicates on two multimodal capture areas.<br>- The validation experiment comparing amp-seq vs SmT was conducted on four biological replicates on multimodal SmT arrays and compared to one (Leaf C) or two (Leaves A, B, D) primer pairs of amplicon sequencing.<br>- The probe concentration experiments were conducted on three leaves with four sections from each leaf for different probe concentrations all providing expected results.<br>- The axenically-grown leaves derive from experiments from 8 leaves one section from each, profiled in two separate arrays and compared with crude RNA extracts from the same leaf batches on two separate arrays.<br>- The detection of microbial populations and analysis of A. thaliana gene expression patterns were carried out on 13 tissue sections across 4 leaves of 2 different plants. Cell type proportions per spot were calculated to identify similar regions from the tissue sections in an unsupervised manner.<br><br>The attempts at replication were successful.
Randomization	Each experiment was processed in their own batch since Spatial metaTranscriptomics allows us to analyze all the samples (leaf sections) and the spatial locations (spots) in the same manner, using the same criteria. Comparisons between the methods were done for all the data without exclusions in an unsupervised manner so randomization was not needed.
Blinding	The researchers were not blinded as the results did not include subjective measurements and were performed by individual researchers. However, all analyses were performed independently on each sample (leaf section). Comparisons between the different leaves and plants studied were done only in retrospect.

Reporting for specific materials, systems and methods

We require information from authors about some types of materials, experimental systems and methods used in many studies. Here, indicate whether each material, system or method listed is relevant to your study. If you are not sure if a list item applies to your research, read the appropriate section before selecting a response.

Materials & experimental systems

n/a	Involved in the study
☒	☐ Antibodies
☒	☐ Eukaryotic cell lines
☒	☐ Palaeontology and archaeology
☒	☐ Animals and other organisms
☒	☐ Clinical data
☒	☐ Dual use research of concern

Methods

n/a	Involved in the study
☒	☐ ChIP-seq
☒	☐ Flow cytometry
☒	☐ MRI-based neuroimaging
